# Supplementary material for: Selective agonist of TRPML2 reveals direct role in chemokine release from innate immune cells
Source: eLife. 2018 Nov 27;7:e39720. doi: 10.7554/eLife.39720 (PMC6257821; doi:10.7554/eLife.39720)
Supplement: Supplementary file 1. [file elife-39720-supp1.doc]

**Supplementary File 1.** Synthesis details and analytical data

NMR spectra were recorded on an Avance III HD 400 MHz Bruker BioSpin (^1^H-NMR: 400 MHz, ^13^C-NMR: 101 MHz) and an Avance III HD 500 MHz Bruker BioSpin (^1^H-NMR: 500 MHz, ^13^C-NMR: 126 MHz) spectrometer, respectively. NMR-Spectra were recorded in deuterated solvents and chemical shifts are reported in δ (ppm) relative to the internal standard tetramethylsilane (TMS) or the solvent peak, respectively. *J* values are given in hertz. Multiplicities are abbreviated as follows: s = singlet, d = doublet, t = triplet, q = quartet, m = multiplet. Signal assignments were carried out based on ^1^H, ^13^C, HMBC, HMQC and COSY spectra. NMR spectra were analyzed with the NMR software MestReNova, Version 5.1.1-3092 (Mestrelab Research S.L.). Except as noted otherwise, NMR spectra were recorded at room temperature. Mass spectra were obtained on a JMS GCmate II Jeol by electron ionization (EI), a JMS-700 MStation Jeol (EI), and a MAT 95 and MAT 90 Thermo Finnigan. IR measurements were carried out with a Perkin-Elmer FTIR Paragon 1000 spectrometer and a Jasco FT/IR-4100 with ATR PRO450-S respectively. Melting points were determined by the open tube capillary method on a Büchi melting point B-450 apparatus and are uncorrected. HPLC purities were determined using a HP Agilent 1100 HPLC with a diode array detector and an Agilent Poroshell column (120 EC-C18; 3.0 × 100 mm; 2.7 Micron) with acetonitrile/water as eluent. All chemicals used were of analytical grade and no further puriﬁcation was needed. THF and dioxane were dried over sodium and distilled before usage, all other solvents were purified by distillation. All reactions were monitored by thin-layer chromatography (TLC) using pre-coated plastic sheets POLYGRAM® SIL G/UV254 from Macherey-Nagel (Düren, Germany). Compounds on TLC plates were detected under UV light at 254 and 366 nm. Flash column chromatography was performed on Merck silica gel Si 60 (0.015 – 0.040 mm).

Arylaldoximes were prepared from aldehydes according to a known procedure (McIntosh et al, 2012).

Mesitonitrile oxide was prepared from mesitaldehyde oxime according to the protocol of Grundmann et al (1970).

2,2,4-Trimethyl-1,2-dihydroquinoline was prepared from aniline and acetone according to a protocol published by Chen et al (2007). Hydrogenation of 2,2,4-trimethyl-1,2-dihydroquinoline to 2,2,4-trimethyl-1,2,3,4-tetrahydroquinoline was performed according to patent PCT Int. Appl. 2010109301 (Venturini et al, 2010).

N-Phthaloyl amino acids were prepared from phthalic anhydride and the corresponding ω-aminocarboxylic acids according to known procedures (Tan et al, 2004; Zav’yalov et al, 2002).

The following compounds were prepared according to literature: EVP-7 (Mendelsohn et al, 2012), EVP-10 (Medelsohn et al, 2012), EVP-17 (Xiang et al, 2014; Li et al, 2015), EVP-37 (Zlatopolskiy et al, 2012), EVP-69 (Orth et al, 2010; Kociolek et al, 2004; Caldirola et al, 1986), EVP-75 (McIntosh et al, 2012; Lee et al, 2004).

**General Procedures:**

Details concerning used equivalents and eluents for chromatography are given below for each single compound.

**General procedure 1: Huisgen cycloaddition of norbornene with *in situ* prepared nitrile oxides**[Bis(trifluoroacetoxy)iodo]benzene (PIFA) (1.2 eq.) was added slowly to a stirred solution of arylaldoxime (1.0  eq.) and norbornene (1.5 eq.) in methanol/water (2:1, 2 mL/mmol aldoxime). After stirring for 30 minutes at ambient temperature, 50 mL water were added and the mixture was extracted with diethyl ether (3 × 50 mL). The combined organic layers were washed with brine, dried over sodium sulfate, filtered and concentrated. The crude product was purified by flash column chromatography (FCC).

**General procedure 2: Huisgen cycloaddition of alkenes with *in situ* prepared mesitonitrile oxide**[Bis(trifluoroacetoxy)iodo]benzene (PIFA) (1.2 eq.) was added slowly to a stirred solution of mesitaldehyde oxime (1.0 eq.) and alkene (1.5 eq.) in methanol/water (2:1, 2 mL/mmol mesitaldehyde oxime). After stirring for 20 hours at ambient temperature, 50 mL water were added and the mixture was extracted with diethyl ether (3 × 50 mL). The combined organic layers were washed with brine, dried over sodium sulfate, filtered and concentrated. The crude product was purified by flash column chromatography (FCC).

**General procedure 3: Huisgen cycloaddition with mesitonitrile oxide**A solution of mesitonitrile oxide and the appropriate alkene or alkyne in ethyl acetate (3 mL/mmol mesitaldehyde oxime) was stirred for 15 hours at ambient temperature.
**Workup a)** 20 mL water were added and the mixture was extracted with ethyl acetate (3 × 20 mL). The combined organic layers were washed with brine, dried over magnesium sulfate, filtered and concentrated. The crude product was purified by flash column chromatography (FCC).
**Workup b)** The volatiles were removed under reduced pressure and the residue was purified by flash column chromatography.

**General procedure 4: Huisgen cycloaddition of enolates with mesitonitrile oxide**The appropriate ketone (dissolved in minimum amounts of dry THF in case of solid ketones) (1-3 eq.) was added dropwise to a solution of lithium diisopropylamide (1.0 eq.) in 4 mL dry THF at -78 °C under nitrogen atmosphere. The solution was stirred at -78 °C for 2 hours before mesitonitrile oxide (1.0 eq.), dissolved in 2 mL dry THF, was added dropwise. The mixture was allowed to reach room temperature slowly over 2 hours under stirring. Reaction progress was controlled by TLC. After consumption of the nitrile oxide, a saturated aqueous solution of ammonium chloride (20 mL) was added and the mixture was extracted with ethyl acetate (3 × 30 mL). The combined organic layers were washed with brine, dried over sodium sulfate, filtered and concentrated. The crude product was purified as given below for any single compound.

**General procedure 5: Dehydration of 5-hydroxyisoxazolines to isoxazoles**A solution of sodium carbonate (5 eq.) in 2 mL water was added to a stirred solution of the appropriate 5-hydroxyisoxazoline in methanol (5 mL/mmol). The mixture was refluxed for 2 hours, then the methanol was evaporated *in vacuo*. The resulting aqueous solution was diluted with water (20 mL) and extracted with dichloromethane (3 × 20 mL). The combined organic layers were washed with brine, dried over sodium sulfate, filtered and the solvent was evaporated *in vacuo* to give pure isoxazole.

**General procedure 6: Amide formation with *N*-phthalylglycyl chloride**Triethylamine (0.2 mL/mmol) was added to a stirred solution of secondary amine in dichloromethane (1 mL/mmol). While stirring at ambient temperature *N*-phthalylglycyl chloride (1.0 eq.) was added slowly and the mixture stirred for 15 hours. Dichloromethane (20 mL) was added and the mixture washed with hydrochloric acid (2 M, 3 × 20 mL) and sodium hydroxide solution (2 M, 3 × 20 mL). The combined organic layers were dried over magnesium sulfate, filtered and concentrated.
**Workup a)** The residue was purified by flash column chromatography (FCC).
**Workup b)** Crystallization from hexanes resulted in the pure product.

**General procedure 7: *N*-Alkylation of imides with EVP-175**A mixture of (*RS*)-2-bromo-1-(2,2,4-trimethyl-3,4-dihydroquinolin-1(2*H*)-yl)ethan-1-one **(EVP-175)**, the appropriate imide (1-2 eq.), 3.0 g dried molecular sieves 4 Å and sodium iodide (1.0 eq.) was disperged in dry THF (4 mL/mmol **EVP-175**) in a dry flask under nitrogen atmosphere. Under stirring at ambient temperature LiHMDS (1 M in toluene, 1.0 eq.) was added slowly and the resulting suspension was heated to 70 °C for 15 hours. After cooling to room temperature the molecular sieves was removed by filtration and washed with dichloromethane. The organic phase was washed with water and brine, dried over sodium sulfate, filtered and concentrated. The crude product was purified by flash column chromatography (FCC).

**General procedure 8: *N*-Acylation of 2,2,4-trimethyl-1,2,3,4-tetrahydroquinoline**A solution of the appropriate carboxylic acid in thionyl chloride was refluxed for one hour, then excess thionyl chloride was removed by purging with a flow of nitrogen (gas stream passed through a sodium hydroxide solution). The resulting acyl chloride was dried in vacuum and dissolved in dichloromethane without further purification. Under cooling in an ice bath a solution of 2,2,4-trimethyl-1,2,3,4-tetrahydroquinoline in 1 mL dichloromethane was added over 30 min. The mixture was stirred for another 0.5 – 15 hours while slowly warming to room temperature, soaked on Isolute® and purified by flash column chromatography using hexanes/ethyl acetate 4:1 as eluent.

**(3a*RS*,4*RS*,7*SR*,7a*RS*)-3-Mesityl-3a,4,5,6,7,7a-hexahydro-4,7-methanobenzo[*d*]isoxazole (SN-2)**

C_17_H_21_NO, M_r_ = 255.36

Prepared following general procedure 1 using mesitaldehyde oxime (520 mg, 3.19 mmol) and norbornene (458 mg, 4.86 mmol); FCC using hexanes/ethyl acetate 4:1 gave colorless crystals (87 %). mp: 98 °C; ^1^H-NMR (500 MHz, CDCl_3_) δ (ppm) = 6.89 (s, 2H, 3'-H, 5'-H), 4.64 (dt, *J_1_* = 8.3 Hz, *J_2_* = 1.3 Hz, 1H, 7a-H), 3.26 (d, *J* = 8.3 Hz, 1H, 3a-H), 2.67 (d, *J* = 4.9 Hz, 1H, 7-H), 2.29 (s, 3H, 4'-CH_3_), 2.27 (s, 6H, 2'‑CH_3_, 6'-CH_3_), 2.28 – 2.24 (m, 1H, 4-H), 1.81 (br. d, *J* = 10.4 Hz, 1H, 8-H), 1.63 – 1.50 (m, 1H, 6-H), 1.53 – 1.43 (m, 1H, 5-H), 1.28 (br. d, *J* = 10.4 Hz, 1H, 8-H), 1.18 – 1.10 (m, 2H, 5-H, 6-H). ^13^C-NMR (101 MHz, CDCl_3_) δ (ppm) = 157.8 (C-3), 138.4 (C-4'), 136.7 (C-2', C-6'), 128.7 (C-3', C-5'), 126.4 (C-1'), 86.8 (C-7a), 61.3 (C-3a), 42.7 (C-7), 38.8 (C-4), 32.8 (C-8), 27.6 (C-5), 22.8 (C-6), 21.0 (4'-CH_3_), 20.2 (2'-CH_3_, 6'-CH_3_). IR (KBr)
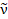
 [cm^-1^] = 2963, 2928, 2880, 2870, 1610, 1458, 1438, 1377, 1326, 1314, 1213, 1034, 917, 899, 869, 849, 819. MS (EI) *m/z* (%) = 255 (100) [M^•^]^+^, 252 (95), 250 (60), 218 (60), 181 (73), 169 (85). HRMS (EI) 255.1594 (calculated for C_17_H_21_NO: 255.1623). Purity (HPLC) > 99 % (λ = 210 nm), > 99 % (λ = 254 nm).

**4-((3a*RS*,4*RS*,7*SR*,7a*RS*)-3a,4,5,6,7,7a-Hexahydro-4,7-methanobenzo[*d*]isoxazol-3-yl)-*N*,*N*-dimethylaniline (EVP-6)**

C_16_H_20_N_2_O, M_r_ = 256.35

To a stirred solution of 4-(dimethylamino)benzaldehyde oxime (281 mg, 1.71 mmol) and norbornene (238 mg, 2.52 mmol) in 5 mL DMF at 0 °C, NBS (382 mg, 2.15 mmol) and triethylamine (2 mL) were added. The mixture was stirred for 1 h, diluted with water (50 mL) and extracted with diethyl ether (3 × 50 mL). The combined organic layers were dried over sodium sulfate, filtered and concentrated. The crude product was purified by flash column chromatography (FCC) using hexanes/ethyl acetate 4:1 gave yellow crystals (12 %). mp: 149 °C; ^1^H-NMR (500 MHz, CDCl_3_) δ (ppm) = 7.59 (d, *J* = 8.9 Hz, 2H, 3-H, 5-H), 6.69 (d, *J* = 8.9 Hz, 2H, 2-H, 6-H), 4.56 (d, *J* = 8.4 Hz, 1H, 7a'-H), 3.46 (d, *J* = 8.4, 1H, 3a'-H), 3.00 (s, 6H, N(CH_3_)_2_), 2.61 – 2.57 (m, 1H, 4'-H), 2.54 – 2.50 (m, 1H, 7'-H), 1.60 – 1.51 (m, 3H, 5'-H, 6'-H, 8'-H), 1.37 – 1.31 (m, 1H, 5'-H), 1.20 – 1.12 (m, 2H, 6'‑H, 8'-H). ^13^C-NMR (101 MHz, CDCl_3_) δ (ppm) = 156.7 (C-3'), 151.2 (C-1), 128.1 (C-3, C-5), 116.9 (C-4), 111.8 (C-2, C‑6), 87.1 (C-7a'), 57.6 (C-3a'), 43.0 (C-4'), 40.2 (N(CH_3_)_2_), 39.4 (C‑7'), 32.3 (C‑8'), 27.4 (C-5'), 22.7 (C-6'). IR (KBr)
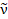
 [cm^-1^] = 3433, 2959, 2873, 2804, 1613, 1529, 1369, 1198, 982, 888, 878, 865, 815. MS (EI) *m/z* (%) = 256 (100) [M^•^]^+^, 252 (31), 250 (24), 145 (20). HRMS (EI) 256.1575 (calculated for C_16_H_20_N_2_O: 256.1576). Purity (HPLC) 99 % (λ = 210 nm), > 99 % (λ = 254 nm).

**(3a*RS*,4*RS*,7*SR*,7a*RS*)-3-(2,4,6-Trimethoxyphenyl)-3a,4,5,6,7,7a-hexahydro-4,7-methanobenzo[*d*]isoxazole (EVP-11)**

C_17_H_21_NO_4_, M_r_ = 303.36

Prepared following general procedure 1 using 2,4,6-trimethoxybenzaldehyde oxime (0.20 g, 0.95 mmol) and norbornene (0.13 g, 1.4 mmol). FCC using dichloromethane/methanol 99:1 gave colorless crystals (33 %). mp: 192 °C; ^1^H-NMR (400 MHz, CDCl_3_) δ (ppm) = 6.13 (s, 2H, 3'-H, 5'-H), 4.56 (dt, *J_1_* = 8.4 Hz, *J_2_* = 1.2 Hz, 1H, 7a-H), 3.83 (s, 3H, 4'‑O‑CH_3_), 3.78 (s, 6H, 2'‑O‑CH_3_, 6'‑O‑CH_3_), 3.44 (dt, *J_1_* = 8.4 Hz, *J_2_* = 0.9 Hz, 1H, 3a-H), 2.58 (d, *J* = 4.2 Hz, 1H, 7-H), 2.14 (d, *J* = 3.5 Hz, 1H, 4‑H), 1.81 (br. d, *J* = 10.5 Hz, 1H, 8‑H), 1.53 – 1.40 (m, 2H, 5-H, 6-H), 1.18 – 1.07 (m, 3H, 5-H, 6-H, 8-H). ^13^C-NMR (101 MHz, CDCl_3_) δ (ppm) = 162.2 (C-4'), 159.6 (C-2', C-6'), 153.5 (C-3), 100.5 (C-1'), 90.7 (C-3', C-5'), 86.6 (C-7a), 60.3 (C-3a), 55.9 (2'‑O‑CH_3_, 6'‑O‑CH_3_), 55.4 (4'‑O‑CH_3_), 43.0 (C-7), 38.6 (C-4), 32.5 (C-8), 27.2 (C-5), 23.1 (C-6). IR (KBr)
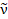
 [cm^-1^] = 3108, 3083, 2998, 2967, 2950, 2925, 2869, 2842, 1615, 1596, 1585, 1503, 1468, 1454, 1420, 1332, 1234, 1208, 1162, 1123, 1072, 1030, 856, 825. MS (EI) *m/z* (%) = 303 (100) [M^•^]^+^, 209 (80). HRMS (EI) 303.1480 (calculated for C_17_H_21_NO_4_: 303.1471). Purity (HPLC) > 99 % (λ = 210 nm), > 99 % (λ = 254 nm).

**(3a*RS*,4*RS*,7*SR*,7a*RS*)-3-(4-(Trifluoromethyl)phenyl)-3a,4,5,6,7,7a-hexahydro-4,7-methanobenzo[*d*]isoxazole (EVP-14)**

C_15_H_14_F_3_NO, M_r_ = 281.28

Prepared following general procedure 1 using 4-trifluoromethylbenzaldehyde oxime (0.20 g, 1.1 mmol) and norbornene (0.15 g, 1.6 mmol). FCC using hexanes/ethyl acetate 85:15 and crystallization from methanol gave colorless crystals (45 %). mp: 121 °C; ^1^H-NMR (500 MHz, CDCl_3_) δ (ppm) = 7.83 (d, *J* = 8.2 Hz, 2H, 2'-H, 6'-H), 7.65 (d, *J* = 8.2 Hz, 2H, 3'-H, 5'-H), 4.70 (dt, *J_1_* = 8.3 Hz, *J_2_* = 1.4 Hz, 1H, 7a-H), 3.50 (d, *J* = 8.2 Hz, 1H, 3a-H), 2.67 – 2.63 (m, 1H, 7-H), 2.53 – 2.48 (m, 1H, 4-H), 1.66 – 1.58 (m, 2H, 5-H, 6‑H), 1.51 (br. d, *J* = 10.7 Hz, 1H, 8-H), 1.41 – 1.35 (m, 1H, 5-H), 1.24 – 1.17 (m, 2H, 6-H, 8-H). ^13^C-NMR (126 MHz, CDCl_3_) δ (ppm) = 156.0 (C-3), 132.9 (C-1'), 131.3 (q, *J* = 32.6 Hz, C-4'), 127.0 (C‑2', C-6'), 125.6 (q, *J* = 3.9 Hz, C-3', C-5'), 123.9 (q, *J* = 273.2 Hz, CF_3_), 88.5 (C‑7a), 56.6 (C-3a), 43.0 (C-7), 39.2 (C-4), 32.3 (C-8), 27.4 (C-5), 22.7 (C‑6). IR (KBr)
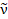
 [cm^-1^] = 2972, 2938, 2878, 1326, 1169, 1122, 1109, 1071, 841. MS (EI) *m/z* (%) = 281 (100) [M^•^]^+^, 214 (55). HRMS (EI) 281.1022 (calculated for C_15_H_14_F_3_NO: 281.1028). Purity (HPLC) > 99 % (λ = 210 nm), > 99 % (λ = 254 nm).

**(3a*RS*,4*RS*,7*SR*,7a*RS*)-3-(4-Chlorophenyl)-3a,4,5,6,7,7a-hexahydro-4,7-methanobenzo[*d*]isoxazole (EVP-19)**

C_14_H_14_ClNO, M_r_ = 247.72

A solution of 4-chlorobenzaldehyde oxime (571 mg; 3.67 mmol) in methanol (2 mL) and trifluoroacetic acid (15 µL) was added slowly at room temperature to a stirred suspension of (diacetoxyiodo)benzene (DIB) (1.58 g; 4.92 mmol) and norbornene (375 mg; 3.98 mmol) in methanol (6 mL) and trifluoroacetic acid (45 µL). The mixture was stirred for 30 min, then diluted with water and extracted with ethyl acetate (3 × 30 mL). The combined organic layers were washed with brine, dried over sodium sulfate, filtered and concentrated in vacuo. The crude product was crystallized from methanol to give colorless crystals (17 %). mp: 78 °C; ^1^H-NMR (500 MHz, CDCl_3_) δ (ppm) = 7.66 – 7.62 (m, 2H, 2'-H, 6'-H), 7.39 – 7.35 (m, 2H, 3'-H, 5'-H), 4.65 (d, *J* = 8.4 Hz, 1H, 7a-H), 3.45 (d, *J* = 8.3 Hz, 1H, 3a-H), 2.66 – 2.61 (m, 1H, 7-H), 2.50 – 2.47 (m, 1H, 4-H), 1.61 – 1.55 (m, 2H, 5-H, 6-H), 1.50 (br. d, *J* = 10.6 Hz, 1H, 8-H), 1.38 – 1.32 (m, 1H, 5-H), 1.22 – 1.15 (m, 2H, 6-H, 8-H). ^13^C-NMR (126 MHz, CDCl_3_) δ (ppm) = 156.0 (C-3), 135.6 (C-4'), 128.9 (C-3', C-5'), 128.0 (C-2', C-6'), 127.9 (C-1'), 88.2 (C-7a), 56.9 (C-3a), 43.0 (C-7), 39.2 (C-4), 32.3 (C-8), 27.4 (C-5), 22.7 (C-6). IR (KBr)
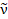
 [cm^-1^] = 3075, 2964, 2948, 2871, 1591, 1492, 1402, 1348, 1089, 921, 910, 888, 860, 828, 818. MS (EI) *m/z* (%) = 247 (100) [M^•^]^+^, 191 (40), 151 (28). HRMS (EI) 247.0748 (calculated for C_14_H_14_^35^ClNO 247.0764). Purity (HPLC) > 99 % (λ = 210 nm), > 99 % (λ = 254 nm).

**(3a*RS*,7a*RS*)-3-Mesityl-3a,4,5,6,7,7a-hexahydrobenzo[*d*]isoxazole (EVP-20)**

C_16_H_21_NO, M_r_ = 243.35

Prepared following general procedure 2 using mesitaldehyde oxime (392 mg, 2.40 mmol) and cyclohexene (688 mg, 8.38 mmol). FCC using hexanes/ethyl acetate 4:1 gave colorless crystals (13 %). mp: 80 °C; ^1^H-NMR (500 MHz, CDCl_3_) δ (ppm) = 6.90 (s, 2H, 3'-H, 5'-H), 4.60 (dt, *J_1_* = 7.6 Hz, *J_2_* = 4.9 Hz, 1H, 7a-H), 3.28 – 3.22 (m, 1H, 3a-H), 2.30 (s, 6H, 2'-CH_3_, 6'-CH_3_), 2.29 (s, 3H, 4'-CH_3_), 2.09 – 2.02 (m, 1H, 7-H), 1.88 – 1.80 (m, 1H, 7-H), 1.66 – 1.57 (m, 2H, 4-H, 6-H), 1.54 – 1.40 (m, 3H, 4-H, 5-H, 6-H), 1.28 – 1.19 (m, 1H, 5-H). ^13^C-NMR (126 MHz, CDCl_3_) δ (ppm) = 163.7 (C-3), 138.5 (C-4'), 136.8 (C-2', C-6'), 128.8 (C-3', C-5'), 126.2 (C-1'), 79.1 (C-7a), 48.9 (C-3a), 25.9 (C-7), 24.3 (C-4), 22.4 (C-5), 21.1 (4'-CH_3_), 20.8 (C-6), 20.4 (2'-CH_3_, 6'-CH_3_). IR (KBr)
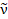
 [cm^-1^] = 2934, 2860, 1612, 1448, 1376, 1325, 938, 851. MS (EI) 243.1624 (calculated for C_16_H_21_NO: 243.1623). HRMS (EI) 243.1624 (calculated for C_16_H_21_NO: 243.1623). Purity (HPLC) 97 % (λ = 210 nm), 98 % (λ = 254 nm).

**3-Mesityl-4,5,6,7-tetrahydrobenzo[*d*]isoxazole (EVP-21)**

C_16_H_19_NO, M_r_ = 241.33

Prepared following general procedure 5 using (3a*RS*,7a*RS*)-3-mesityl-4,5,6,7-tetrahydrobenzo[*d*]isoxazole-7a(3a*H*)-ol **(EVP‑24)** (181 mg, 0.699 mmol) to give a colorless oil (95 %); ^1^H-NMR (400 MHz, CDCl_3_) δ (ppm) = 6.93 – 6.90 (m, 2H, 3'-H, 5'-H), 2.77 (tt, *J_1_* = 6.4 Hz, *J_2_* = 1.5 Hz, 2H, 7-H), 2.31 (s, 3H, 4'-CH_3_), 2.16 (tt, *J_1_* = 6.0 Hz, *J_2_* = 1.6 Hz, 2H, 4-H), 2.08 (s, 6H, 2'-CH_3_, 6'-CH_3_), 1.95 – 1.84 (m, 2H, 6-H), 1.79 – 1.68 (m, 2H, 5-H). ^13^C-NMR (126 MHz, CDCl_3_) δ (ppm) = 168.1 (C-7a), 161.0 (C-3), 138.5 (C-4'), 137.1 (C-2', C-6'), 128.1 (C-3', C-5'), 125.8 (C-1'), 112.7 (C-3a), 22.8 (C-7), 22.6 (C-5), 22.3 (C-6), 21.2 (4'-CH_3_), 19.9 (2'‑CH_3_, 6'-CH_3_), 19.6 (C-4). IR (Film)
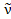
 [cm^-1^] = 3003, 2938, 2856, 1635, 1613, 1453, 1429, 1320, 1209, 1153, 986, 864, 851. MS (EI) *m/z* (%) = 241 (100) [M^•^]^+^, 185 (55), 184 (55). HRMS (EI) 241.1465 (calculated for C_16_H_19_NO: 241.1467). Purity (HPLC) > 99 % (λ = 210 nm), > 99 % (λ = 254 nm).

**(3a*RS*,4*RS*,7*SR*,7a*RS*)-3-(2,6-Dichlorophenyl)-3a,4,5,6,7,7a-hexahydro-4,7-methanobenzo[*d*]isoxazole (EVP-22 = ML2-SA1)**

C_14_H_13_Cl_2_NO, M_r_ = 282.16

Prepared following general procedure 1 using 2,6-dichlorobenzaldehyde oxime (0.20 g, 1.1 mmol) and norbornene (0.15 g, 1.6 mmol). FCC using hexanes/ethyl acetate 4:1 gave colorless crystals (68 %). mp: 116 °C; ^1^H-NMR (500 MHz, CDCl_3_) δ (ppm) = 7.39 – 7.35 (m, 2H, 3'-H, 5'-H), 7.29 – 7.25 (m, 1H, 4'-H), 4.73 (d, *J* = 8.4 Hz, 1H, 7a-H), 3.52 (d, *J* = 8.4 Hz, 1H, 3a-H), 2.68 (d, *J* = 4.6 Hz, 1H, 7-H), 2.34 (d, *J* = 4.1 Hz, 1H, 4-H), 1.96 (br. d, *J* = 10.6 Hz, 1H, 8-H), 1.64 – 1.56 (m, 1H, 6-H), 1.54 – 1.46 (m, 1H, 5-H), 1.29 (br. d, *J* = 10.6 Hz, 1H, 8-H), 1.22 – 1.12 (m, 2H, 5-H, 6-H). ^13^C-NMR (101 MHz, CDCl_3_) δ (ppm) = 154.6 (C-3), 135.2 (C-2', C-6'), 130.7 (C-4'), 129.1 (C-1'), 128.3 (C-3', C-5'), 88.1 (C-7a), 59.9 (C-3a), 42.5 (C-7), 39.0 (C-4), 33.0 (C-8), 27.4 (C-5), 22.9 (C-6). IR (KBr)
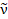
 [cm^-1^] = 3078, 2966, 2952, 2879,2868, 1579, 1557, 1427, 1325, 1192, 875, 785, 726. MS (EI) *m/z* (%) = 281 (100) [(^35^Cl_2_) M^•^]^+^, 216 (52), 214 (85), 185 (42). HRMS (EI) 281.0376 (calculated for C_14_H_13_^35^Cl_2_NO: 281.0374). Purity (HPLC) > 99 % (λ = 210 nm), > 99 % (λ = 254 nm).

**(3a*RS*,7a*RS*)-3-Mesityl-4,5,6,7-tetrahydrobenzo[*d*]isoxazol-7a(3a*H*)-ol (EVP-24)**

C_16_H_21_NO_2_, M_r_ = 259.35

Prepared following general procedure 4 using cyclohexanone (0.50 mL, 4.8 mmol) and mesitonitrile oxide (208 mg, 1.29 mmol). Washing the precipitate with hexanes gave a colorless solid (73 %). mp: 119 °C; ^1^H-NMR (400 MHz, CDCl_3_) δ (ppm) = 6.91 – 6.88 (m, 2H, 3'-H, 5'-H), 3.13 – 3.06 (m, 1H, 3a‑H), 2.70 (s, 1H, OH), 2.39 – 2.32 (m, 1H, 7-H), 2.33 (s, 6H, 2'-CH_3_, 6'-CH_3_), 2.29 (s, 3H, 4'-CH_3_), 1.92 – 1.81 (m, 2H, 4-H, 7-H), 1.81 – 1.72 (m, 1H, 6-H), 1.70 – 1.63 (m, 1H, 5-H), 1.53 – 1.40 (m, 1H, 6-H), 1.29 – 1.21 (m, 1H, 4-H), 1.21 – 1.12 (m, 1H, 5-H). ^13^C-NMR (126 MHz, CDCl_3_) δ (ppm) = 164.8 (C-3), 138.8 (C-4'), 137.3 (C-2', C-6'), 128.7 (C-3', C-5'), 125.7 (C-1'), 105.4 (C-7a), 54.9 (C-3a), 32.2 (C-7), 26.9 (C-4), 22.2 (C-6), 21.7 (C-5), 21.1 (4'-CH_3_), 20.18 (2'-CH_3_, 6'-CH_3_). IR (KBr)
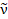
 [cm^-1^] = 3425, 2946, 2860, 1611, 1454, 1442, 1414, 1322, 1258, 1104, 1091, 958, 923, 860, 847, 828, 799. MS (EI) *m/z* (%) = 259 (45) [M^•^]^+^, 242 (52), 241 (100), 285 (62), 284 (67), 172 (70), 145 (62), 130 (55). HRMS (EI) 259.1567 (calculated for C_16_H_21_NO_2_: 259.1572). Purity (HPLC) > 99 % (λ = 210 nm), > 99 % (λ = 254 nm).

**(3a*RS*,4*RS*,7*SR*,7a*RS*)-3-(2-Nitrophenyl)-3a,4,5,6,7,7a-hexahydro-4,7-methanobenzo[*d*]isoxazole (EVP-26)**

C_14_H_14_N_2_O_3_, M_r_ = 258.28

Prepared following general procedure 1 using 2-nitrobenzaldehyde oxime (259 mg, 1.56 mmol) and norbornene (212 mg, 2.25 mmol). FCC using hexanes/ethyl acetate 4:1 gave a yellow oil (48 %); ^1^H-NMR (500 MHz, CDCl_3_) δ (ppm) = 8.04 (dd, *J_1_* = 7.7 Hz, *J_2_* = 1.3 Hz, 1H, 3'-H), 7.67 (ddd, *J_1_* = 7.7 Hz, *J_2_* = 7.7 Hz, *J_3_* = 1.3 Hz, 1H, 5'-H), 7.59 (ddd, *J_1_* = 7.7 Hz, *J_2_* = 7.7 Hz, *J_3_* = 1.5 Hz, 1H, 4'-H), 7.48 (dd, *J_1_* = 7.7 Hz, *J_2_* = 1.5 Hz, 1H, 6'-H), 4.73 (d, *J* = 8.3 Hz, 1H, 7a-H), 3.50 (d, *J* = 8.3 Hz, 1H, 3a-H), 2.64 (d, *J* = 4.3 Hz, 1H, 7-H), 2.15 (d, *J* = 3.6 Hz, 1H, 4-H), 1.64 (br. d, *J* = 10.6 Hz, 1H, 8-H), 1.58 – 1.47 (m, 2H, 5-H, 6-H), 1.25 (br. d, *J* = 10.6 Hz, 1H, 8-H), 1.25 – 1.12 (m, 2H, 5-H, 6-H). ^13^C-NMR (101 MHz, CDCl_3_) δ (ppm) = 155.7 (C-3), 148.1 (C-2'), 133.2 (C-5'), 131.3 (C-6'), 130.3 (C-4'), 125.5 (C‑1'), 124.8 (C-3'), 88.2 (C-7a), 58.7 (C-3a), 42.8 (C-7), 38.6 (C-4), 32.4 (C-8), 27.1 (C‑5), 22.7 (C-6). IR (Film)
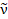
 [cm^-1^] = 2962, 2876, 1530, 1345, 922, 889, 850, 750, 741. MS (EI) *m/z* (%) = 258 (50) [M^•^]^+^, 191 (100). HRMS (EI) 258.1010 (calculated for C_14_H_14_N_2_O_3_: 258.1005). Purity (HPLC) > 99 % (λ = 210 nm), > 99 % (λ = 254 nm)

**(3a*RS*,4*RS*,7*SR*,7a*RS*)-3-(2-Methylphenyl)-3a,4,5,6,7,7a-hexahydro-4,7-methanobenzo[*d*]isoxazole (EVP-28)**

C_15_H_17_NO, M_r_ = 227.31

Prepared following general procedure 1 using 2-methylbenzaldehyde oxime (250 mg, 1.84 mmol) and norbornene (226 mg, 2.40 mmol). FCC using hexanes/ethyl acetate 4:1 gave a pale yellow oil (60 %); ^1^H-NMR (400 MHz, CDCl_3_) δ (ppm) = 7.37 (d, *J* = 7.2 Hz, 1H, 6'-H), 7.30 – 7.21 (m, 3H, 3'-H, 4'-H, 5'-H), 4.58 (d, *J*= 8.5 Hz, 1H, 7a-H), 3.57 (d, *J* = 8.4 Hz, 1H, 3a-H), 2.65 – 2.61 (m, 1H, 7-H), 2.51 (s, 3H, 2'-CH_3_), 2.36 – 2.33 (m, 1H, 4-H), 1.59 – 1.52 (m, 3H, 5-H, 6-H, 8-H), 1.34 – 1.26 (m, 1H, 5-H), 1.22 – 1.14 (m, 2H, 6-H, 8-H). ^13^C-NMR (126 MHz, CDCl_3_) δ (ppm) = 157.9 (C-3), 138.1 (C-2'), 131.5 (C-3'), 129.0 (C-4'), 128.8 (C-6'), 128.6 (C‑1'), 125.7 (C-5'), 86.5 (C-7a), 59.6 (C.3a), 43.2 (C-7), 39.0 (C-4), 32.4 (C-8), 27.3 (C‑5), 22.8 (C-6), 22.8 (2'-CH_3_). IR (Film)
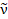
 [cm^-1^] = 3061, 3022, 2963, 2875, 1777, 1585, 1490, 1454, 1336, 1317, 987, 950, 921, 913, 887, 869, 769, 757, 720, 664. MS (EI) *m/z* (%) = 227 (100) [M^•^]^+^, 212 (33), 198 (50), 184 (30), 170 (37), 157 (36), 144 (36), 130 (40). HRMS (EI) 227.1288 (calculated for C_15_H_17_NO: 227.1310). Purity (HPLC) 98 % (λ = 210 nm), 99 % (λ = 254 nm).

**(3a*RS*,4*RS*,7*SR*,7a*RS*)-3-(3-Chlorophenyl)-3a,4,5,6,7,7a-hexahydro-4,7-methanobenzo[*d*]isoxazole (EVP-29)**

C_14_H_14_ClNO, M_r_ = 247.72

Prepared following general procedure 1 using 3-chlorobenzaldehyde oxime (256 mg, 1.61 mmol) and norbornene (173 mg, 1.83 mmol). FCC using hexanes/ethyl acetate 4:1 gave a colorless oil (44 %); ^1^H-NMR (400 MHz, CDCl_3_) δ (ppm) = 7.71 – 7.65 (m, 1H, 2'-H), 7.59 (ddd, *J_1_* = 7.3 Hz, *J_2_* = 1.8 Hz, *J_3_* = 1.6 Hz, 1H, 6'-H), 7.37 – 7.34 (m, 1H, 4'-H), 7.34 – 7.30 (m, 1H, 5'-H), 4.66 (d, *J* = 8.3 Hz, 1H, 7a‑H), 3.45 (d, *J* = 8.3 Hz, 2H, 3a-H), 2.71 – 2.57 (m, 1H, 7-H), 2.55 – 2.46 (m, 1H, 4-H), 1.67 – 1.56 (m, 2H, 5-H, 6-H), 1.50 (br. d, *J* = 10.7 Hz, 1H, 8-H), 1.42 – 1.30 (m, 1H, 5-H), 1.26 – 1.12 (m, 2H, 6-H, 8-H). ^13^C-NMR (101 MHz, CDCl_3_) δ (ppm) = 156.0 (C-3), 134.7 (C-3'), 131.3 (C-1'), 130.0 (C-5'), 129.7 (C-4'), 126.7 (C‑2'), 124.9 (C-6'), 88.3 (C-7a), 56.7 (C-3a), 43.0 (C-7), 39.2 (C-4), 32.3 (C-8), 27.4 (C-5), 22.7 (C-6). IR (Film)
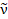
 [cm^-1^] = 3071, 2963, 2875, 1588, 1555, 1474, 1454, 1426, 1355, 1341, 1317, 1244, 1095, 1080, 989, 950, 924, 913, 898, 786, 686. MS (EI) *m/z* (%) = 247 (100) [(^35^Cl) M^•^]^+^, 180 (40). HRMS (EI) 247.0764 (calculated for C_14_H_14_^35^ClNO: 247.0764). Purity (HPLC) 97 % (λ = 210 nm), 98 % (λ = 254 nm).

**(3a*RS*,4*RS*,7*SR*,7a*RS*)-3-(2,4-Dichlorophenyl)-3a,4,5,6,7,7a-hexahydro-4,7-methanobenzo[*d*]isoxazole (EVP-31)**

C_14_H_13_Cl_2_NO, M_r_ = 282.16

Prepared following general procedure 1 using 2,4-dichlorobenzaldehyde oxime (251 mg, 1.32 mmol) and norbornene (188 mg, 2.00 mmol). FCC using hexanes/ethyl acetate 4:1 gave colorless crystals (49 %). mp: 70 °C; ^1^H-NMR (500 MHz, CDCl_3_) δ (ppm) = 7.46 (d, *J* = 8.3 Hz, 1H, 6'-H), 7.45 (d, *J* = 2.2 Hz, 1H, 3'-H), 7.27 (dd, *J_1_* = 8.3 Hz, *J_2_* = 2.2 Hz, 1H, 5'-H), 4.66 (d, *J* = 8.5 Hz, 1H, 7a-H), 3.82 (d, *J* = 8.5 Hz, 1H, 3a-H), 2.61 (br. s, 1H, 7-H), 2.19 (br. s, 1H, 4-H), 1.58 – 1.49 (m, 3H, 5‑H, 6-H, 8-H), 1.31 – 1.22 (m, 1H, 5-H), 1.22 – 1.19 (m, 1H, 8-H), 1.19 – 1.12 (m, 1H, 6-H). ^13^C-NMR (101 MHz, CDCl_3_) δ (ppm) = 156.2 (C-3), 135.9 (C-4'), 133.5 (C-2'), 131.7 (C-6'), 130.3 (C-3'), 127.5 (C‑1'), 127.3 (C-5'), 88.0 (C-7a), 58.5 (C-3a), 43.0 (C-7), 38.6 (C-4), 32.2 (C-8), 27.1 (C‑5), 22.8 (C-6). IR (KBr)
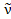
 [cm^-1^] = 3424, 3091, 3072, 2962, 2945, 2869, 1586, 1577, 1471, 1380, 1334, 1103, 1079, 987, 919, 892, 861, 839, 796. MS (EI) *m/z* (%) = 281 (100) [(^35^Cl_2_) M^•^]^+^, 214 (53). HRMS (EI) 281.0370 (calculated for C_14_H_13_^35^Cl_2_NO: 281.0374). Purity (HPLC) > 99 % (λ = 210 nm), 98 % (λ = 254 nm).

**(3a*RS*,4*RS*,7*SR*,7a*RS*)-3-(4-Nitrophenyl)-3a,4,5,6,7,7a-hexahydro-4,7-methanobenzo[*d*]isoxazole (EVP-33)**

C_14_H_14_N_2_O_3_, M_r_ = 258.28

Prepared following general procedure 1 using 4-nitrobenzaldehyde oxime (244 mg, 1.47 mmol) and norbornene (169 mg, 1.80 mmol). Crystallization from methanol gave beige crystals (38 %). mp: 162 °C; ^1^H-NMR (400 MHz, CDCl_3_) δ (ppm) = 8.28 – 8.23 (m, 2H, 3'-H, 5'-H), 7.90 – 7.85 (m, 2H, 2'-H, 6'-H), 4.74 (dt, *J_1_* = 8.3 Hz, *J_2_* = 1.3 Hz, 1H, 7a-H), 3.50 (d, *J* = 8.3 Hz, 1H, 3a-H), 2.70 – 2.65 (m, 1H, 7-H), 2.53 – 2.48 (m, 1H, 4-H), 1.69 – 1.59 (m, 2H, 5-H, 6-H), 1.50 (br. d, *J* = 10.7 Hz, 1H, 8‑H), 1.43 – 1.36 (m, 1H, 5-H), 1.25 (br. d, *J* = 10.7 Hz, 1H, 8-H), 1.23 – 1.17 (m, 1H, 6-H). ^13^C-NMR (126 MHz, CDCl_3_) δ (ppm) = 155.6 (C-3), 148.2 (C-4'), 135.6 (C-1'), 127.4 (C-2', C-6'), 124.0 (C-3', C-5'), 89.1 (C-7a), 56.3 (C-3a), 42.9 (C-7), 39.2 (C-4), 32.4 (C-8), 27.4 (C-5), 22.6 (C-6). IR (KBr) *m/z* (%) = 258 (75) [M^•^]^+^, 219 (48), 191 (40), 181 (78), 169 (86), 131 (100). MS (EI) *m/z* (%) = 258 (75) [M^•^]^+^, 219 (48), 191 (40), 181 (78), 169 (86), 131 (100). HRMS (EI) 258.1007 (calculated for C_14_H_14_N_2_O_3_: 258.1005). Purity (HPLC) > 99 % (λ = 210 nm), > 99 % (λ = 254 nm).

**(3a*RS*,4*RS*,7*SR*,7a*RS*)-3-(3,4,5-Trimethoxyphenyl)-3a,4,5,6,7,7a-hexahydro-4,7-methanobenzo[*d*]isoxazole (EVP-36)**

C_17_H_21_NO_4_, M_r_ = 303.36

Prepared following general procedure 1 using 3,4,5-trimethoxybenzaldehyde oxime (274 mg, 1.30 mmol) and norbornene (125 mg, 1.32 mmol). FCC using hexanes/ethyl acetate 1:1 gave a pale yellow solid (24 %). mp: 145 °C; ^1^H-NMR (400 MHz, CDCl_3_) δ (ppm) = 6.93 (s, 2H, 2'-H, 6'-H), 4.63 (d, *J* = 8.5 Hz, 1H, 7a-H), 3.88 (s, 6H, 3'-O-CH_3_, 5'-O-CH_3_), 3.87 (s, 3H, 4'-O-CH_3_), 3.45 (d, *J* = 8.5 Hz, 1H, 3a-H), 2.65 – 2.58 (m, 1H, 7‑H), 2.55 – 2.49 (m, 1H, 4-H), 1.66 – 1.53 (m, 2H, 5-H, 6-H), 1.52 (br. d, *J*= 10.7 Hz, 1H, 8-H), 1.41 – 1.29 (m, 1H, 5-H), 1.25 – 1.11 (m, 2H, 6-H, 8‑H). ^13^C-NMR (126 MHz, CDCl_3_) δ (ppm) = 156.7 (C-3), 153.3 (C-3', C-5'), 139.7 (C-4'), 124.9 (C-1'), 104.3 (C-2', C-6'), 88.1 (C-7a), 60.9 (4'-O-CH_3_), 57.1 (C-3a), 56.4 (3'-O-CH_3_, 5'-O-CH_3_) 42.9 (C-7), 39.5 (C‑4), 32.4 (C-8), 27.4 (C-5), 22.7 (C-6). IR (KBr)
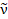
 [cm^-1^] = 3010, 2981, 2952, 2928, 2876, 1597, 1571, 1508, 1455, 1416, 1371, 1238, 1130, 1001, 901, 841, 732. MS (EI) *m/z* (%) = 303 (100) [M^•^]^+^, 288 (40). HRMS (EI) 303.1452 (calculated for C_17_H_21_NO_4_: 303.1471). Purity (HPLC) 98 % (λ = 210 nm), > 99 % (λ = 254 nm).

**(3a*RS*,4*RS*,7*SR*,7a*RS*)-3-Mesityl-4,5,6,7-tetrahydro-4,7-methanobenzo[*d*]isoxazol-7a(3a*H*)-ol (EVP-45)**

C_17_H_21_NO_2_, M_r_ = 271.36

Prepared following general procedure 4 using norcamphor (824 mg, 7.48 mmol) and mesitonitrile oxide (607 mg. 3.77 mmol). Washing the residue with hexanes gave colorless crystals (85 %). mp: 187 °C; ^1^H-NMR (400 MHz, CDCl_3_) δ (ppm) = 6.89 (s, 2H, 3'-H, 5'-H), 2.99 (s, 1H, OH), 2.89 (d, *J* = 2.1 Hz, 1H, 3a-H), 2.66 – 2.60 (m, 1H, 7-H), 2.36 – 2.32 (m, 1H, 4-H), 2.29 (s, 3H, 4'-CH_3_), 2.26 (s, 6H, 2'‑CH_3_, 6'-CH_3_), 1.90 (br. d, *J* = 10.5 Hz, 1H, 8-H), 1.87 – 1.80 (m, 1H, 6-H), 1.70 – 1.62 (m, 1H, 5-H), 1.63 – 1.50 (m, 1H, 6-H), 1.44 – 1.36 (m, 1H, 5-H), 1.34 (br. d, *J* = 10.5 Hz, 1H, 8-H). ^13^C-NMR (126 MHz, CDCl_3_) δ (ppm) = 159.5 (C-3), 138.7 (C-4'), 136.7 (C-2', C-6'), 128.6 (C-3', C-5'), 126.2 (C-1'), 115.4 (C-7a), 65.5 (C-3a), 46.7 (C-7), 40.3 (C-4), 35.6 (C-8), 28.2 (C-5), 22.2 (C-6), 21.1 (4'-CH_3_), 19.99 (2'-CH_3_, 6'-CH_3_). IR (KBr)
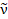
 [cm^-1^] = 3299, 3093, 2960, 2952, 2879, 1613, 1477, 1458, 1333, 1315, 1296, 1230, 1184, 1135, 1075, 1060, 1003, 953, 904, 883, 850, 828, 788. MS (EI) *m/z* (%) = 271 (21) [M^•^]^+^, 270 (85), 219 (57), 181 (60), 169 (63), 131 (100), 130 (95). HRMS (EI) 271.1581 (calculated for C_17_H_21_NO_2_: 271.1572). Purity (HPLC) 99 % (λ = 210 nm), > 99 % (λ = 254 nm).

**(3a*RS*,4*RS*,7*SR*,7a*RS*)-3-(3,5-Dichloropyridin-4-yl)-3a,45,6,7,7a-hexahydro-4,7-methanobenzo[*d*]isoxazole (EVP-46)**

C_13_H_12_Cl_2_N_2_O, M_r_ = 283.15

Prepared following general procedure 1 using 3,5-dichloroisonicotinaldehyde oxime (240 mg, 1.26 mmol) and norbornene (159 mg, 1.69 mmol). FCC using hexanes/ethyl acetate 4:1 gave colorless oil (49 %); ^1^H-NMR (500 MHz, CDCl_3_) δ (ppm) = 8.54 (s, 1H, 2'-H, 6'-H), 4.74 (d, *J* = 8.4 Hz, 1H, 7a-H), 3.54 (d, *J* = 8.4 Hz, 1H, 3a-H), 2.67 (d, *J* = 4.8 Hz, 1H, 7-H), 2.25 (d, *J* = 4.0 Hz, 1H, 4-H), 1.87 (br. d, *J* = 10.7 Hz, 1H, 8-H), 1.63 – 1.54 (m, 1H, 6-H), 1.54 – 1.45 (m, 1H, 5-H), 1.29 (br. d, *J* = 10.7 Hz, 1H, 8-H), 1.20 – 1.13 (m, 2H, 5-H, 6-H). ^13^C-NMR (101 MHz, CDCl_3_) δ (ppm) = 152.4 (C-3), 147.8 (C-2', C-6'), 136.9 (C-4'), 132.0 (C-3', C-5'), 88.5 (C-7a), 59.2 (C-3a), 42.6 (C-7), 39.0 (C-4), 32.9 (C-8), 27.2 (C-5), 22.8 (C-6). IR (Film)
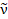
 [cm^-1^] = 3048, 2962, 2876, 1600, 1522, 1453, 1399, 1390, 1326, 1315, 1305, 1222, 1215, 1201, 1107, 921, 907, 887, 824, 814, 737, 701. MS (EI) *m/z* (%) = 286 (15) [(^37^Cl_2_) M^•^]^+^, 284 (47) [(^35^Cl, ^37^Cl) M^•^]^+^, 282 (72) [(^35^Cl_2_) M^•^]^+^, 252 (40), 217 (72), 215 (100). HRMS (EI) 282.0323 (calculated for C_13_H_12_^35^Cl_2_N_2_O: 282.0327). Purity (HPLC) 99 % (λ = 210 nm), > 99 % (λ = 254 nm).

**(3a*RS*,4*RS*,7*SR*,7a*RS*)-3-(Thiophen-2-yl)-3a,4,5,6,7,7a-hexahydro-4,7-methanobenzo[*d*]isoxazole (EVP-51)**

C_12_H_13_NOS, M_r_ = 219.30

Prepared following general procedure 1 using thiophene-2-carbaldehyde oxime (251 mg, 1.97 mmol) and norbornene (230 mg, 2.44 mmol). FCC using hexanes/ethyl acetate 4:1 gave a beige solid (8 %). mp: 74 °C; ^1^H-NMR (500 MHz, CDCl_3_) δ (ppm) = 7.35 (dd, *J_1_* = 5.1 Hz, *J_2_* = 1.1 Hz, 1H, 5'-H), 7.26 (dd, *J_1_* = 3.7 Hz, *J_2_* = 1.1 Hz, 1H, 3'-H), 7.06 (dd, *J_1_* = 5.1 Hz, *J_2_* = 3.7 Hz, 1H, 4'-H), 4.63 (d, *J* = 8.3 Hz, 1H, 7a-H), 3.45 (d, *J* = 8.3 Hz, 1H, 3a-H), 2.66 – 2.59 (m, 2H, 4-H, 7-H), 1.60 – 1.57 (m, 2H, 5-H, 6-H), 1.56 – 1.53 (m, 1H, 8-H), 1.37 – 1.31 (m, 1H, 5-H), 1.21 (br. d, *J* = 10.6 Hz, 1H, 8‑H), 1.19 – 1.12 (m, 1H, 6-H). ^13^C-NMR (101 MHz, CDCl_3_) δ (ppm) = 152.9 (C-3), 132.3 (C-2'), 127.8 (C-5), 127.6 (C-3'), 127.2 (C-4'), 88.2 (C-7a), 58.1 (C-3a), 42.9 (C-7), 39.5 (C-4), 32.4 (C-8), 27.3 (C-5), 22.6 (C-6). IR (Film)
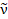
 [cm^-1^] = 3104, 3076, 2964, 2874, 1449, 1438, 1357, 1336, 1314, 1230, 966, 921, 908, 873, 841, 707. MS (EI) *m/z* (%) = 219 (100) [M^•^]^+^. HRMS (EI) 219.0719 (calculated for C_12_H_13_NOS: 219.0718). Purity (HPLC) 98 % (λ = 210 nm), 97 % (λ = 254 nm).

**(3a*RS*,6a*RS*)-3-Mesityl-3a,4,5,6-tetrahydro-6a*H*-cyclopenta[*d*]isoxazol-6a-ol (EVP-52)**

C_15_H_19_NO_2_, M_r_ = 245.32

Prepared following general procedure 4 using cyclopentanone (0.30 mL, 3.4 mmol) and mesitonitrile oxide (245 mg, 1.52 mmol). FCC using a gradient of hexanes/ethyl acetate 4:1 → 1:1 gave bright yellow crystals (78 %). mp: 117 °C; ^1^H-NMR (400 MHz, CDCl_3_) δ (ppm) = 6.90 (s, 2H, 3'-H, 5'-H), 3.63 – 3.56 (m, 1H, 3a-H), 3.15 – 3.06 (m, 1H, OH), 2.39 – 2.32 (m, 1H, 6-H), 2.29 (s, 3H, 4'-CH_3_), 2.28 (s, 6H, 2'-CH_3_, 6'-CH_3_), 2.07 – 1.98 (m, 1H, 6-H), 1.98 – 1.87 (m, 1H, 4-H), 1.86 – 1.81 (m, 1H, 5-H), 1.81 – 1.70 (m, 2H, 5-H, 4-H). ^13^C-NMR (126 MHz, CDCl_3_) δ (ppm) = 160.8 (C-3), 138.8 (C-4'), 136.9 (C-2', C-6'), 128.7 (C-3', C-5'), 125.9 (C-1'), 118.3 (C-6a), 60.4 (C-3a), 39.3 (C-6), 29.1 (C-4), 25.2 (C-5), 21.1 (4'-CH_3_), 19.9 (2'-CH_3_, 6'-CH_3_). IR (Film)
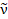
 [cm^-1^] = 3363, 2960, 2871, 1612, 1452, 1378, 1323, 1243, 1222, 1196, 1093, 1072, 872, 852, 812, 787, 755. MS (EI) *m/z* (%) = 245 (35) [M^•^]^+^, 227 (100), 226 (65), 172 (77), 171 (96), 145 (60), 130 (70). HRMS (EI) 245.1398 (calculated for C_15_H_19_NO_2_: 245.1416). Purity (HPLC) 98 % (λ = 210 nm), 97 % (λ = 254 nm).

**(3a*RS*,8a*RS*)-3-Mesityl-3a,4,5,6,7,8-hexahydro-8a*H*-cyclohepta[*d*]isoxazol-8a-ol (EVP-53)**

C_17_H_23_NO_2_, M_r_ = 273.38

Prepared following general procedure 4 using cycloheptanone (226 mg, 2.02 mmol) and mesitonitrile oxide (294 mg, 1.82 mmol). Washing the residue with hexanes gave a colorless solid (36 %). mp: 167 °C; ^1^H-NMR (400 MHz, DMSO-D_6_) δ (ppm) = 6.90 (s, 2H, 3'-H, 5'-H), 6.32 (s, 1H, OH), 3.11 – 3.03 (m, 1H, 3a-H), 2.23 (s, 3H, 4'-CH_3_), 2.19 (s, 6H, 2'-CH_3_, 6'-CH_3_), 2.13 (dd, *J_1_* = 14.2 Hz, *J_2_* = 8.3 Hz, 1H, 8-H), 1.86 (dd, *J_1_* = 14.2 Hz, *J_2_* = 10.9 Hz, 1H, 8-H), 1.80 – 1.65 (m, 3H, 5-H, 6-H, 7-H), 1.54 – 1.40 (m, 1H, 7-H), 1.40 – 1.33 (m, 2H, 4-H, 6-H), 1.33 – 1.16 (m, 2H, 4-H, 5-H). ^13^C-NMR (101 MHz, DMSO-D_6_) δ (ppm) = 160.7 (C-3), 137.9 (C-4'), 136.8 (C-2', C-6'), 128.3 (C-3', C-5'), 126.1 (C-1'), 110.2 (C-8a), 62.0 (C-3a), 36.3 (C-8), 30.6 (C-6), 29.2 (C-5), 26.6 (C-4), 23.7 (C-7), 20.6 (4'-CH_3_), 19.6 (2'-CH_3_, 6'-CH_3_). IR (KBr)
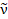
 [cm^-1^] = 3441, 2941, 2927, 2860, 1612, 1444, 1414, 1327, 1276, 1213, 1078, 1052, 1019, 886, 854, 814. MS (EI) *m/z* (%) = 273 (8) [M^•^]^+^, 255 (100), 212 (45), 172 (78), 159 (54), 158 (54). HRMS (EI) 273.1716 (calculated for C_17_H_23_NO_2_: 273.1729). Purity (HPLC) > 99 % (λ = 210 nm), 99 % (λ = 254 nm).

**(3a*RS*,6a*RS*)-3-Mesityl-3a,5,6,6a-tetrahydro-4*H*-cyclopenta[*d*]isoxazole (EVP-55)**

C_15_H_19_NO, M_r_ = 229.32

Prepared following general procedure 2 using mesitaldehyde oxime (307 mg, 1.88 mmol) and cyclopentene (325 mg, 4.77 mmol). FCC using hexanes/ethyl acetate 4:1 gave a bright yellow oil (61 %); ^1^H-NMR (500 MHz, CDCl_3_) δ (ppm) = 6.90 (s, 2H, 3'-H, 5'-H), 5.21 (dd, *J_1_* = 8.8 Hz, *J_2_* = 4.4 Hz, 1H, 6a-H), 3.97 – 3.90 (m, 1H, 3a-H), 2.29 (s, 3H, 4'-CH_3_), 2.27 (s, 6H, 2'-CH_3_, 6'-CH_3_), 2.26 – 2.22 (m, 1H, 6-H), 1.81 – 1.72 (m, 3H, 4-H, 5-H, 6-H), 1.72 – 1.64 (m, 1H, 5-H), 1.64 – 1.56 (m, 1H, 4-H). ^13^C-NMR (126 MHz, CDCl_3_) δ (ppm) = 158.5 (C-3), 138.5 (C-4'), 136.9 (C-2', C-6'), 128.7 (C-3', C-5'), 126.2 (C-1'), 86.3 (C-6a), 56.3 (C-3a), 36.0 (C-6), 30.3 (C-4), 23.6 (C-5), 21.1 (4'-CH_3_), 20.1 (2'-CH_3_, 6'-CH_3_). IR (Film)
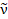
 [cm^-1^] = 2956, 2868, 2733, 1738, 1612, 1571, 1451, 1434, 1332, 1315, 1197, 1083, 1042, 955, 912, 896, 874, 864, 852, 832, 736. MS (EI) *m/z* (%) = 229 (63) [M^•^]^+^, 172 (100). HRMS (EI) 229.1457 (calculated for C_15_H_19_NO: 229.1467). Purity (HPLC) 98 % (λ = 210 nm), 95 % (λ = 254 nm).

**3-Mesityl-5,6,7,8-tetrahydro-4*H*-cyclohepta[*d*]isoxazole (EVP-56)**

C_17_H_21_NO, M_r_ = 255.36

Prepared following general procedure 5 using (3a*RS*,8a*RS*)-3-mesityl-3a,4,5,6,7,8-hexahydro-8a*H*-cyclohepta[*d*]isoxazol-8a-ol **(EVP-53)** (108 mg, 0.396 mmol) to give a colorless oil (92 %); ^1^H-NMR (400 MHz, CDCl_3_) δ (ppm) = 6.95 – 6.88 (m, 2H, 3'-H, 5'-H), 2.98 – 2.90 (m, 2H, 8‑H), 2.31 (s, 3H, 4'‑CH_3_), 2.17 – 2.09 (m, 2H, 4-H), 2.06 (s, 6H, 2'‑CH_3_, 6'‑CH_3_), 1.88 – 1.80 (m, 2H, 6-H), 1.80 – 1.73 (m, 2H, 7-H), 1.65 – 1.58 (m, 2H, 5-H). ^13^C-NMR (101 MHz, CDCl_3_) δ (ppm) = 170.9 (C-8a), 162.6 (C-3), 138.5 (C-4'), 137.4 (C-2', C-6'), 128.1 (C-3', C‑5'), 125.8 (C-1'), 115.9 (C-3a), 30.9 (C-6), 28.3 (C-5), 28.0 (C-8), 26.0 (C-7), 22.7 (C-4), 21.2 (4'‑CH_3_), 19.9 (2'‑CH_3_, 6'‑CH_3_). IR (Film)
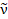
 [cm^-1^] = 3003, 2924, 2851, 1627, 1613, 1445, 1426, 1339, 1173, 1134, 980, 902, 890, 848. MS (EI) *m/z* (%) = 255 (100) [M^•^]^+^, 212 (40), 172 (48). HRMS (EI) 255.1623 (calculated for C_17_H_21_NO: 255.1623). Purity (HPLC) 99 % (λ = 210 nm), 99 % (λ = 254 nm).

**3-Mesityl-5,6-dihydro-4*H*-cyclopenta[*d*]isoxazole (EVP-57)**

C_15_H_17_NO, M_r_ = 227.31

Methyl iodide (3 mL, 48 mmol) was added to a stirred solution of (3a*RS*,6a*RS*)-3-mesityl-6a-morpholino-3a,5,6,6a-tetrahydro-4*H*-cyclopenta[*d*]isoxazole **(EVP-82)** (0.429 g, 1.36 mmol) in acetone (10 mL). The mixture was refluxed for 3 days, evaporated to dryness and the resulting powder was grounded with Ag_2_O (1.0 g, 4.3 mmol). Sublimation at 0.1 mbar and 220 °C gave brown oil that could be purified by FCC using hexanes/ethyl acetate 9:1 to give a yellow oil (62 %); ^1^H-NMR (500 MHz, CDCl_3_) δ (ppm) = 6.92 (s, 2H, 3'-H, 5'-H), 2.86 (tt, *J_1_* = 8.1 Hz, *J_2_* = 1.4 Hz, 2H, 4‑H), 2.69 (tt, *J_1_* = 8.1 Hz, *J_2_* = 6.9 Hz, 2H, 5-H), 2.45 (tt, *J_1_* = 6.9 Hz, *J_2_* = 1.4 Hz, 2H, 6‑H), 2.31 (s, 3H, 4'-CH_3_), 2.16 (s, 6H, 2'-CH_3_, 6'-CH_3_). ^13^C-NMR (126 MHz, CDCl_3_) δ (ppm) = 179.2 (C-6a), 158.5 (C-3), 138.5 (C-4'), 137.2 (C-2', C-6'), 128.2 (C-3', C-5'), 125.7 (C-1'), 124.0 (C-3a), 30.4 (C-5), 24.6 (C-4), 21.6 (C-6), 21.1 (4'-CH_3_), 20.1 (2'‑CH_3_, 6'-CH_3_). IR (Film)
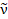
 [cm^-1^] = 3277, 2952, 2923, 2866, 1619, 1575, 1457, 1433, 1377, 1321, 1304, 1218, 1079, 1034, 993, 851, 832. MS (EI) *m/z* (%) = 227 (100) [M^•^]^+^, 226 (63), 171 (60). HRMS (EI) 227.1311 (calculated for C_15_H_17_NO: 227.1310). Purity (HPLC) 99 % (λ = 210 nm), 99 % (λ = 254 nm).

**(3a*RS*,7a*RS*)-5-Ethyl-3-mesityl-4,5,6,7-tetrahydroisoxazolo[4,5-*c*]pyridin-7a(3a*H*)-ol (EVP-59)**

C_17_H_24_N_2_O_2_, M_r_ = 288.39

Prepared following general procedure 4 using 1-ethyl-4-piperidone (501 mg, 3.94 mmol) and mesitonitrile oxide (147 mg, 0.912 mmol). FCC using dichloromethane/methanol 9:1 gave yellow crystals (80 %). mp: 119 °C; ^1^H-NMR (500 MHz, CDCl_3_) δ (ppm) = 6.90 (s, 2H, 3'-H, 5'-H), 3.37 (dd, *J_1_* = 11.0 Hz, *J_2_* = 7.0 Hz, 1H, 3a-H), 2.96 (ddd, *J_1_* = 11.1 Hz, *J_2_* = 7.0 Hz, *J_3_* = 2.2 Hz, 1H, 4-H), 2.90 – 2.86 (m, 1H, 6-H), 2.49 – 2.35 (m, 2H, N-CH_2_), 2.36 – 2.33 (m, 1H, 7-H), 2.33 (s, 6H, 2'-CH_3_, 6'-CH_3_), 2.29 (s, 3H, 4'-CH_3_), 2.22 – 2.14 (m, 2H, 6-H, 7-H), 1.88 (dd, *J_1_* = 11.1 Hz, *J_2_* = 11.0 Hz, 1H, 4‑H), 1.04 (t, *J* = 7.2 Hz, 3H, N-CH_2_-**CH_3_**). ^13^C-NMR (126 MHz, CDCl_3_) δ (ppm) = 163.3 (C-3), 139.0 (C-4'), 137.4 (C-2', C-6'), 128.9 (C-3', C-5'), 125.4 (C‑1'), 104.3 (C-7a), 54.7 (C-3a), 53.5 (C-4), 51.3 (N-CH_2_), 50.0 (C-6), 32.2 (C-7), 21.1 (4'-CH_3_), 20.3 (2'-CH_3_, 6'-CH_3_), 12.3 (N-CH_2_-**CH_3_**). IR (Film)
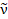
 [cm^-1^] = 3322, 2970, 2927, 2821, 1612, 1473, 1454, 1379, 1364, 1343, 1324, 1302, 1214, 1115, 1082, 1052, 849, 835, 769, 756. MS (EI) *m/z* (%) = 288 (55) [M^•^]^+^, 271 (58), 172 (100), 126 (60). HRMS (EI) 288.1842 (calculated for C_17_H_24_N_2_O_2_: 288.1838). Purity (HPLC) > 99 % (λ = 210 nm), > 99 % (λ = 254 nm).

**(*RS*)-3-Mesityl-5-phenyl-4,5-dihydroisoxazole (EVP-60)**

C_18_H_19_NO, M_r_ = 265.36

This compound has been prepared in a similar manner before by Nitta et al. (1983; 1984) and Lee et al. (2004), but without presenting analytical data.

Prepared following general procedure 2 using mesitaldehyde oxime (288 mg, 1.77 mmol) and styrene (214 mg, 2.06 mmol). FCC using dichloromethane/methanol 98:2 gave a colorless oil (59 %); ^1^H-NMR (500 MHz, CDCl_3_) δ (ppm) = 7.46 – 7.43 (m, 2H, 2''-H, 6''-H), 7.43 – 7.38 (m, 2H, 3''-H, 5''-H), 7.36 – 7.30 (m, 1H, 4''-H), 6.89 (s, 2H, 3'-H, 5'-H), 5.77 (dd, *J_1_* = 10.9 Hz, *J_2_* = 8.1 Hz, 1H, 5-H), 3.60 (dd, *J_1_* = 17.3 Hz, *J_2_* = 10.9 Hz, 1H, 4-H), 3.13 (dd, *J_1_* = 17.3 Hz, *J_2_* = 8.1 Hz, 1H, 4-H), 2.29 (s, 3H, 4'-CH_3_), 2.23 (s, 6H, 2'-CH_3_, 6'-CH_3_). ^13^C-NMR
(126 MHz, CDCl_3_) δ (ppm) = 156.9 (C-3), 141.1 (C-1''), 138.8 (C-4'), 136.6 (C-2', C-6'), 128.7 (C-3'', C-5''), 128.5 (C-3', C-5'), 128.1 (C-4''), 126.0 (C-1'), 125.7 (C-2'', C-6''), 81.8 (C-5), 47.3 (C-4), 21.1 (4'-CH_3_), 19.7 (2'-CH_3_, 6'-CH_3_). IR (Film)
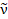
 [cm^-1^] = 3062, 3030, 2951, 2920, 2860, 1611, 1493, 1450, 1325, 876, 849, 758, 731, 699. MS (EI) *m/z* (%) = 265 (100) [M^•^]^+^, 161 (85). HRMS (EI) 265.1470 (calculated for C_18_H_19_NO: 265.1467). Purity (HPLC) 98 % (λ = 210 nm), 97 % (λ = 254 nm).

**(3a*RS*,4*RS*,7*SR*,7a*RS*)-3-(2,6-Dimethylphenyl)-3a,4,5,6,7,7a-hexahydro-4,7-methanobenzo[*d*]isoxazole (EVP-61)**

C_16_H_19_NO, M_r_ = 241.33

Prepared following general procedure 1 using 2,6-dimethylbenzaldehyde oxime (279 mg, 1.88 mmol) and norbornene (249 mg, 2.64 mmol). FCC using hexanes/ethyl acetate 9:1 gave colorless crystals (71 %). mp: 96 °C; ^1^H-NMR (500 MHz, CDCl_3_) δ (ppm) = 7.17 (t, *J* = 7.6 Hz, 1H, 4'-H), 7.07 (d, *J* = 7.6 Hz, 2H, 3'-H, 5'-H), 4.65 (d, *J* = 8.3 Hz, 1H, 7a-H), 3.28 (d, *J* = 8.4 Hz, 1H, 3a-H), 2.68 (d, *J* = 4.8 Hz, 1H, 7-H), 2.31 (s, 6H, 2'-CH_3_, 6'-CH_3_), 2.28 (d, *J* = 4.3 Hz, 1H, 4-H), 1.83 (br. d, *J* = 10.5 Hz, 1H, 8-H), 1.64 – 1.54 (m, 1H, 6-H), 1.54 – 1.44 (m, 1H, 5-H), 1.30 (br. d, *J* = 10.5 Hz, 1H, 8-H), 1.20 – 1.10 (m, 2H, 5-H, 6-H). ^13^C-NMR (126 MHz, CDCl_3_) δ (ppm) = 157.8 (C-3), 136.9 (C-2', C-6'), 129.5 (C-1'), 128.7 (C-4'), 127.9 (C-3', C-5'), 86.9 (C-7a), 61.5 (C-3a), 42.7 (C-7), 38.9 (C-4), 32.8 (C-8), 27.6 (C-5), 22.8 (C-6), 20.3 (2'-CH_3_, 6'-CH_3_). IR (KBr)
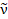
 [cm^-1^] = 3065, 3023, 2960, 2929, 2872, 1467, 1456, 1326, 1314, 1301, 917, 901, 871, 855, 819, 779, 728. MS (EI) *m/z* (%) = 241 (100) [M^•^]^+^, 212 (50), 198 (80), 171 (60), 145 (64), 144 (85). HRMS (EI) 241.1487 (calculated for C_16_H_19_NO: 241.1467). Purity (HPLC) 96 % (λ = 210 nm), 98 % (λ = 254 nm).

**(4*RS*,5*RS*)-5-Ethyl-3-mesityl-4-methyl-4,5-dihydroisoxazol-5-ol (EVP-62)**

C_15_H_21_NO_2_, M_r_ = 247.34

Prepared following general procedure 4 using 3-pentanone (0.42 mL, 4.0 mmol) and mesitonitrile oxide (0.21 g, 1.3 mmol). FCC using hexanes/ethyl acetate 4:1 gave a colorless solid (87 %). mp: 95 °C; ^1^H-NMR (400 MHz, DMSO-D_6_) δ (ppm) = 6.91 (s, 2H, 3'-H, 5'-H), 6.28 (s, 1H, OH), 3.37 (q, *J* = 7.4 Hz, 1H, 4-H), 2.24 (s, 3H, 4'-CH_3_), 2.15 (s, 6H, 2'-CH_3_, 6'-CH_3_), 1.88 (q, *J* = 7.5 Hz, 2H, 5-CH_2_), 0.98 (t, *J* = 7.5 Hz, 3H, 5-CH_2_-**CH_3_**), 0.83 (d, *J* = 7.5 Hz, 3H, 4-CH_3_). ^13^C-NMR (101 MHz, DMSO-D_6_) δ (ppm) = 161.8 (C-3), 137.8 (C-4'), 136.8 (C-2', C-6'), 128.1 (C-3', C-5'), 126.4 (C‑1'), 107.8 (C-5), 49.4 (C-4), 30.8 (5-CH_2_), 20.6 (4'-CH_3_), 19.6 (2'-CH_3_, 6'-CH_3_), 9.0 (4-CH_3_), 8.5 (5-CH_2_-**CH_3_**). IR (KBr)
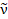
 [cm^-1^] = 3425, 2995, 2979, 2966, 2942, 2921, 2883, 2858, 1613, 1463, 1189, 992, 966, 916, 854, 840, 815. MS (EI) *m/z* (%) = 247 (10) [M^•^]^+^, 229 (45), 200 (65), 172 (100). HRMS (EI) 247.1572 (calculated for C_15_H_21_NO_2_: 247.1572). Purity (HPLC) > 99 % (λ = 210 nm), > 99 % (λ = 254 nm).

**((4*RS*,5*RS*)-3-Mesityl-4,5-dihydroisoxazole-4,5-diyl)dimethanol (EVP-63)**

C_14_H_19_NO_3_, M_r_ = 249.31

Prepared following general procedure 2 using mesitaldehyde oxime (0.20 g, 1.2 mmol) and *cis*-but-2-ene-1,4-diol (0.16 g, 1.8 mmol). FCC using a gradient hexanes/ethyl acetate 4:1 → dichloromethane/methanol 95:5 to give colorless crystals (18 %). mp: 103°C; ^1^H-NMR (500 MHz, CDCl_3_) δ (ppm) = 6.89 (s, 2H, 3'-H, 5'-H), 4.84 (ddd, *J_1_* = 10.0 Hz, *J_2_* = 6.4 Hz, *J_3_* = 3.8 Hz, 1H, 5-H), 4.07 (dd, *J_1_* = 12.2 Hz, *J_2_* = 6.4 Hz, 1H, 5-CH_2_), 3.98 (dd, *J_1_* = 12.2 Hz, *J_2_* = 3.8 Hz, 1H, 5-CH_2_), 3.88 – 3.80 (m, 2H, 4-H, 4-CH_2_), 3.62 – 3.54 (m, 1H, 4-CH_2_), 3.26 (s, 2H, 4‑CH_2_‑**OH**, 5-CH_2_‑**OH**), 2.28 (s, 3H, 4'‑CH_3_), 2.24 (s, 6H, 2'-CH_3_, 6'-CH_3_). ^13^C-NMR (101 MHz, CDCl_3_) δ (ppm) = 158.9 (C-3), 139.1 (C-4'), 136.9 (C-2', C-6'), 128.9 (C-3', C-5'), 124.9 (C-1'), 81.7 (C-5), 59.9 (5-CH_2_), 57.9 (4-CH_2_), 55.5 (C-4), 21.1 (4'-CH_3_), 20.1 (2'-CH_3_, 6'-CH_3_). IR (Film)
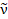
 [cm^-1^] = 3384, 2922, 1611, 1454, 1378, 1327, 1052, 852. MS (EI) *m/z* (%) = 249 (39) [M^•^]^+^, 188 (65), 169 (67), 158 (63), 145 (77), 130 (100). HRMS (EI) 249.1366 (calculated for C_14_H_19_NO_3_: 249.1365).

This compound has been prepared in a different manner and described by Lee et al. (2006) before, but with deviating NMR data. The published NMR data of the claimed *trans*-isomer is equal to the above mentioned. The *cis*-configuration of the structure was confirmed by x-ray analysis.

**(3a*RS*,4*RS*,7*SR*,7a*RS*)-3-(5-Chlorofuran-2-yl)-3a,4,5,6,7,7a-hexahydro-4,7-methanobenzo[*d*]isoxazole (EVP-64)**

C_12_H_12_ClNO_2_, M_r_ = 237.68

*N*‑Chlorosuccinimide (0.37 g, 2.8 mmol) was added to a stirred solution of furan-2-carbaldehyde oxime (0.16 g, 1.4 mmol) in DMF (4 mL). After stirring for 18 hours the mixture was poured in water (30 mL) and extracted with diethyl ether (3 × 30 mL). The combined organic layers were dried over sodium sulfate, filtered and concentrated. Without further purification the residue was dissolved in dichloromethane (8 mL) together with norbornene (0.27 g, 2.9 mmol). A solution of triethylamine (0.5 mL) in dichloromethane (5 mL) was added dropwise. The mixture was stirred for 14 hours at room temperature, poured in water and extracted with diethyl ether (3 × 30 mL). The combined organic layers were dried over sodium sulfate, filtered and concentrated. FCC using hexanes/ethyl acetate 4:1 gave a beige powder (63 %). mp: 124 °C; ^1^H-NMR (400 MHz, CDCl_3_) δ (ppm) = 6.69 (d, *J* = 3.5 Hz, 1H, 3'-H), 6.26 (d, *J* = 3.5 Hz, 1H, 4'-H), 4.60 (d, *J* = 8.3 Hz, 1H, 7a-H), 3.37 (d, *J* = 8.3 Hz, 1H, 3a-H), 2.65 – 2.58 (m, 1H, 7‑H), 2.58 – 2.50 (m, 1H, 4-H), 1.61 – 1.54 (m, 2H, 5-H, 6-H), 1.53 – 1.46 (m, 1H, 8-H), 1.36 – 1.28 (m, 1H, 5-H), 1.21 (br. d, *J* = 10.5 Hz, 1H, 8-H), 1.18 – 1.10 (m, 1H, 6-H). ^13^C-NMR (101 MHz, CDCl_3_) δ (ppm) = 148.9 (C-3), 144.8 (C-2'), 138.5 (C-5'), 113.0 (C-3'), 108.4 (C-4'), 87.9 (C‑7a), 57.1 (C-3a), 42.8 (C-7), 39.4 (C-4), 32.3 (C-8), 27.3 (C-5), 22.7 (C-6). IR (Film)
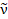
 [cm^-1^] = 3127, 2965, 2876, 1495, 1456, 1326, 1317, 1205, 1152, 1016, 941, 920, 907, 866, 854, 786. MS (EI) *m/z* (%) = 237 (100) [(^35^Cl) M^•^]^+^. HRMS (EI) 237.0558 (calculated for C_12_H_12_^35^ClNO_2_: 237.0557). Purity (HPLC) 99 % (λ = 210 nm), 99 % (λ = 254 nm).

**5-Ethyl-3-mesityl-4,5,6,7-tetrahydroisoxazolo[4,5-*c*]pyridine (EVP-68)**

C_17_H_22_N_2_O, M_r_ = 270.38

Prepared following general procedure 5 using (3a*RS*,7a*RS*)-5-ethyl-3-mesityl-4,5,6,7-tetrahydroisoxazolo[4,5-c]pyridin-7a(3a*H*)-ol **(EVP-59)** (130 mg, 0.450 mmol) to give an orange oil (99 %); ^1^H-NMR (500 MHz, CDCl_3_) δ (ppm) = 6.92 (s, 2H, 3'-H, 5'-H), 3.16 (s, 2H, 4-H), 2.94 – 2.89 (m, 2H, 7‑H), 2.85 (t, *J* = 5.6 Hz, 2H, 6-H), 2.60 (q, *J* = 7.2 Hz, 2H, N-CH_2_), 2.31 (s, 3H, 4'-CH_3_), 2.10 (s, 6H, 2'-CH_3_, 6'-CH_3_), 1.12 (t, *J* = 7.2 Hz, 3H, N-CH_2_-**CH_3_**). ^13^C-NMR (101 MHz, CDCl_3_) δ (ppm) = 166.8 (C-7a), 159.5 (C-3), 138.8 (C-4'), 137.2 (C-2', C-6'), 128.2 (C-3', C‑5'), 125.2 (C-1'), 111.9 (C-3a), 51.1 (N-CH_2_), 49.2 (C-6), 47.3 (C-4), 23.8(C-7), 21.2 (4'-CH_3_), 20.0 (2'-CH_3_, 6'-CH_3_), 12.5 (N-CH_2_-**CH_3_**). IR (Film)
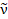
 [cm^-1^] = 2970, 2922, 2804, 2765, 1648, 1613, 1455, 1377, 1330, 1211, 1146, 1090, 852, 755. MS (EI) *m/z* (%) = 270 (95) [M^•^]^+^, 269 (100), 255 (72). HRMS (EI) 270.1732 (calculated for C_17_H_22_N_2_O [M^•^]^+^: 270.1732). Purity (HPLC) 97 % (λ = 210 nm), 97 % (λ = 254 nm).

**(*RS*)-4-(3-Mesityl-4,5-dihydroisoxazol-5-yl)butan-1-ol (EVP-70)**

C_16_H_23_NO_2_, M_r_ = 261.37

Prepared following general procedure 3a using mesitonitrile oxide (161 mg, 0.999 mmol) and hex-5-en-1-ol (450 mg, 4.49 mmol). FCC using dichloromethane/methanol 95:5 gave a colorless solid (85 %). mp: 71 °C; ^1^H-NMR (500 MHz, CDCl_3_) δ (ppm) = 6.89 (s, 2H, 3''-H, 5''-H), 4.78 – 4.67 (m, 1H, 5'-H), 3.68 (t, *J* = 6.3 Hz, 2H, 1‑H), 3.22 (dd, *J_1_* = 17.2 Hz, *J_2_* = 10.2 Hz, 1H, 4'-H), 2.79 (dd, *J_1_* = 17.2 Hz, *J_2_* = 8.2 Hz, 1H, 4'), 2.28 (s, 3H, 4''-CH_3_), 2.24 (s, 6H, 2''-CH_3_, 6''-CH_3_), 1.93 – 1.81 (m, 1H, 4-H), 1.71 – 1.59 (m, 4H, 2-H, 3-H, 4-H), 1.55 – 1.48 (m, 1H, 3‑H). ^13^C-NMR (101 MHz, CDCl_3_) δ (ppm) = 157.3 (C-3'), 138.7 (C-4''), 136.5 (C-2'', C-6''), 128.4 (C-3'', C-5''), 126.6 (C‑1''), 80.5 (C-5'), 62.7 (C-1), 44.1 (C-4'), 35.2 (C-4), 32.5 (C-2), 22.1 (C-3), 21.1 (4''‑CH_3_), 19.72 (2''‑CH_3_, 6''‑CH_3_). IR (KBr)
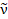
 [cm^-1^] = 3420, 3000, 2946, 2935, 2860, 1606, 1471, 1454, 1439, 1426, 1333, 1186, 1052, 1036, 913, 867, 853, 819. MS (EI) *m/z* (%) = 261 (11) [M^•^]^+^, 188 (90), 159 (100), 158 (74). HRMS (EI) 261.1724 (calculated for C_16_H_23_NO_2_: 261.1729). Purity (HPLC) 98 % (λ = 210 nm), 98 % (λ = 254 nm).

**5-Ethyl-3-mesityl-4-methylisoxazole (EVP-71)**

C_15_H_19_NO, M_r_ = 229.32

Prepared following general procedure 5 using (4*RS*,5*RS*)-5-ethyl-3-mesityl-4-methyl-4,5-dihydroisoxazol-5-ol (**EVP-62**) (0.22 g, 0.89 mmol) to give a yellow oil (93 %); ^1^H-NMR (400 MHz, CDCl_3_) δ (ppm) = 6.92 (s, 2H, 3'-H, 5'-H), 2.78 (q, *J* = 7.6 Hz, 2H, 5-CH_2_), 2.32 (s, 3H, 4'-CH_3_), 2.06 (s, 6H, 2'-CH_3_, 6'-CH_3_), 1.71 (s, 3H, 4-CH_3_), 1.34 (t, *J* = 7.6 Hz, 3H, 5-CH_2_-CH_3_). ^13^C-NMR (101 MHz, CDCl_3_) δ (ppm) = 169.6 (C-5), 163.2 (C-3), 138.5 (C-4'), 137.2 (C-2', C-6'), 128.1 (C-3', C‑5'), 125.9 (C-1'), 108.5 (C-4), 21.2 (4'-CH_3_), 19.8 (2'-CH_3_, 6'-CH_3_), 19.4 (5-CH_2_), 11.9 (5-CH_2_-CH_3_), 6.7 (4-CH_3_). IR (Film)
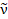
 [cm^-1^] = 2976, 2924, 1630, 1614, 1460, 1427, 1386, 1377, 1325, 1190, 1035, 1011, 894, 850. MS (EI) *m/z* (%) = 229 (76) [M^•^]^+^, 200 (100), 172 (90). HRMS (EI) 229.1468 (calculated for C_15_H_19_NO: 229.1467). Purity (HPLC) 99 % (λ = 210 nm), 99 % (λ = 254 nm).

**(3a*RS*,7a*SR*)-3-Mesityl-3a,4,6,7-tetrahydro-7a*H*-pyrano[3,4-*d*]isoxazol-7a-ol (EVP-73)**

C_15_H_19_NO_3_, M_r_ = 261.32

Prepared following general procedure 4 using tetrahydro-4*H*-pyran-4-one (0.37 g, 3.7 mmol) and mesitonitrile oxide (0.20 g, 1.2 mmol). FCC using hexanes/ethyl acetate 1:1 gave a pale yellow oil (80 %); ^1^H-NMR (500 MHz, CDCl_3_) δ (ppm) = 6.90 (s, 2H, 3'-H, 5'-H), 4.04 – 3.99 (m, 2H, 4-H, 6-H), 3.54 (ddd, *J_1_* = 11.8 Hz, *J_2_* = 11.8 Hz, *J_3_* = 3.5 Hz, 1H, 6-H), 3.34 (dd, *J_1_* = 9.6 Hz, *J_2_* = 6.8 Hz, 1H, 3a-H), 3.24 (dd, *J_1_* = 11.4 Hz, *J_2_* = 9.6 Hz, 1H, 4-H), 2.92 (s, 1H, OH), 2.31 (s, 6H, 2‘-CH_3_, 6‘‑CH_3_), 2.29 (s, 3H, 4'-CH_3_), 2.28 – 2.26 (m, 1H, 7-H), 2.19 (ddd, *J_1_* = 14.2 Hz, *J_2_* = 11.9 Hz, *J_3_* = 6.1 Hz, 1H, 7-H). ^13^C-NMR (101 MHz, CDCl_3_) δ (ppm) = 161.8 (C-3), 139.2 (C-4'), 137.2 (C-2', C-6'), 128.9 (C-3', C-5'), 125.0 (C-1'), 103.5 (C-7a), 66.7 (C-4), 65.0 (C-6), 55.1 (C-3a), 32.0 (C-7), 21.1 (4'-CH_3_), 20.1 (2'‑CH_3_, 6'-CH_3_). IR (Film)
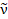
 [cm^-1^] = 3357, 2963, 2923, 2865, 1611, 1452, 1380, 1347, 1322, 1286, 1217, 1123, 1098, 1022, 943, 838. MS (EI) *m/z* (%) = 261 (100) [M^•^]^+^, 244 (58), 172 (95), 145 (60). HRMS (EI) 261.1366 (calculated for C_15_H_19_NO_3_: 261.1365). Purity (HPLC) 98 % (λ = 210 nm), > 99 % (λ = 254 nm).

**2-((4*RS*,5*RS*)-4-Ethyl-3-mesityl-4,5-dihydroisoxazol-5-yl)ethan-1-ol (EVP-76a)**

C_16_H_23_NO_2_, M_r_ = 261.37

Prepared following general procedure 3b using mesitonitrile oxide (329 mg, 2.04 mmol) and *cis*-hex-3-en-1-ol (335 mg, 3.34 mmol). FCC hexanes/diethyl ether 3:7 was used to separate **EVP-76b** (eluting first; colorless crystals, 29 %) from its isomer **EVP-76a** (eluting second; colorless oil, 38 %); ^1^H-NMR (500 MHz, CDCl_3_) δ (ppm) = 6.89 (s, 2H, 3'''-H, 5'''-H), 4.88 (ddd, *J_1_* = 11.5 Hz, *J_2_* = 9.4 Hz, *J_3_* = 2.6 Hz, 1H, 5'-H), 3.97 – 3.89 (m, 2H, 1-H), 3.43 (ddd, *J_1_* = 9.4 Hz, *J_2_* = 9.3 Hz, *J_3_* = 5.8 Hz, 1H, 4'‑H), 2.29 (s, 3H, 4'''-CH_3_), 2.24 (s, 6H, 2'''-CH_3_, 6'''-CH_3_), 2.02 – 1.93 (m, 1H, 2-H), 1.86 – 1.79 (m, 1H, 2-H), 1.61 – 1.51 (m, 1H, 1''-H), 1.44 – 1.32 (m, 1H, 1''-H), 0.82 (t, *J* = 7.4 Hz, 3H, 2''-H). ^13^C-NMR (126 MHz, CDCl_3_) δ (ppm) = 162.1 (C-3'), 138.8 (C-4'''), 136.9 (C-2''', C-6'''), 128.8 (C-3''', C-5'''), 126.3 (C-1'''), 81.3 (C-5'), 60.8 (C-1), 55.5 (C-4'), 31.5 (C-2), 21.2 (4'''-CH_3_), 20.3 (2'''-CH_3_, 6'''-CH_3)_, 18.9 (C-1''), 13.3 (C-2''). IR (Film)
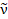
 [cm^-1^] = 3416, 2961, 2934, 2878, 1612, 1458, 1379, 1328, 1305, 1289, 1214, 1047, 889, 852, 832. MS (EI) *m/z* (%) = 261 (27) [M^•^]^+^, 216 (100), 172 (40), 145 (50), 130 (62). HRMS (EI) 261.1727 (calculated for C_16_H_23_NO_2_: 261.1729). Purity (HPLC) 99 % (λ = 210 nm), > 99 % (λ = 254 nm).

**2-((4*RS*,5*RS*)-5-Ethyl-3-mesityl-4,5-dihydroisoxazol-4-yl)ethan-1-ol (EVP-76b)**

C_16_H_23_NO_2_, M_r_ = 261.37

Obtained as the first eluting isomer in the reaction described above (colorless crystals, 29 %). mp: 84 °C; ^1^H-NMR (500 MHz, CDCl_3_) δ (ppm) = 6.88 (s, 2H, 3'''-H, 5'''-H), 4.58 (ddd, *J_1_* = 9.7 Hz, *J_2_* = 9.7 Hz, *J_3_* = 3.7 Hz, 1H, 5'-H), 3.66 (ddd, *J_1_* = 9.4 Hz, *J_2_* = 8.2 Hz, *J_3_* = 6.4 Hz, 1H, 4'-H), 3.48 – 3.33 (m, 2H, 1‑H), 2.28 (s, 3H, 4'''-CH_3_), 2.25 (s, 6H, 2'''-CH_3_, 6'''-CH_3_), 1.86 – 1.76 (m, 1H, 2-H), 1.77 – 1.69 (m, 1H, 1''-H), 1.66 – 1.60 (m, 1H, 1''-H), 1.57 – 1.49 (m, 1H, 2-H), 1.13 (t, *J* = 7.3 Hz, 3H, 2''-H). ^13^C-NMR (126 MHz, CDCl_3_) δ (ppm) = 161.4 (C-3'), 161.4 (C-4'''), 138.7 (C-2''', C-6'''), 128.7 (C3''', C-5'''), 126.3 (C-1'''), 84.4 (C5'), 60.8 (C-1), 49.1 (C-4'), 28.3 (C-2), 22.5 (C-1''), 21.1 (4'''-CH_3_), 20.2 (2'''-CH_3_, 6'''-CH_3_), 11.1 (C-2''). IR (Film)
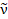
 [cm^-1^] = 3417, 2964, 2941, 2877, 1611, 1455, 1378, 1327, 1309, 1055, 894, 852, 755. MS (EI) *m/z* (%) = 261 (30) [M^•^]^+^, 232 (100), 145 (47), 130 (62). HRMS (EI) 261.1728 (calculated for C_16_H_23_NO_2_: 261.1729). Purity (HPLC) 91 % (λ = 210 nm), 94 % (λ = 254 nm).

**3-Mesityl-6,7-dihydro-4*H*-pyrano[3,4-*d*]isoxazole (EVP-79)**

C_15_H_17_NO_2_, M_r_ = 243.31

Prepared following general procedure 5 using (3a*RS*,7a*SR*)-3-mesityl-3a,4,6,7-tetrahydro-7a*H*-pyrano[3,4-d]isoxazol-7a-ol **(EVP-73)** (0.25 g, 0.96 mmol). FCC using hexanes/ethyl acetate 4:1 gave colorless oil (68 %); ^1^H-NMR (500 MHz, CDCl_3_) δ (ppm) = 6.92 (s, 2H, 3'-H, 5'-H), 4.36 (t, *J* = 1.7 Hz, 2H, 4-H), 4.00 (t, *J* = 5.6 Hz, 2H, 6-H), 2.94 (tt, *J_1_* = 5.5 Hz, *J_2_* = 1.7 Hz, 2H, 7-H), 2.31 (s, 3H, 4'-CH_3_), 2.10 (s, 6H, 2'-CH_3_, 6'-CH_3_). ^13^C-NMR (101 MHz, CDCl_3_) δ (ppm) = 165.4 (C-7a), 158.5 (C-3), 139.0 (C-4'), 137.1 (C-2', C-6'), 128.3 (C-3', C‑5'), 124.9 (C-1'), 111.7 (C-3a), 63.8 (C-6), 62.5 (C-4), 24.6 (C-7), 21.1 (4'-CH_3_), 19.9 (2'-CH_3_, 6'-CH_3_). IR (Film)
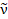
 [cm^-1^] = 2922, 2856, 1638, 1612, 1441, 1327, 1208, 1180, 1081, 986, 958, 854, 836, 743. MS (EI) *m/z* (%) = 243 (100) [M^•^]^+^, 242 (78), 213 (55), 186 (45), 130 (40). HRMS (EI) 243.1257 (calculated for C_15_H_17_NO_2_: 243.1259). Purity (HPLC) 99 % (λ = 210 nm), 99 % (λ = 254 nm).

**(3a*RS*,6a*RS*)-3-Mesityl-6a-morpholino-3a,5,6,6a-tetrahydro-4*H*-cyclopenta[*d*]isoxazole (EVP-82)**

C_19_H_26_N_2_O_2_, M_r_ = 314.43

A solution of mesitonitrile oxide (1.01 g, 6.24 mmol) and 1-morpholinocyclopentene (3.0 mL, 19 mmol) in 20 mL dichloromethane was stirred for 20 hours at room temperature. Then the mixture was poured into water (30 mL) and extracted with dichloromethane (3 × 30 mL). The combined organic layers were washed with brine, dried over magnesium sulfate, filtered and concentrated. FCC using hexanes/ethyl acetate 7:3 containing 5 % triethylamine followed by crystallization from ethanol gave colorless crystals (92 %). mp: 119 °C; ^1^H-NMR
(400 MHz, CDCl_3_) δ (ppm) = 6.89 (s, 2H, 3'-H, 5'-H), 3.81 – 3.72 (m, 4H, 2''-H, 6''-H), 3.68 – 3.63 (m, 1H, 3a-H), 2.92 – 2.81 (m, 4H, 3''-H, 5''-H), 2.43 – 2.36 (m, 1H, 6-H), 2.28 (s, 3H, 4'‑CH_3_), 2.27 (s, 6H, 2'‑CH_3_, 6'-CH_3_), 1.95 – 1.86 (m, 1H, 6-H), 1.86 – 1.81 (m, 1H, 5-H), 1.81 – 1.74 (m, 2H, 4-H, 5-H), 1.73 – 1.68 (m, 1H, 4-H). ^13^C-NMR (126 MHz, CDCl_3_) δ (ppm) = 158.3 (C-3), 138.6 (C-4'), 137.2 (C-2', C-6'), 129.0 (C-3', C-5'), 126.5 (C-1'), 113.1 (C-6a), 67.0 (C-2'', C-6''), 56.0 (C-3a), 47.0 (C-3'', C-5''), 39.3 (C-6), 29.7 (C-4), 24.5 (C-5), 21.0 (4'-CH_3_), 20.8 (2'-CH_3_, 6'-CH_3_). IR (KBr)
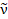
 [cm^-1^] = 2988, 2973, 2951, 2926, 2896, 2856, 1737, 1699, 1612, 1596, 1454, 1343, 1334, 1273, 1227, 1210, 1133, 1117, 1072, 1032, 881, 874, 845, 746. MS (EI) *m/z* (%) = 314 (7) [M^•^]^+^, 153 (100). HRMS (EI) 314.1995 (calculated for C_17_H_24_N_2_O_2_: 314.1994). Purity (HPLC) 97 % (λ = 210 nm), > 99 % (λ = 254 nm).

**5-Butyl-3-mesitylisoxazole (EVP-83)**

C_16_H_21_NO, M_r_ = 243.35

Hexan-2-one (0.30 mL, 2.4 mmol) was added dropwise to a solution of lithium diisopropylamide (4.0 mmol) in 4 mL dry THF, cooled to -78 °C under nitrogen atmosphere. The solution was stirred at -78 °C for 2 hours before mesitonitrile oxide (0.23 g, 1.4 mmol), dissolved in 4 mL dry THF, was added dropwise. The solution was allowed to reach room temperature slowly by stirring for another 14 hours. A saturated aqueous solution of ammonium chloride (30 mL) was added and the mixture was extracted with ethyl acetate (3 × 30 mL). The combined organic layers were washed with brine, dried over sodium sulfate, filtered and concentrated. The crude product was purified by FCC using hexanes/ethyl acetate 4:1. Without further characterization, the resulting 5-butyl-3-mesityl-4,5-dihydroisoxazol-5-ol was dissolved in methanol (5 mL). A solution of sodium carbonate (0.50 g, 4.7 mmol) in 2 mL water was added and the mixture was refluxed for 2 hours. After cooling down to room temperature, water (20 mL) was added and the mixture was extracted with dichloromethane (3 × 20 mL). The combined organic layers were dried over magnesium sulfate, filtered and concentrated. FCC using hexanes/ethyl acetate 9:1 gave a colorless oil (50 %); ^1^H-NMR (400 MHz, CDCl_3_) δ (ppm) = 6.93 (m, 2H, 3''-H, 5''-H), 5.91 (t, *J* = 0.8 Hz, 1H, 4-H), 2.82 (td, *J_1_* = 7.7 Hz, *J_2_* = 0.8 Hz, 2H, 1'-H), 2.32 (s, 3H, 4''-CH_3_), 2.14 (s, 6H, 2''-CH_3_, 6''-CH_3_), 1.80 – 1.68 (m, 2H, 2'-H), 1.50 – 1.38 (m, 2H, 3'-H), 0.97 (t, *J* = 7.4 Hz, 3H, 4'-H). ^13^C-NMR (101 MHz, CDCl_3_) δ (ppm) = 173.6 (C-5), 162.1 (C-3), 138.6 (C-4''), 137.2 (C-2'', C-6''), 128.4 (C-3'', C‑5''), 126.7 (C-1''), 102.3 (C-4), 29.7 (C-2'), 26.6 (C-1'), 22.3 (C-3'), 21.2 (4''-CH_3_), 20.3 (2''-CH_3_, 6''-CH_3_), 13.8 (C-4'). IR (Film)
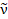
 [cm^-1^] = 3125, 2958, 2930, 2872, 2864, 1613, 1595, 1458, 1425, 1394, 1379, 1180, 1033, 1000, 934, 887, 851, 800. MS (EI) *m/z* (%) = 243 (65) [M^•^]^+^, 186 (100), 159 (62), 158 (66). HRMS (EI) 243.1623 (calculated for C_16_H_21_NO: 243.1623). Purity (HPLC) > 99 % (λ = 210 nm), > 99 % (λ = 254 nm).

**(3a*RS*,4*RS*,7*SR*,7a*RS*)-3-Mesityl-7a-morpholino-3a,4,5,6,7,7a-hexahydro-4,7-methanobenzo[*d*]isoxazole (EVP-85)**

C_21_H_28_N_2_O_2_, M_r_ = 340.47

*p*-Toluenesulfonic acid monohydrate (24 mg, 0.13 mmol) was added to a stirred solution of norcamphor (0.42 g, 3.8 mmol) in *N*-(trimethlsilyl)morpholine (2.0 mL, 11 mmol) at room temperature. After 24 hours of stirring mesitonitrile oxide (0.30 g, 1.9 mmol), dissolved in dichloromethane (2 mL), was added and the mixture stirred for another 2 hours. The solvent was evaporated in vacuo. FCC using hexanes/ethyl acetate 7:3, followed by crystallization from hexanes, containing few drops of dichloromethane gave colorless crystals (95 %). mp: 165 °C; ^1^H-NMR (400 MHz, CDCl_3_) δ (ppm) = 6.88 (s, 2H, 3'-H, 5'-H), 3.83 – 3.65 (m, 4H, 2''-H, 6''-H), 2.90 (s, 1H, 3a-H), 2.87 – 2.74 (m, 5H, 7-H, 3''-H, 5''-H), 2.28 (s, 9H, 2'-CH_3_, 4'-CH_3_, 6'-CH_3_), 2.25 – 2.20 (m, 1H, 4-H), 1.93 (br. d, *J_1_* = 10.4 Hz, 1H, 8-H), 1.76 – 1.66 (m, 1H, 6-H), 1.66 – 1.56 (m, 1H, 5-H), 1.49 – 1.39 (m, 1H, 6-H), 1.35 – 1.30 (m, 1H, 8-H), 1.30 – 1.24 (m, 1H, 5-H). ^13^C-NMR (101 MHz, CDCl_3_) δ (ppm) = 157.7 (C-3), 138.6 (C-4'), 137.1 (C-2', C-6'), 129.1 (C-3', C-5'), 127.0 (C-1'), 111.2 (C-7a), 67.2 (C-2'', C-6''), 61.3 (C-3a), 47.1 (C-3'', C-5''), 44.9 (C-7), 40.1 (C-4), 35.0 (C-8), 27.9 (C-5), 23.0 (C-6), 21.2 (2'-CH_3_, 6'-CH_3_), 21.1 (4'-CH_3_). IR (KBr)
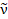
 [cm^-1^] = 3020, 2980, 2949, 2922, 2860, 2848, 1609, 1455, 1333, 1270, 1226, 1119, 887, 879, 851, 809, 770, 732. MS (EI) *m/z* (%) = 340 (20) [M^•^]^+^, 179 (60), 151 (100). HRMS (EI) 340.2151 (calculated for C_21_H_28_N_2_O_2_: 340.2151). Purity (HPLC) > 99 % (λ = 210 nm), 97 % (λ = 254 nm).

**(3a*RS*,7a*RS*)-3-Mesityl-3a,4,6,7-tetrahydro-7a*H*-thiopyrano[3,4-*d*]isoxazol-7a-ol (EVP-86)**

C_15_H_19_NO_2_S, M_r_ = 277.38

Prepared following general procedure 4 using tetrahydro-4*H*-thiopyran-4-one (0.34 g, 2.9 mmol) and mesitonitrile oxide (0.40 g, 2.5 mmol). FCC using hexanes/ethyl acetate 3:1, followed by crystallization from ethanol gave pale yellow crystals (94 %). mp: 171 °C; ^1^H-NMR (500 MHz, DMSO-D_6_) δ (ppm) = 6.92 (s, 2H, 3'-H, 5'-H), 6.73 (s, 1H, OH), 3.26 (dd, *J_1_* = 10.0 Hz, *J_2_* = 5.5 Hz, 1H, 3a-H), 2.75 – 2.65 (m, 2H, 4-H, 6-H), 2.59 (ddd, *J_1_* = 12.9 Hz, *J_2_* = 10.0 Hz, *J_3_* = 3.7 Hz, 1H, 6-H), 2.37 (ddd, *J_1_* = 14.5 Hz, *J_2_ =* 6.5 Hz, *J_3_ =* 3.7 Hz, 1H, 7-H), 2.31 (dd, *J_1_* = 13.4 Hz, *J_2_ =* 10.0 Hz, 1H, 4-H), 2.24 (s, 9H, 2'-CH_3_, 4'-CH_3_, 6'-CH_3_), 2.09 (ddd, *J_1_* = 14.5 Hz, *J_2_ =* 10.0 Hz, *J_3_ =* 4.1 Hz, 1H, 7-H). ^13^C-NMR (126 MHz, DMSO-D_6_) δ (ppm) = 160.5 (C-3), 138.2 (C-4'), 136.9 (C-2', C-6'), 128.5 (C-3', C-5'), 125.5 (C-1'), 104.4 (C-7a), 55.7 (C-3a), 32.2 (C-7), 25.6 (C-4), 23.6 (C-6), 20.6 (4'-CH_3_), 19.8 (2'-CH_3_, 6'-CH_3_). IR (KBr)
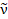
 [cm^-1^] = 3335, 3010, 2960, 2946, 2917, 2852, 1611, 1427, 1319, 1247, 1227, 1108, 1063, 1049, 852, 830. MS (EI) *m/z* (%) = 277 (90) [M^•^]^+^, 260 (37), 172 (100), 145 (78). HRMS (EI) 277.1139 (calculated for C_15_H_19_NO_2_^32^S: 277.1136). Purity (HPLC) 99 % (λ = 210 nm), 99 % (λ = 254 nm).

**3-Mesityl-6,7-dihydro-4*H*-thiopyrano[3,4-*d*]isoxazole (EVP-87)**

C_15_H_17_NOS, M_r_ = 259.37

Prepared following general procedure 5 using (3a*RS*,7a*RS*)-3-mesityl-3a,4,6,7-tetrahydro-7a*H*-thiopyrano[3,4-*d*]isoxazol-7a-ol **(EVP-86)** (0.53 g, 1.9 mmol) to give pale yellow crystals (99 %). mp: 62 °C; ^1^H-NMR (500 MHz, CDCl_3_) δ (ppm) = 6.93 (s, 2H, 3'-H, 5'-H), 3.23 (t, *J* = 1.5 Hz, 2H, 4-H), 3.10 – 3.05 (m, 2H, 7‑H), 2.98 (t, *J* = 5.8 Hz, 2H, 6-H), 2.32 (s, 3H, 4'-CH_3_), 2.08 (s, 6H, 2'-CH_3_, 6'-CH_3_). ^13^C-NMR (126 MHz, CDCl_3_) δ (ppm) = 167.6 (C-7a), 160.3 (C-3), 139.1 (C-4'), 137.3 (C-2', C-6'), 128.4 (C-3', C-5'), 124.9 (C-1'), 110.1 (C-3a), 25.4 (C-6), 25.4 (C-7), 21.6 (C-4), 21.3 (4'-CH_3_), 20.0 (2'‑CH_3_, 6'-CH_3_). IR (Film)
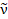
 [cm^-1^] = 3002, 2919, 2857, 1636, 1613, 1439, 1332, 1224, 852. MS (EI) *m/z* (%) = 259 (76) [M^•^]^+^, 203 (79), 147 (100). HRMS (EI) 259.1018 (calculated for C_15_H_17_NO^32^S: 259.1031). This compound is not stable.

**(*RS*)-3-Mesityl-6,7-dihydro-4*H*-thiopyrano[3,4-*d*]isoxazole-5-oxide (EVP-88)**

C_15_H_17_NO_2_S, M_r_ = 275.37

To a stirred solution of 3-mesityl-6,7-dihydro-4*H*-thiopyrano[3,4-*d*]isoxazole **(EVP-87)** (0.10 g, 0.39 mmol) in methanol (2 mL) NaOH (6 M, 3.0 µL, 0.018 mmol) and H_2_O_2_ (30 %, 40 µL, 0.39 mmol) were added. The mixture was heated to 60 °C for 2 h, then diluted with water (20mL) and extracted with dichloromethane (3 × 10 mL). The combined organic layers were dried over magnesium sulfate, filtered and concentrated, FCC using dichloromethane/methanol 95:5 gave a colorless oil (56 %); ^1^H-NMR (500 MHz, DMSO-D_6_) δ (ppm) = 6.99 (s, 2H, 3'-H, 5'-H), 3.65 (dt, *J_1_* = 16.0 Hz, *J_2_* = 1.5 Hz, 1H, 4-H), 3.50 – 3.41 (m, 1H, 6-H), 3.31 (dd, *J_1_* = 16.1 Hz, *J_2_* = 1.8 Hz, 1H, 4-H), 3.26 – 3.10 (m, 2H, 7‑H), 3.00 (ddd, *J_1_* = 13.6 Hz, *J_2_* = 10.4 Hz, *J_3_* = 6.6 Hz, 1H, 6-H), 2.29 (s, 3H, 4'-CH_3_), 2.01 (s, 3H, 6'-CH_3_), 1.95 (s, 3H, 2'CH_3_). ^13^C-NMR (126 MHz, DMSO-D_6_) δ (ppm) = 165.4 (C-7a), 161.5 (C-3), 138.6 (C-4'), 136.7 (C-6'), 136.6 (C-2'), 128.2 (C‑5'), 128.2 (C-3'), 124.4 (C-1'), 104.0 (C-3a), 42.1 (C-4), 41.4 (C-6), 20.7 (4'-CH_3_), 19.5 (6'-CH_3_), 19.4 (2'-CH_3_), 15.5 (C-7). IR (Film)
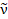
 [cm^-1^] = 2950, 2921, 1637, 1612, 1438, 1335, 1048. MS (EI) *m/z* (%) = 275 (100) [M^•^]^+^, 266 (70), 171 (46), 145 (53). HRMS (EI) 275.0982 (calculated for C_15_H_17_NO_2_^32^S: 275.0980). Purity (HPLC) 99 % (λ = 210 nm), 96 % (λ = 254 nm).

**3-Mesityl-6,7-dihydro-4*H*-thiopyrano[3,4-*d*]isoxazole-5,5-dioxide (EVP-89)**

C_15_H_17_NO_3_S, M_r_ = 291.37

To a stirred solution of 3-mesityl-6,7-dihydro-4*H*-thiopyrano[3,4-*d*]isoxazole **(EVP-87)** (0.10 g, 0.39 mmol) in dichloromethane (3 mL) mCPBA (0.14 g, 0.81 mmol) was added. The mixture was stirred at room temperature for 2 h, then diluted with dichloromethane and washed with a saturated aqueous solution of NaHCO_3_ and water. The organic phase was dried over magnesium sulfate, filtered and the solvent was evaporated in vacuo to give a colorless solid (98%). mp: 189 °C; ^1^H-NMR (400 MHz, CDCl_3_) δ (ppm) = 6.94 (s, 2H, 3'-H, 5'-H), 3.74 (s, 2H, 4-H), 3.48 (t, *J* = 6.1 Hz, 2H, 7-H), 3.36 (t, *J* = 6.1 Hz, 2H, 6-H), 2.32 (s, 3H, 4'-CH_3_), 2.08 (s, 6H, 2'-CH_3_, 6'-CH_3_). ^13^C-NMR (126 MHz, CDCl_3_) δ (ppm) = 163.5 (C-7a), 161.5 (C-3), 139.9 (C-4'), 137.1 (C-2', C-6'), 128.7 (C-3', C-5'), 123.2 (C-1'), 107.3 (C-3a), 47.5 (C-4), 46.8 (C-6), 22.5 (C-7), 21.3 (4'-CH_3_), 20.0 (2'‑CH_3_, 6'-CH_3_). IR (Film)
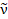
 [cm^-1^] = 3442, 2982, 2957, 2924, 1640, 1611, 1438, 1322, 1286, 1167, 1131, 851. HRMS (EI) 291.0923 (calculated for C_15_H_17_NO_3_^32^S: 291.0929). Purity (HPLC) 98 % (λ = 210 nm), 96 % (λ = 254 nm).

***(E)*-2-(2,4,6-Trimethylbenzylidene)cyclohexan-1-one (EVP-90)**

C_16_H_20_O, M_r_ = 228.34

A mixture of cyclohexanone (0.70 mL, 6.8 mmol), 2,4,6-trimethylbenzaldehyde (504 mg, 3.40 mmol) and NaOH (205 mg, 5.13 mmol) in water (3.5 mL) was refluxed for 40 h, then neutralized with HCl (2 M) and extracted with dichloromethane (3 × 10 mL). The combined organic layers were dried over magnesium sulfate, filtered and concentrated. FCC using hexanes/ethyl acetate 9:1 gave a pale yellow oil (19%). ^1^H-NMR (500 MHz, CDCl_3_) δ (ppm) = 7.35 (s, 1H, 1'-H), 6.87 (s, 2H, 3''-H, 5''-H), 2.54 (t, *J* = 6.8 Hz, 2H, 6-H), 2.29 (s, 3H, 4''-CH_3_), 2.24 (td, *J_1_* = 6.5 Hz, *J_2_* = 2.0 Hz, 2H, 3-H), 2.12 (s, 6H, 2''-CH_3_, 6''-CH_3_), 1.95 – 1.88 (m, 2H, 5-H), 1.71 – 1.65 (m, 2H, 4-H). ^13^C-NMR (101 MHz, CDCl_3_) δ (ppm) = 201.6 (C-1), 139.3 (C-2), 137.1 (C-4''), 135.7 (C-2'', C-6''), 134.9 (C-1'), 132.2 (C-1''), 128.2 (C-3'', C-5''), 40.8 (C-6), 28.4 (C-3), 24.0 (C-4), 24.0 (C-5), 21.1 (4''-CH_3_), 20.2 (2''-CH_3_, 6''-CH_3_). IR (Film)
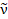
 [cm^-1^] = 2940, 2863, 1689, 1616, 1450, 1267, 1255, 1235, 1138, 851. MS (EI) *m/z* (%) = 228 (80) [M^•^]^+^, 213 (100). HRMS (EI) 228.1508 (calculated for C_16_H_20_O: 228.1514). Purity (HPLC) 82 % (λ = 210 nm), 87 % (λ = 254 nm).

**(3*RS*,3a*RS*,4*RS*,7*SR*,7a*RS*)-3-Mesityl-2-methyloctahydro-4,7-methanobenzo[*d*]isoxazole (EVP-94)**

C_18_H_25_NO, M_r_ = 271.40

A solution trimethyloxonium tetrafluoroborate (150 mg, 1.01 mmol) in dry nitromethane (5 mL) was added dropwise to a stirred solution of (3a*RS*,4*RS*,7*SR*,7a*RS*)-3-mesityl-3a,4,5,6,7,7a-hexahydro-4,7-methanobenzo[*d*]isoxazole **(EVP-2)** (202 mg, 0.791 mmol) under nitrogen atmosphere. After stirring for 48 hours, reaction control by TLC showed absence of the starting material **EVP-2**. The solvent was evaporated in vacuo and the residue was dissolved in dichloromethane (5 mL). Powdered NaBH_4_ (51.3 mg, 1.36 mmol) was added and the suspension was stirred for one hour. Then the excess NaBH_4_ was removed by filtration and the solution was soaked on Isolute®. FCC using hexanes/diethyl ether 9:1 gave a colorless solid (72 %). mp: 76 °C; ^1^H-NMR (500 MHz, CDCl_3_) δ (ppm) = 6.84 (s, 1H, 5'-H), 6.83 (s, 1H, 3'-H), 4.21 (dt, *J_1_* = 6.4 Hz, *J_2_* = 1.4 Hz, 1H, 7a-H), 3.97 (d, *J* = 7.0 Hz, 1H, 3-H), 2.64 (ddd, *J_1_* = 7.0 Hz, *J_2_* = 6.4 Hz, *J_3_* = 1.5 Hz, 1H, 3a‑H), 2.55 (s, 3H, 2'-CH_3_), 2.54 (s, 3H, N-CH_3_), 2.42 (dt, *J_1_* = 5.0 Hz, *J_2_* = 1.1 Hz, 1H, 7-H), 2.36 – 2.32 (m, 1H, 8-H), 2.33 (s, 3H, 6'-CH_3_), 2.26 (s, 3H, 4'-CH_3_), 1.81 – 1.76 (m, 1H, 4-H), 1.49 – 1.41 (m, 1H, 6-H), 1.35 – 1.26 (m, 1H, 5-H), 1.03 (br. d, *J* = 9.7 Hz, 1H, 8‑H), 1.05 – 0.92 (m, 2H, 5-H, 6-H). ^13^C-NMR (126 MHz, CDCl_3_) δ (ppm) = 137.4 (C-2'), 136.9 (C-6'), 136.1 (C-4'), 131.7 (C-3'), 131.0 (C-1'), 129.4 (C‑5'), 84.0 (C-7a), 75.0 (C-3), 56.2 (C-3a), 45.2 (N-CH_3_), 42.2 (C-7), 36.9 (C-4), 35.0 (C-8), 29.5 (C-5), 23.5 (C-6), 22.5 (2'-CH_3_), 21.6 (6'-CH_3_), 20.8 (4'-CH_3_). IR (Film)
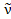
 [cm^-1^] = 2954, 2920, 2870, 1612, 1474, 1455, 1127, 1019, 850. MS (EI) *m/z* (%) = 271 (32) [M^•^]^+^, 162 (100), 152 (25). HRMS (EI) 171.1935 (calculated for C_18_H_25_NO [M^•^]^+^: 171.1931). Purity (HPLC) > 99 % (λ = 210 nm), > 99 % (λ = 254 nm).

**(3a*RS,*4*SR,*7*RS,*7*aSR*)-1-Mesityl-3a,4,5,6,7,7a-hexahydro-1*H*-4,7-methanobenzo[*d*][1,2,3]triazole (EVP-99)**

C_16_H_21_N_3_, M_r_ = 255.37

A solution of 2-azido-1,3,5-trimethylbenzene (117 mg, 0.726 mmol) and norbornene (108 mg, 1.15 mmol) in ethyl acetate (2 mL) was stirred at room temperature for 72 hours. Then the solvent was evaporated in vacuo. FCC using hexanes/ethyl acetate 4:1 gave orange crystals (45%). mp: 64 °C; ^1^H-NMR (500 MHz, CDCl_3_) δ (ppm) = 6.93 (s, 2H, 3'-H, 5'-H), 4.59 (d, *J* = 9.9 Hz, 1H, 3a-H), 3.51 (d, *J* = 9.8 Hz, 1H, 7a-H), 2.82 (d, *J* = 4.5 Hz, 1H, 4-H), 2.36 (d, *J* = 4.7 Hz, 1H, 7-H), 2.30 (s, 3H, 4'-CH_3_), 2.21 (s, 6H, 2'-CH_3_, 6'-CH_3_), 1.65 – 1.56 (m, 1H, 5-H), 1.52 – 1.43 (m, 2H, 6-H, 8-H), 1.33 (dddd, *J_1_* = 12.6 Hz, *J_2_* = 9.0 Hz, *J_3_* = 4.1 Hz, *J_4_* = 2.2 Hz, 1H, 5-H), 1.27 (br. d, *J* = 10.5 Hz, 1H, 8-H), 1.11 (dddd, *J_1_* = 12.9 Hz, *J_2_* = 9.0 Hz, *J_3_* = 4.5 Hz, *J_4_* =2.4 Hz, 1H, 6-H). ^13^C-NMR (101 MHz, CDCl_3_) δ (ppm) = 138.4 (C-4'), 137.4 (C-2', C-6'), 135.7 (C-1'), 129.6 (C-3', C-5'), 86.5 (C-3a), 65.1 (C-7a), 41.8 (C-7), 41.6 (C-4), 33.0 (C-8), 26.1 (C-5), 25.0 (C-6), 21.1 (4'-CH_3_), 18.6 (2'-CH_3_, 6'-CH_3_). IR (KBr)
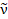
 [cm^-1^] = 2954, 2927, 2872, 1607, 1487, 1464, 1089, 989, 910, 852. MS (EI) *m/z* (%) = 255 (0.4) [M^•^]^+^, 227 (31), 198 (100), 146 (92), 135 (40), 93 (39), 91 (41). HRMS (EI) 255.1733 (calculated for C_16_H_21_N_3_ [M^•^]^+^: 255.1730). Purity (HPLC) > 99 % (λ = 210 nm), 98 % (λ = 254 nm).

**3-Mesityl-4,5,6,7-tetrahydrobenzo[*c*]isoxazole (EVP-103)**

C_16_H_19_NO, M_r_ = 241.33

*n*-Butyllithium (2.5 M in hexane, 3.8 mL, 9.5 mmol) was added dropwise to an ice-cooled solution of cyclohexanone oxime (500 mg, 4.41 mmol) in dry THF (16 mL). After stirring for 30 min at 0 °C and another 30 min at room temperature, the solution was cooled to 0 °C again and methyl mesitoate (390 mg, 2.19 mmol), dissolved in dry THF (4 mL), was added. The solution was stirred for another hour at 0 °C and 15 hours at room temperature. Then a mixture of sulfuric acid (96 %, 1.8 g) and water (5 mL) was added and the mixture was refluxed for one hour. After cooling to ambient temperature the phases were separated, the aqueous phase was extracted with diethyl ether (3 × 20 mL) and the combined organic layers were dried over magnesium sulfate, filtered and concentrated. FCC using hexanes/ethyl acetate 9:1 gave a colorless oil (67%). ^1^H-NMR (500 MHz, CDCl_3_) δ (ppm) = 6.93 (s, 2H, 3'-H, 5'-H), 2.83 (t, *J* = 6.5 Hz, 2H, 7-H), 2.32 (t, *J* = 6.4 Hz, 2H, 4-H), 2.32 (s, 3H, 4'-CH_3_) 2.09 (s, 6H, 2'-CH_3_, 6'-CH_3_), 1.88 – 1.82 (m, 2H, 6-H), 1.77 – 1.70 (m, 2H, 5-H). ^13^C-NMR (126 MHz, CDCl_3_) δ (ppm) = 164.4 (C-3), 160.8 (C-7a), 139.7 (C-4'), 138.2 (C-2', C-6'), 128.3 (C-3', C-5'), 124.9 (C-1'), 112.8 (C-3a), 22.8 (C-5), 22.6 (C-6), 22.0 (C-7), 21.3 (4'-CH_3_), 19.8 (2'‑CH_3_, 6'-CH_3_), 19.6 (C-4). IR (Film)
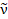
 [cm^-1^] = 2941, 2859, 1637, 1610, 1447, 1416, 1144, 1102, 851. MS (EI) *m/z* (%) = 241 (100) [M^•^]^+^, 173 (26), 147 (48), 119 (26), 91 (25). HRMS (EI) 241.1465 (calculated for C_16_H_19_NO: 241.1467). Purity (HPLC) 98 % (λ = 210 nm), 98 % (λ = 254 nm).

**(*Z*)-2-[Amino(mesityl)methylene]cyclohexan-1-one (EVP-108)**

C_16_H_21_NO, M_r_ = 243.35

To a solution of 3-mesityl-4,5,6,7-tetrahydrobenzo[*d*]isoxazole **(EVP-21)** (865 mg, 3.58 mmol) in ethanol (25 mL), KOH (1.5 g, 27 mmol) and palladium (10% on activated carbon, 100 mg) was added and the mixture was hydrogenated for 60 hours at atmospheric pressure at room temperature. Then the mixture was filtered through silica and the filtrate was diluted with water (50 mL), neutralized with hydrochloric acid (2 M) and extracted with ethyl acetate (3 × 50 mL). The combined organic layers were washed with brine, dried over magnesium sulfate, filtered and concentrated in vacuo to give pale yellow crystals (> 99 %). mp: 154 °C; ^1^H-NMR (400 MHz, CDCl_3_) δ (ppm) = 10.56 (s, 1H, NH), 6.89 (s, 2H, 3''-H, 5''-H), 4.92 (s, 1H, NH), 2.41 (t, *J* = 6.7 Hz, 2H, 6-H), 2.29 (s, 3H, 4''-CH_3_), 2.19 (s, 6H, 2''-CH_3_, 6''-CH_3_), 1.84 (t, *J* = 6.4 Hz, 2H, 3-H), 1.77 – 1.68 (m, 2H, 5-H), 1.59 – 1.51 (m, 2H, 4-H). ^13^C-NMR (101 MHz, CDCl_3_) δ (ppm) = 199.1 (C-1), 161.2 (C-1'), 138.2 (C-4''), 134.5 (C-2'', C-6''), 134.0 (C-1''), 128.5 (C-3'', C-5''), 102.1 (C-2), 38.8 (C-6), 26.4 (C-3), 24.1 (C-4), 23.3 (C-5), 21.2 (4''-CH_3_), 19.1 (2''-CH_3_, 6''-CH_3_). IR (KBr)
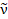
 [cm^-1^] = 3264, 3111, 2939, 2921, 1613, 1585, 1505, 1467, 1409, 1318, 1300, 128, 1159, 852, 696. MS (EI) *m/z* (%) = 243 (34) [M^•^]^+^, 228 (100), 226 (16), 172 (19). HRMS (EI) 243.1627 (calculated for C_16_H_21_NO: 243.1623). Purity (HPLC) 97 % (λ = 210 nm), > 99 % (λ = 254 nm).

**3-Mesityl-4,5,6,7-tetrahydrobenzo[*d*]isothiazole (EVP-109)**

C_16_H_19_NS, M_r_ = 257.40

Phosphorus pentasulfide (150 mg, 0.675 mmol) was added to a stirred mixture of (*Z*)-2-[amino(mesityl)methylene]cyclohexan-1-one **(EVP-108)** (100 mg, 0.411 mmol) and NaHCO_3_ (35 mg, 0.42 mmol) in dry THF (2 mL) and the mixture was stirred for 15 hours at room temperature. Then *p*-chloranil (150 mg, 0.610 mmol) in 3 mL THF was added and the mixture stirred for another 5 hours. Then the mixture was diluted with water (30 mL) and extracted with ethyl acetate (3 × 30 mL). The combined organic layers were dried over magnesium sulfate, filtered and concentrated. FCC using hexanes/ethyl acetate 4:1 gave a yellow oil (> 99 %); ^1^H-NMR (500 MHz, CDCl_3_) δ (ppm) = 6.89 (s, 2H, 3'-H, 5'-H), 2.94 (tt, *J_1_* = 6.3 Hz, *J_2_* = 1.4 Hz, 2H, 7-H), 2.31 (s, 3H, 4'-CH_3_), 2.24 (tt, *J_1_* = 6.1 Hz, *J_2_* = 1.5 Hz, 2H, 4-H), 1.95 (s, 6H, 2'-CH_3_, 6'-CH_3_), 1.89 – 1.83 (m, 2H, 6-H), 1.78 – 1.71 (m, 2H, 5-H). ^13^C-NMR (101 MHz, CDCl_3_) δ (ppm) = 168.4 (C-3), 159.5 (C-7a), 137.8 (C-4'), 136.7 (C-2', C-6'), 132.8 (C-3a), 132.5 (C-1'), 128.2 (C-3', C-5'), 24.0 (C-7), 23.6 (C-4), 22.9 (C-6), 22.4 (C-5), 21.3 (4'-CH_3_), 19.7 (2'-CH_3_, 6'-CH_3_). IR (Film)
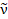
 [cm^-1^] = 3019, 2934, 2861, 1612, 1448, 1378, 1311, 1215. MS (EI) *m/z* (%) = 257 (100) [M^•^]^+^, 256 (34), 242 (60), 224 (38). HRMS (EI) 257.1226 (calculated for C_16_H_19_N^32^S [M^•^]^+^: 257.1233). Purity (HPLC) > 99 % (λ = 210 nm), > 99 % (λ = 254 nm).

**(3*RS*,3a*RS*,4*SR*,7*RS*,7a*SR*)-3-Mesityl-3a,4,5,6,7,7a-hexahydro-3*H*-4,7-methanoindazole (EVP-110)**

C_17_H_22_N_2_, M_r_ = 254.38

Mesitaldehyde (1.00 g, 6.75 mmol) was added to a solution of *p*-toluenesulfonylhydrazide (1.50 g, 8.05 mmol) in glacial acetic acid (3.5 mL) and refluxed for 3 hours. The white precipitate formed upon cooling was collected by filtration, washed with water and dried in vacuo to give 1.93 g mesitaldehyde tosylhydrazone (6.10 mmol, 90 %). Without further purification 1.00 g (3.16 mmol) of this precipitate was suspended in toluene (50 mL) and poured into a solution of benzyltriethylammonium chloride (1.1 g, 4.8 mmol) in aqueous NaOH (14 %, 50 mL). The mixture was stirred for 15 minutes at 90 °C and the phases were separated. The organic phase was washed with water (3 × 30 mL), dried over magnesium sulfate, filtered, and concentrated to a volume of 6.0 mL.
Norbornene (400 mg, 4.25 mmol) was added to 2.0 mL of this crude aryldiazomethane solution and stirred for 15 hours at room temperature. FCC using hexanes/ethyl acetate 4:1 gave bright beige crystals (24%). mp: 56 °C; ^1^H-NMR (500 MHz, CDCl_3_) δ (ppm) = 6.87 (s, 2H, 3'-H, 5'-H), 5.23 (dd, *J_1_* = 5.1 Hz, *J_2_* = 3.4 Hz, 1H, 3-H), 4.87 (dd, *J_1_* = 7.3 Hz, *J_2_* = 3.4 Hz, 1H, 7a-H), 2.98 (d, *J* = 4.6 Hz, 1H, 7-H), 2.27 (s, 3H, 4'-CH_3_), 2.22 (d, *J* = 4.3 Hz, 1H, 4-H), 2.15 (s, 6H, 2'-CH_3_, 6'-CH_3_), 1.81 (ddd, *J_1_* = 7.1 Hz, *J_2_* = 5.1 Hz, *J_3_* = 1.6 Hz, 1H, 3a-H), 1.72 – 1.61 (m, 1H, 6-H), 1.56 – 1.46 (m, 1H, 5-H), 1.46 – 1.36 (m, 1H, 6-H), 1.21 – 1.12 (m, 1H, 5-H), 1.11 (br. d, *J* = 10.8 Hz, 1H, 8-H), 0.83 (br. d, *J* = 10.8 Hz, 1H, 8-H). ^13^C-NMR (101 MHz, CDCl_3_) δ (ppm) = 137.5 (C-4'), 136.7 (C-2', C-6'), 132.7 (C-1'), 130.3 (C-3', C-5'), 99.7 (C-7a), 95.2 (C-3), 46.3 (C-3a), 41.5 (C-4), 39.9 (C-7), 32.4 (C-8), 27.9 (C-5), 26.5 (C-6), 20.9 (4'-CH_3_), 20.5 (2'-CH_3_, 6'-CH_3_). IR (ATR)
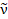
 [cm^-1^] = 3007, 2982, 2950, 2872, 1609, 1547, 1481, 1459, 1294, 1225, 1033, 849. HRMS (ESI) 255.1857 (calculated for C_17_H_23_N_2_^+^ [M+H]^+^: 255.1856). Purity (HPLC) > 99 % (λ = 210 nm), > 99 % (λ = 254 nm).

**(*RS*)-2-[2-Oxo-2-(2,2,4-trimethyl-3,4-dihydroquinolin-1(2*H*)-yl)ethyl]isoindoline-1,3-dione (ML-SA1^29^)**

C_22_H_22_N_2_O_3_, M_r_ = 362.43

Prepared following general procedure 6a using (*RS*)-2,2,4-trimethyl-1,2,3,4-tetrahydroquinoline (237 mg, 1.35 mmol) and *N*-phthalylglycyl chloride (300 mg, 1.34 mmol). FCC using hexanes/ethyl acetate 9:1 gave colorless crystals (71 %). mp: 154 °C; ^1^H-NMR (500 MHz, CDCl_3_) δ (ppm) = 7.86 – 7.81 (m, 2H, 4-H, 7-H), 7.72 – 7.67 (m, 2H, 5-H, 6-H), 7.25 – 7.17 (m, 4H, 5''-H, 6''-H, 7''-H, 8''-H), 4.82 (d, *J* = 16.1 Hz, 1H, 1'-H), 4.04 (d, *J* = 16.2 Hz, 1H, 1'‑H), 2.89 (dqd, *J_1_* = 12.0 Hz, *J_2_* = 6.8 Hz, *J_3_* = 2.7 Hz, 1H, 4''-H), 1.86 (dd, *J_1_* = 12.9 Hz, *J_2_* = 2.7 Hz, 1H, 3''-H), 1.68 (s, 3H, 2''-CH_3_), 1.46 (s, 3H, 2''-CH_3_), 1.35 (d, *J* = 6.8 Hz, 3H, 4''-CH_3_), 1.24 (dd, *J_1_* = 12.9 Hz, J_2_ = 12.0 Hz, 1H, 3''-H). ^13^C-NMR (126 MHz, CDCl_3_) δ (ppm) = 168.1 (C-1, C-3), 167.3 (C-2'), 141.7 (C-4a''), 137.6 (C-8a''), 134.1 (C-5, C‑6), 132.4 (C-3a, C-7a), 126.5 (C-7''), 125.9 (C-6''), 125.2 (C-8''), 123.5 (C-5''), 123.5 (C-4, C-7), 60.3 (C-2''), 51.9 (C-3''), 42.4 (C-1'), 29.3 (C-4''), 27.9 (2''-CH_3_), 25.8 (2''‑CH_3_), 17.0 (4''-CH_3_). IR (ATR)
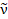
 [cm^-1^] = 3022, 2954, 2933, 2871, 1720, 1666, 1487, 1422, 1396, 1368, 1320, 1306, 1248, 1111, 773, 714. MS (EI) *m/z* (%) = 362 (42) [M^•^]^+^, 160 (89), 146 (70), 85 (63), 83 (100). HRMS (EI) 362.1628 (calculated for C_22_H_22_N_2_O_3_^•+^: 362.1625). Purity (HPLC) > 99 % (λ = 210 nm), > 99 % (λ = 254 nm).

**2-[2-Oxo-2-(2,2,4-trimethylquinolin-1(2*H*)-yl)ethyl]isoindoline-1,3-dione (SF-51^30^)**

C_22_H_20_N_2_O_3_, M_r_ = 360.41

Prepared following general procedure 6a using 2,2,4-trimethyl-1,2-dihydroquinoline (241 mg, 1.39 mmol) and *N*-phthalylglycyl chloride (303 mg, 1.35 mmol mmol). FCC using hexanes/ethyl acetate 9:1 gave beige crystals (27 %). mp: 143 °C; ^1^H-NMR (500 MHz, CDCl_3_) δ (ppm) = 7.86 – 7.81 (m, 2H, 4-H, 7-H), 7.73 – 7.68 (m, 2H, 5-H, 6-H), 7.36 (dd, *J_1_* = 7.8 Hz, *J_2_* = 1.2 Hz, 1H, 8''-H), 7.28 – 7.23 (m, 1H, 7''-H), 7.23 – 7.21 (m, 1H, 5''‑H) 7.20 – 7.15 (m, 1H, 6''-H), 5.58 (q, *J* = 1.5 Hz, 1H, 3''-H), 4.56 (s, 2H, 1'-H), 2.01 (d, *J* = 1.5 Hz, 3H, 4''-CH_3_), 1.49 (s, 6H, 2''-(CH_3_)_2_). ^13^C-NMR (126 MHz, CDCl_3_) δ (ppm) = 168.0 (C-1, C-3), 167.9 (C-2'), 136.4 (C-3''), 135.7 (C-8a''), 134.1 (C-5, C-6), 132.3 (C-3a, C-7a), 130.2 (C-4''), 128.0 (C-4a''), 127.8 (C-7''), 125.7 (C-6''), 124.5 (C‑8''), 123.6 (C-4, C-7), 123.4 (C-5''), 59.0 (C-2''), 42.7 (C-1'), 26.5 (2''-(CH_3_)_2_), 17.9 (4''-CH_3_). IR (ATR)
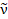
 [cm^-1^] = 3033, 3020, 2968, 2945, 1714, 1673, 1488, 1415, 1392, 1376, 1327, 1300, 1237, 1106, 955, 759, 749, 720. MS (EI) *m/z* (%) = 360 (3) [M^•^]^+^, 345 (48), 160 (100), 158 (84). HRMS (EI) 360.1469 (calculated for C_22_H_20_N_2_O_3_^•+^: 360.1468). Purity (HPLC) 98 % (λ = 210 nm), 99 % (λ = 254 nm).

**(*RS*)-1-[2-Oxo-2-(2,2,4-trimethyl-3,4-dihydroquinolin-1(2*H*)-yl)ethyl]-pyrrolidine-2,5-dione (EVP-146)**

C_18_H_22_N_2_O_3_, M_r_ = 314.39

A mixture of (*RS*)-2-chloro-1-(2,2,4-trimethyl-3,4-dihydroquinolin-1(2*H*)-yl)ethan-1-one **(EVP-149)** (203 mg, 0.806 mmol), succinimide (80.0 mg, 0.807 mmol) and cesium carbonate (263 mg, 0.807 mmol) in dry dimethylformamide (0.6 mL) was heated at 70 °C for 20 hours. After conversion was complete (controlled by TLC), water (20 mL) was added and the mixture was extracted with ethyl acetate (3 × 20 mL). The combined organic layers were washed with brine, dried over sodium sulfate, filtered and concentrated. FCC using hexanes/ethyl acetate 1:1 gave a colorless solid (86 %). mp: 91 °C; ^1^H-NMR (500 MHz, CDCl_3_) δ (ppm) = 7.25 – 7.16 (m, 4H, 5''-H, 6''-H, 7''-H, 8''-H), 4.64 (d, *J* = 15.9 Hz, 1H, 1'-H), 3.85 (d, *J* = 15.9 Hz, 1H, 1'-H), 2.85 (dqd, *J_1_* = 12.9 Hz, *J_2_* = 6.8 Hz, *J_3_* = 2.7 Hz, 1H, 4''‑H), 2.71 (s, 4H, 3-H, 4-H), 1.84 (dd, *J_1_* = 12.7 Hz, *J_2_* = 2.7 Hz, 1H, 3''-H), 1.66 (s, 3H, 2''‑CH_3_), 1.45 (s, 3H, 2''-CH_3_), 1.34 (d, *J* = 6.8 Hz, 3H, 4''-CH_3_), 1.22 (dd, *J_1_* = 12.9 Hz, J_2_ = 12.6 Hz, 1H, 3''-H). ^13^C-NMR (126 MHz, CDCl_3_) δ (ppm) = 176.8 (C-2, C-5), 166.5 (C-2'), 141.7 (C-4a''), 137.5 (C-8a''), 126.5 (C-7''), 125.9 (C-6''), 125.2 (C-8''), 123.5 (C-5''), 60.3 (C-2''), 51.9 (C-3''), 43.0 (C-1'), 29.3 (C‑4''), 28.3 (C-3, C-4), 27.9 (2''-CH_3_), 25.7 (2''-CH_3_), 17.0 (4''-CH_3_). IR (ATR)
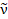
 [cm^-1^] = 3012, 2966, 2935, 2877, 1706, 1675, 1493, 1425, 1406, 1366, 1324, 1308, 1241, 1172, 772. MS (EI) *m/z* (%) = 314 (34) [M^•^]^+^, 160 (100), 146 (41), 112 (35). HRMS (EI) 314.1627 (calculated for C_18_H_22_N_2_O_3_^•+^: 314.1625). Purity (HPLC) 314.1627 (calculated for C_18_H_22_N_2_O_3_^•+^: 314.1625).

**(*RS*)-1-[2-Oxo-2-(2,2,4-trimethyl-3,4-dihydroquinolin-1(2*H*)-yl)ethyl]-1*H*-pyrrole-2,5-dione (EVP-147)**

C_18_H_20_N_2_O_3_, M_r_ = 312.37

(*RS*)-*exo*-2-[2-Oxo-2-(2,2,4-trimethyl-3,4-dihydroquinolin-1(2*H*)-yl)ethyl]-3a,4,7,7a-tetrahydro-1*H*-4,7-epoxyisoindole-1,3(2*H*)-dione **(EVP-177)** (152 mg, 0.400 mmol) was transferred into a sublimation apparatus and heated to 170 °C – 200 °C for 30 min at a pressure of 0.1 mbar. After cooling down, pale yellow crystals were formed (95 %). mp: 126 °C; ^1^H-NMR (500 MHz, CDCl_3_) δ (ppm) = 7.25 – 7.18 (m, 3H, 5''-H, 6''-H, 7''-H), 7.18 – 7.13 (m, 1H, 8''-H), 6.71 (s, 2H, 3-H, 4-H), 4.65 (d, *J* = 16.3 Hz, 1H, 1'-H), 3.87 (d, *J* = 16.3 Hz, 1H, 1'-H), 2.85 (dqd, *J_1_*= 13.4 Hz, J_2_ = 6.8 Hz, J_3_ = 2.7 Hz, 1H, 4''-H), 1.84 (dd, *J_1_* = 12.7 Hz, *J_2_* = 2.7 Hz, 1H, 3''‑H), 1.65 (s, 3H, 2''-CH_3_), 1.45 (s, 3H, 2''-CH_3_), 1.34 (d, *J* = 6.7 Hz, 3H, 4''-CH_3_), 1.22 (dd, *J_1_* = 13.3 Hz, *J_2_* = 12.6 Hz, 1H, 3''-H). ^13^C-NMR (126 MHz, CDCl_3_) δ (ppm) = 170.5 (C-2, C-5), 167.2 (C-2'), 141.7 (C-4a''), 137.5 (C-8a''), 134.5 (C-3, C‑4), 126.5 (C-7''), 125.9 (C-6''), 125.1 (C-8''), 123.6 (C-5''), 60.3 (C-2''), 51.8 (C‑3''), 42.2 (C‑1'), 29.3 (C-4''), 27.9 (2''-CH_3_), 25.7 (2''-CH_3_), 17.0 (4''-CH_3_). IR (ATR)
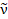
 [cm^-1^] = 3120, 2980, 2953, 2934, 1702, 1666, 1490 1429, 1377, 1308, 1248, 1154, 839, 767, 701. MS (EI) *m/z* (%) = 312 (62) [M^•^]^+^, 160 (85), 146 (100). HRMS (EI) 312.1463 (calculated for C_18_H_20_N_2_O_3_: 312.1474). Purity (HPLC) 98 % (λ = 210 nm), 98 % (λ = 254 nm).

**(*RS*)-2-Hydroxy-1-(2,2,4-trimethyl-3,4-dihydroquinolin-1(2*H*)-yl)ethan-1-one (EVP-147_A)** C_14_H_19_NO_2_, M_r_ = 233.31 and **(*RS*)-2-(2-Oxo-2-(2,2,4-trimethyl-3,4-dihydroquinolin-1(2*H*)-yl)ethyl)benzo[*d*]isothiazol-3(2*H*)-one 1,1-dioxide (EVP-152)** C_21_H_22_N_2_O_4_S, M_r_ = 398.48

A mixture of (*RS*)-2-chloro-1-(2,2,4-trimethyl-3,4-dihydroquinolin-1(2*H*)-yl)ethan-1-one **(EVP-149)** (204 mg, 0.810 mmol), saccharin (150 mg, 0.819 mmol) and cesium carbonate (265 mg, 0.814 mmol) in dry dimethylformamide (0.6 mL) was heated at 70 °C for 20 hours. Then water (20 mL) was added and the mixture was extracted with ethyl acetate (3 × 20 mL). The combined organic layers were washed with brine, dried over sodium sulfate, filtered and concentrated. FCC using a gradient hexanes/ethyl acetate 9:1 -> 4:1 was used to separate not reacted starting material **EVP-149** (eluting first), the hydrolyzed starting material **EVP-147_A** (eluting second) and the coupling product **EVP-152** (eluting last).

**EVP-147_A** was obtained as yellow crystals (32 %). mp: 54 °C; ^1^H-NMR (400 MHz, CDCl_3_) δ (ppm) = 7.25 – 7.19 (m, 2H, 5'-H, 6'-H), 7.19 – 7.12 (m, 1H, 7'-H), 6.99 – 6.95 (m, 1H, 8'-H), 4.46 (dd, *J_1_* = 14.9 Hz, *J_2_* = 4.0 Hz, 1H, 2-H), 3.68 (dd, *J_1_* = 14.9 Hz, *J_2_* = 5.8 Hz, 1H, 2-H), 3.37 (dd, *J_1_* = 5.9 Hz, *J_2_* = 4.1 Hz, 1H, OH), 2.81 – 2.71 (m, 1H, 4'-H), 1.85 (dd, *J_1_* = 12.9 Hz, *J_2_* = 2.8 Hz, 1H, 3'-H), 1.73 (s, 3H, 2'-CH_3_), 1.49 (s, 3H, 2'-CH_3_), 1.33 (d, *J* = 6.8 Hz, 3H, 4'-CH_3_), 1.22 (dd, *J_1_* = 13.0 Hz, *J_2_* = 12.9 Hz, 1H, 3'-H). ^13^C-NMR (126 MHz, CDCl_3_) δ (ppm) = 173.3 (C-1), 141.7 (C-4a'), 136.8 (C-8a'), 126.2 (C-7'), 126.1 (C-6'), 124.7 (C-8'), 123.6 (C-5'), 62.8 (C-2), 60.1 (C-2'), 51.8 (C-3'), 29.3 (C-4'), 27.9 (2'-CH_3_), 26.0 (2'-CH_3_), 17.1 (4'-CH_3_). IR (ATR)
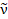
 [cm^-1^] = 3462, 2957, 2926, 1645, 1489, 1453, 1335, 1298, 1078, 771. MS (EI) *m/z* (%) = 233 (65) [M^•^]^+^, 218 (24), 160 (70), 146 (100). HRMS (EI) 233.1411 (calculated for C_14_H_19_NO_2_^•+^: 233.1410). Purity (HPLC) 99 % (λ = 210 nm), 99 % (λ = 254 nm).

**EVP-152** was obtained as a colorless solid (15 %). mp: 185 °C; ^1^H-NMR (400 MHz, CDCl_3_) δ (ppm) = 8.09 – 8.05 (m, 1H, 4-H), 7.93 – 7.89 (m, 1H, 7-H), 7.89 – 7.79 (m, 2H, 5-H, 6-H), 7.29 – 7.21 (m, 4H, 5''-H, 6''-H, 7''-H), 7.18 – 7.12 (m, 1H, 8''-H), 4.84 (d, *J* = 16.6 Hz, 1H, 1'-H), 4.05 (d, *J* = 16.7 Hz, 1H, 1'-H), 2.90 (dqd, *J_1_* = 13.6 Hz, *J_2_* = 6.8, *J_3_* = 2.6 Hz, 1H, 4''-H), 1.87 (dd, *J_1_* = 12.7 Hz, *J_2_* = 2.6 Hz, 1H, 3''-H), 1.70 (s, 3H, 2''-CH_3_), 1.48 (s, 3H, 2''-CH_3_), 1.36 (d, *J* = 6.7 Hz, 3H, 4''-CH_3_), 1.25 (dd, *J* = 13.6 Hz, *J_2_* = 12.4 Hz, 1H, 3''‑H). ^13^C-NMR (101 MHz, CDCl_3_) δ (ppm) = 165.4 (C-2'), 159.2 (C-3), 141.9 (C-4a''), 138.0 (C-7a), 137.4 (C-8a''), 134.9 (C-6), 134.4 (C-5), 127.6 (C-3a), 126.6 (C-7''), 126.2 (C-6''), 125.5 (C-4), 125.0 (C-8''), 123.8 (C-5''), 121.2 (C-7), 60.5 (C-2''), 51.8 (C-3''), 42.5 (C-1'), 29.4 (C-4''), 27.9 (2''-CH_3_), 25.7 (2''-CH_3_), 17.0 (4''-CH_3_). IR (ATR)
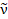
 [cm^-1^] = 3091, 3066, 2958, 2934, 2876, 1732, 1657, 1335, 1316, 1302, 1244, 1181, 762, 749, 672, 581. MS (EI) *m/z* (%) = 398 (50) [M^•^]^+^, 196 (69), 160 (85), 146 (100). HRMS (EI) 398.1298 (calculated for C_21_H_22_N_2_O_4_S ^•+^: 398.1295). Purity (HPLC) > 99 % (λ = 210 nm), > 99 % (λ = 254 nm).

**(*RS*)-2-Chloro-1-(2,2,4-trimethyl-3,4-dihydroquinolin-1(2*H*)-yl)ethan-1-one (EVP-149)**

C_14_H_18_ClNO, M_r_ = 251.75

Potassium carbonate (3.03 g, 21.9 mmol) was added to an ice-cooled solution of (*RS*)-2,2,4-trimethyl-1,2,3,4-tetrahydroquinoline (3.20 g, 18.3 mmol) in acetone (25 mL). Chloroacetyl chloride (4.5 mL, 56 mmol) was added dropwise over an hour and the mixture stirred for another 15 hours while slowly warming to room temperature. Then the mixture was poured into water (50 mL) and extracted with ethyl acetate (3 × 50 mL). The combined organic layers were washed with brine, dried over sodium sulfate, filtered and concentrated. FCC using hexanes/ethyl acetate 19:1 gave a beige solid (36 %). mp: 79 °C; ^1^H-NMR (400 MHz, CDCl_3_) δ (ppm) = 7.24 – 7.15 (m, 3H, 5'-H, 6'-H, 7'-H), 7.00 – 6.94 (m, 1H, 8'-H), 4.03 – 4.05 (m, 2H, 2-H), 2.78 (dqd, *J_1_* = 13.3 Hz, *J_2_* = 6.8 Hz, *J_3_* = 2.7 Hz, 1H, 4'-H), 1.88 (dd, *J_1_* = 12.8 Hz, *J_2_* = 2.7 Hz, 1H, 3'-H), 1.69 (s, 3H, 2'-CH_3_), 1.50 (s, 3H, 2'-CH_3_), 1.34 (d, *J* = 6.8 Hz, 3H, 4'-CH_3_), 1.24 (dd, *J_1_* = 13.2 Hz, *J_2_* = 12.6 Hz, 1H, 3'-H). ^13^C-NMR (101 MHz, CDCl_3_) δ (ppm) = 168.3 (C-1), 141.2 (C-4a'), 138.3 (C-8a'), 126.5 (C-7'), 125.9 (C-6'), 124.3 (C-8'), 123.5 (C-5'), 59.9 (C-2'), 51.8 (C-3'), 44.2 (C-2), 29.1 (C-4'), 27.8 (2'-CH_3_), 25.5 (2'-CH_3_), 16.9 (4'-CH_3_). IR (ATR)
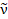
 [cm^-1^] = 3055, 2970, 2935, 2876, 1659, 1490, 1456, 1350, 1302, 1233, 768, 702. MS (EI) *m/z* (%) = 253 (12) [(^37^Cl) M^•^]^+^, 251 (34) [(^35^Cl) M^•^]^+^, 236 (65), 160 (100), 144 (25). HRMS (EI) 251.1075 (calculated for C_14_H_18_^35^ClNO ^•+^: 251.1071). Purity (HPLC) > 99 % (λ = 210 nm), > 99 % (λ = 254 nm).

**2-Chloro-1-(2,2,4-trimethylquinolin-1(2*H*)-yl)ethan-1-one (EVP-150)**

C_14_H_16_ClNO, M_r_ = 249.74

Potassium carbonate (2.42 g, 17.5 mmol) was added to an ice-cooled solution of 2,2,4-trimethyl-1,2-dihydroquinoline (2.35 g, 13.6 mmol) in acetone (20 mL). Chloroacetyl chloride (3.2 mL, 40 mmol) was added dropwise over an hour and stirred for another 15 hours while slowly warming to room temperature. Then the mixture was poured into water (50 mL) and extracted with ethyl acetate (3 × 50 mL). The combined organic layers were washed with brine, dried over sodium sulfate, filtered and concentrated. FCC using hexanes/ethyl acetate 19:1 gave a grey solid (45 %). mp: 95 °C; ^1^H-NMR (400 MHz, CDCl_3_) δ (ppm) = 7.26 – 7.23 (m, 1H, 5'-H), 7.21 – 7.15 (m, 2H, 6'-H, 7'-H), 6.89 – 6.85 (m, 1H, 8'-H), 5.53 (q, *J* = 1.5 Hz, 1H, 3'-H), 4.10 (s, 2H, 2-H), 2.04 (d, *J* = 1.5 Hz, 3H, 4'‑CH_3_), 1.52 (s, 6H, 2'-(CH_3_)_2_). ^13^C-NMR (101 MHz, CDCl_3_) δ (ppm) = 168.8 (C-1), 136.2 (C-8a'), 135.5 (C-3'), 129.2 (C-4a'), 127.9 (C-4'), 127.7 (C-7'), 125.5 (C-6'), 123.5 (C-5'), 122.6 (C-8'), 58.7 (C-2'), 44.2 (C-2), 26.2 (2'-(CH_3_)_2_), 18.0 (4'-CH_3_). IR (ATR)
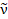
 [cm^-1^] = 3060, 3015, 2973, 2937, 1675, 1491, 1454, 1360, 1341, 1231, 763, 750, 706. MS (EI) *m/z* (%) = 251 (0.5) [(^37^Cl) M^•^]^+^, 249 (2) [(^35^Cl) M^•^]^+^, 234 (32), 158 (100). HRMS (EI) 249.0924 (calculated for C_14_H_16_^35^ClNO ^•+^: 249.0915). Purity (HPLC) > 99 % (λ = 210 nm), > 99 % (λ = 254 nm).

**1-[2-Oxo-2-(2,2,4-trimethylquinolin-1(2*H*)-yl)ethyl]pyrrolidine-2,5-dione (EVP-151)**

C_18_H_20_N_2_O_3_, M_r_ = 312.37

A mixture of 2-chloro-1-(2,2,4-trimethylquinolin-1(2*H*)-yl)ethan-1-one **(EVP-150)** (225 mg, 0.901 mmol), succinimide (140 mg, 1.41 mmol) and cesium carbonate (257 mg, 0.789 mmol) in dry dimethylformamide (0.6 mL) was heated at 70 °C for 15 h. After reaction was complete (controlled by TLC), water (20 mL) was added and the mixture was extracted with ethyl acetate (3 × 20 mL). The combined organic layers were washed with brine, dried over sodium sulfate, filtered and concentrated. FCC using hexanes/ethyl acetate 1:1 gave a colorless solid (63 %). mp: 125 °C; ^1^H-NMR (400 MHz, CDCl_3_) δ (ppm) = 7.32 – 7.29 (m, 1H, 8''-H), 7.27 – 7.24 (m, 1H, 5''-H), 7.24 – 7.15 (m, 2H, 6''-H, 7''-H), 5.57 (q, *J* = 1.5 Hz, 1H, 3''-H), 4.37 (s, 2H, 1'-H), 2.73 (s, 4H, 3-H, 4-H), 2.05 (d, *J* = 1.5 Hz, 3H, 4''-CH_3_), 1.48 (s, 6H, 2''-(CH_3_)_2_). ^13^C-NMR (101 MHz, CDCl_3_) δ (ppm) = 176.8 (C-2, C-5), 167.0 (C-2'), 136.4 (C-3''), 135.5 (C-8a''), 130.2 (C‑4a''), 128.0 (C-4''), 127.7 (C-6''), 125.7 (C-7''), 124.6 (C-8''), 123.4 (C-5''), 59.1 (C-2''), 43.2 (C-1'), 28.4 (C-3, C-4), 26.5 (2''-(CH_3_)_2_), 17.9 (4''-CH_3_). IR (ATR)
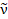
 [cm^-1^] = 3059, 3011, 2996, 2983, 2944, 1710, 1672, 1491, 1432, 1403, 1344, 1287, 1252, 1166, 760. MS (EI) *m/z* (%) = 312 (2) [M^•^]^+^, 297 (29), 158 (100), 112 (19). HRMS (EI) 312.1478 (calculated for C_18_H_20_N_2_O_3_^•+^: 312.1468). Purity (HPLC) 98 % (λ = 210 nm), 99 % (λ = 254 nm).

**(*RS*)-2-[2-Oxo-2-(2,2,4,7-tetramethyl-3,4-dihydroquinolin-1(2*H*)-yl)ethyl]isoindoline-1,3-dione (EVP-154)**

C_23_H_24_N_2_O_3_, M_r_ = 376.46

Prepared following general procedure 6a using (*RS*)-2,2,4,7-tetramethyl-1,2,3,4-tetrahydroquinoline (0.21 mL, 1.1 mmol) and *N*-phthalylglycyl chloride (0.24 g, 1.1 mmol). FCC using hexanes/ethyl acetate 9:1 gave a colorless solid (17 %). mp: 148 °C; ^1^H-NMR (400 MHz, CDCl_3_) δ (ppm) = 7.88 – 7.78 (m, 2H, 4-H, 7-H), 7.73 – 7.66 (m, 2H, 5-H, 6-H), 7.08 – 7.03 (m, 2H, 5''-H, 8''-H), 6.97 (d, *J* = 7.6 Hz, 1H, 6''-H), 4.78 (d, *J* = 16.2 Hz, 1H, 1'-H), 4.11 (d, *J* = 16.2 Hz, 1H, 1'-H), 2.90 – 2.78 (m, 1H, 4''-H), 2.37 (s, 3H, 7''-CH_3_), 1.84 (dd, *J_1_* = 12.8 Hz, *J_2_* = 2.7 Hz, 1H, 3''-H), 1.67 (s, 3H, 2''-CH_3_), 1.45 (s, 3H, 2''-CH_3_), 1.31 (d, *J* = 6.8 Hz, 3H, 4''-CH_3_), 1.20 (dd, *J_1_* = 12.7 Hz, *J_2_* = 12.0 Hz, 1H, 3''-H). ^13^C-NMR (101 MHz, CDCl_3_) δ (ppm) = 168.0 (C-1, C-3), 167.3 (C-2'), 138.6 (C-4a''), 137.5 (C-7''), 136.3 (C-8a''), 134.0 (C-5, C-6), 132.4 (C-3a, C-7a), 126.4 (C-6''), 125.8 (C-8''), 123.5 (C-4, C-7), 123.3 (C-5''), 60.3 (C-2''), 52.0 (C-3''), 42.5 (C-1'), 29.0 (C-4''), 28.0 (2''-CH_3_), 25.8 (2''-CH_3_), 21.1 (7''-CH_3_), 17.1 (4''-CH_3_). IR (ATR)
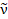
 [cm^-1^] = 2956, 2931, 2873, 1710, 1658, 1428, 1397, 1380, 1312, 1114, 714. HRMS (ESI) 377.1861 (calculated for C_23_H_25_N_2_O_3_^+^ [M+H]^+^: 377.1860). Purity (HPLC) > 99 % (λ = 210 nm).

**2-[2-(3,4-Dihydroisoquinolin-2(1*H*)-yl)-2-oxoethyl]isoindoline-1,3-dione (EVP-155)**

C_19_H_16_N_2_O_3_, M_r_ = 320.35

Prepared following general procedure 6b using 1,2,3,4-tetrahydroisoquinoline (0.20 mL, 1.6 mmol) and *N*-phthalylglycyl chloride (0.35 g, 1.6 mmol) to give a colorless solid (51 %). mp: 211 °C; ^1^H-NMR (400 MHz, C_2_D_2_Cl_4_, 353K) δ (ppm) = 7.90 – 7.73 (m, 2H, 4-H, 7-H), 7.72 – 7.62 (m, 2H, 5-H, 6-H), 7.24 – 7.00 (m, 4H, 5''-H, 6''-H, 7''-H, 8''-H), 4.64 (s, 2H, 1''-H), 4.48 (s, 2H, 1'-H), 3.72 (t, *J* = 6.2 Hz, 2H, 3''-H), 3.11 – 2.61 (m, 2H, 4''-H). ^13^C-NMR (101 MHz, C_2_D_2_Cl_4_, 353K) δ (ppm) = 167.8 (C-1, C-3), 164.6 (C-2'), 134.8 (C-8a''), 134.0 (C-5, C‑6), 132.8 (C-4a''), 132.3 (C-3a, C-7a), 128.4 (C-5''), 127.0 (C-7'') 126.7 (C-8'') 126.4 (CH, C-6''), 123.4 (C-4, C-7), 44.8 (C-1''), 42.7 (C‑3''), 39.3 (C-1'), 29.0 (C-4''). IR (ATR)
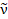
 [cm^-1^] = 3031, 2933, 1771, 1710, 1659, 1469, 1449, 1424, 1395, 1232, 1114, 954, 756, 718. MS (EI) *m/z* (%) = 320 (17) [M^•^]^+^,160 (45), 132 (100). HRMS (EI) 320.1159 (calculated for C_19_H_16_N_2_O_3_^•+^: 320.1155). Purity (HPLC) > 99 % (λ = 210 nm).

**2-[2-(3,4-Dihydroquinolin-1(2*H*)-yl)-2-oxoethyl]isoindoline-1,3-dione (EVP-156)**

C_19_H_16_N_2_O_3_, M_r_ = 320.35

Prepared following general procedure 6b using 1,2,3,4-tetrahydroquinoline (0.20 mL, 1.6 mmol) and *N*-phthalylglycyl chloride (0.36 g, 1.6 mmol) to give a colorless solid (48 %). mp: 193 °C; ^1^H-NMR (500 MHz, CDCl_3_) δ (ppm) = 7.86 (m, 2H, 4-H, 7-H), 7.71 (m, 2H, H-5 H-6), 7.49-7.29 (m, 1H, 6''‑H), 7.25-7.12 (m, 3H, 5''-H, 7''-H, 8''-H), 4.59 (s, 2H, 1'-H), 3.82 (t, *J* = 6.6 Hz, 2H, 2''-H), 2.78 (t, *J* = 6.7 Hz, 2H, 4''-H), 2.00 (tt, *J_1_* = 6.7 Hz, *J_2_* = 6.6 Hz, 2H, 3''-H). ^13^C-NMR (126 MHz, CDCl_3_) δ (ppm) = 168.1 (C-1, C-3), 165.6 (C-2'), 138.1 (C-8a''), 134.2 (C-4a''), 134.1 (C-5, C‑6), 132.4 (C-3a, C-7a), 128.9 (C-5''), 126.6 (C-7''), 126.2 (C-8''), 124.5 (C-6''), 123.6 (C-4, C-7), 43.7 (C-2''), 40.4 (C-1'), 26.9 (C-4''), 23.9 (C-3''). IR (ATR)
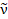
 [cm^-1^] = 3085, 3028, 2934, 1769, 1719, 1651, 1496, 1424, 1386, 1335, 1254, 1116, 955, 722, 711. MS (EI) *m/z* (%) = 320 (60) [M^•^]^+^, 160 (100), 133 (76), 132 (52). HRMS (EI) 320.1155 (calculated for C_19_H_16_N_2_O_3_^•+^: 320.1155). Purity (HPLC) > 99 % (λ = 210 nm).

**2-[2-Oxo-2-(10*H*-phenoxazin-10-yl)ethyl]isoindoline-1,3-dione (EVP-157)**

C_22_H_14_N_2_O_4_, M_r_ = 370.36

Prepared following general procedure 6a using phenoxazine (0.17 g, 0.93 mmol) and *N*-phthalylglycyl chloride (0.21 g, 0.94 mmol). FCC using hexanes/ethyl acetate 4:1 followed by crystallization from hexanes gave a colorless solid (29 %). mp: 184 °C; ^1^H-NMR (400 MHz, CDCl_3_) δ (ppm) = 7.90 – 7.83 (m, 2H, 4-H, 7-H), 7.76 – 7.67 (m, 2H, 5-H, 6-H), 7.57 (dd, *J_1_* = 7.9 Hz, *J_2_* = 1.4 Hz, 2H, 1''-H, 9''-H), 7.24 (ddd, *J_1_* = 8.2 Hz, *J_2_* = 7.2 Hz, *J_3_* = 1.5 Hz, 2H, 3''-H, 7''-H), 7.18 (dd, *J_1_* = 8.2 Hz, *J_2_* = 1.6 Hz, 2H, 4''-H, 6''-H), 7.14 (ddd, *J_1_* = 7.9 Hz, *J_2_* = 7.1 Hz, *J_3_* = 1.6 Hz, 2H, 2''-H, 8''-H), 4.75 (s, 2H, 1'-H). ^13^C-NMR (101 MHz, CDCl_3_) δ (ppm) = 167.9 (C-1, C-3), 165.4 (C-2'), 151.2 (C-4a'', C-5a''), 134.3 (C-5, C-6), 132.3 (C-3a, C-7a), 128.4 (C-9a'', C-10a''), 127.7 (C-3'', C‑7''), 124.9 (C-1'', C-9''), 123.7 (C-2'', C-8''), 123.7 (C-4, C-7), 117.5 (C-4'', C-6''), 40.2 (C-1'). IR (ATR)
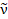
 [cm^-1^] = 1714, 1689, 1480, 1417, 1393, 1299, 1267, 752, 714. HRMS (ESI) 371.1028 (calculated for C_22_H_15_N_2_O_4_^+^ [M+H]^+^: 371.1026). Purity (HPLC) > 99 % (λ = 210 nm).

**2-[2-Oxo-2-(10*H*-phenothiazin-10-yl)ethyl]isoindoline-1,3-dione (EVP-158)**

C_22_H_14_N_2_O_3_S, M_r_ = 386.43

Prepared following general procedure 6a using phenothiazine (0.20 g, 1.0 mmol) and *N*-phthalylglycyl chloride (0.23 g, 1.0 mmol). FCC using hexanes/ethyl acetate 4:1 followed by crystallization from hexanes gave a purple solid (11 %). mp: 238 °C (lit. 232-234 °C^10^, 232-236 °C^31^); ^1^H-NMR (500 MHz, CDCl_3_) δ (ppm) = 7.88 – 7.83 (m, 2H, 4-H, 7-H), 7.74 – 7.68 (m, 2H, 5-H, 6-H), 7.63 (dd, *J_1_* = 8.0 Hz, *J_2_* = 1.3 Hz, 2H, 1''-H, 9''-H), 7.48 (dd, *J_1_* = 7.8 Hz, *J_2_* = 1.5 Hz, 2H, 4''-H, 6''-H), 7.35 (ddd, *J_1_* = 7.9 Hz, *J_2_* = 7.7 Hz, *J_3_* = 1.5 Hz, 2H, 2''-H, 8''-H), 7.27 (ddd, *J_1_* = 7.7 Hz, *J_2_* = 7.6 Hz, *J_3_* = 1.4 Hz, 2H, 3''-H, 7''-H), 4.57 (s, 2H, 1'-H). ^13^C-NMR (126 MHz, CDCl_3_) δ (ppm) = 167.9 (C-1, C-3), 165.4 (C-2'), 137.8 (C-9a'', C-10a''), 134.2 (C-5, C-6), 133.5 (C-4a'', C-5a’’), 132.3 (C-3a, C-7a), 128.4 (C-4'', C‑6''), 127.5 (C-3'', C-7’’), 127.3 (C-2'', C-8''), 127.0 (C-1'', C-9''), 123.7 (C-4, C-7), 40.3 (C-1'). IR (ATR)
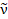
 [cm^-1^] = 2367, 1712, 1697, 1457, 1419, 1363, 1276, 1258, 1106, 948, 763, 714. HRMS (ESI) 387.0798 (calculated for C_22_H_15_N_2_O_3_S^+^ [M+H]^+^: 387.0798). Purity (HPLC) > 99 % (λ = 210 nm).

***N*,*N*-Dicyclohexyl-2-(1,3-dioxoisoindolin-2-yl)acetamide (EVP-159)**

C_22_H_28_N_2_O_3_, M_r_ = 368.48

Prepared following general procedure 6a using *N,N*-dicyclohexylamine (0.20 mL, 1.0 mmol) and *N*-phthalylglycyl chloride (0.23 g, 1.0 mmol). FCC using hexanes/ethyl acetate 4:1 and dichloromethane/methanol 19:1 followed by crystallization from hexanes gave a colorless solid (6 %). mp: 183 °C; ^1^H-NMR (400 MHz, CDCl_3_) δ (ppm) = δ (ppm) = 7.88 – 7.82 (m, 2H, 4'''-H, 7'''-H), 7.72 – 7.66 (m, 2H, 5'''-H, 6'''-H), 4.44 (s, 2H, 2-H), 3.49 – 3.40 (m, 1H, 1'-H), 3.15 – 2.89 (m, 1H, 1''-H), 2.47 – 2.19 (m, 2H, 2''-H, 6''-H), 1.94 – 1.83 (m, 4H, 2'-H, 3'-H, 5'-H, 6'-H), 1.78 – 1.66 (m, 3H, 4'‑H, 3''-H, 5''-H), 1.62 – 1.44 (m, 5H, 2'-H, 6'-H, 2''-H, 4''-H, 6''‑H), 1.41 – 1.28 (m, 2H, 3'-H, 5'-H), 1.23 – 1.03 (m, 4H, 4'-H, 3''-H, 4''-H, 5''‑H). ^13^C-NMR (101 MHz, CDCl_3_) δ (ppm) = 168.3 (C-1''', C-3'''), 163.9 (C-1), 134.0 (C-5''', C-6'''), 132.5 (C-3a''', C‑7a'''), 123.6 (C-4''', C-7'''), 57.4 (C-1'), 56.5 (C-1''), 40.5 (C‑2), 31.4 (C-2', C-6'), 30.0 (C-2'', C-6''), 26.6 (C-3'', C-5''), 26.1 (C‑3', C-5'), 25.4 (C-4''), 25.3 (C-4'). IR (ATR)
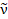
 [cm^-1^] = 3060, 3001, 2980, 2930, 2845, 1722, 1650, 1442, 1422, 1387, 1301, 1109, 727, 714. MS (EI) *m/z* (%) = 368 (27) [M^•^]^+^, 285 (24), 208 (38), 161 (36), 160 (100), 126 (50), 98 (23), 83 (81). HRMS (EI) 368.2092 (calculated for C_22_H_28_N_2_O_3_^•+^: 368.2094). Purity (HPLC) > 99 % (λ = 210 nm).

**2-[2-(Indolin-1-yl)-2-oxoethyl]isoindoline-1,3-dione (EVP-160)**

C_18_H_14_N_2_O_3_, M_r_ = 306.32

Prepared following general procedure 6b using indoline (0.20 mL, 1.8 mmol) and *N*-phthalylglycyl chloride (0.39 g, 1.7 mmol) to give a colorless solid (25 %). mp: 253 °C; ^1^H-NMR (500 MHz, CD_2_Cl_2_) δ (ppm) = 8.05 (d, *J* = 8.1 Hz, 1H, 7''-H), 7.92-7.86 (m, 2H, 4-H, 7-H), 7.81 – 7.74 (m, 2H, 5-H, 6-H), 7.24 (d, *J* = 7.2 Hz, 1H, 4''-H), 7.16 (dd, *J_1_* = 8.1 Hz, *J_2_* = 7.7 Hz, 1H, 6''-H), 7.04 (dd, *J_1_* = 7.7 Hz, *J_2_* = 7.2 Hz, 1H, 5''-H), 4.54 (s, 2H, 1'-H), 4.19 (t, *J* = 8.4 Hz, 2H, 2''-H), 3.29 (t, *J* = 8.4 Hz, 2H, 3''-H). ^13^C-NMR (126 MHz, CD_2_Cl_2_) δ (ppm) = 168.4 (C-1, C-3), 164.0 (C-2'), 143.2 (C-7a''), 134.7 (C-5, C‑6), 132.8 (C-3a, C-7a), 131.7 (C-3a''), 128.0 (C-6''), 125.2 (C-4''), 124.6 (C-5''), 124.0 (C-4, C-7), 117.2 (C-7''), 47.6 (C-2''), 41.1 (C-1'), 28.8 (C-3''). IR (ATR)
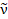
 [cm^-1^] = 1710, 1667, 1484, 1428, 1390, 1333, 1305, 1289, 1110, 955, 761, 723, 714. MS (EI) *m/z* (%) = 306 (59) [M^•^]^+^, 160 (100), 119 (78), 118 (30). HRMS (EI) 306.0999 (calculated for C_18_H_14_N_2_O_3_^•+^: 306.0999). Purity (HPLC) > 99 % (λ = 210 nm).

**2-(1,3-Dioxoisoindolin-2-yl)-*N*-methyl-*N*-phenylacetamide (EVP-161)**

C_17_H_14_N_2_O_3_, M_r_ = 294.31

Prepared following general procedure 6b using *N*-methylaniline (0.20 mL, 1.8 mmol) and *N*-phthalylglycyl chloride (0.41 g, 1.8 mmol) to give a colorless solid (46 %). mp: 179 °C (lit. 176-178 °C ^9^, 177 °C^32^); ^1^H-NMR (500 MHz, CDCl_3_) δ (ppm) = 7.87 – 7.82 (m, 2H, 4'-H, 7'-H), 7.73 – 7.68 (m, 2H, 5'-H, 6'-H), 7.53 – 7.47 (m, 2H, 3''-H, 5''-H), 7

.44 – 7.36 (m, 3H, 2''-H, 4''-H, 6''-H), 4.18 (s, 2H, 2-H), 3.30 (s, 3H, N-CH_3_). ^13^C-NMR (126 MHz, CDCl_3_) δ (ppm) = 168.1 (C-1', C-3'), 165.8 (C-1), 142.5 (C-1''), 134.1 (C-5', C-6'), 132.4 (C-3a', C-7a'), 130.4 (C-3'', C-5''), 128.7 (C-4''), 127.6 (C-2'', C-6''), 123.6 (C-4', C-7'), 39.9 (C-2), 37.9 (N-CH_3_). IR (ATR)
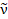
 [cm^-1^] = 1770, 1721, 1668, 1596, 1494, 1468, 1420, 1384, 1325, 1275, 1118, 954, 778, 768, 715, 698. MS (EI) *m/z* (%) = 294 (54) [M^•^]^+^, 160 (100), 134 (78), 107 (58). HRMS (EI) 294.0999 (calculated for C_17_H_14_N_2_O_3_^•+^: 294.0999). Purity (HPLC) > 99 % (λ = 210 nm).

***N*-Cyclohexyl-2-(1,3-dioxoisoindolin-2-yl)-*N*-phenylacetamide (EVP-164)**

C_22_H_22_N_2_O_3_, M_r_ = 362.43

Prepared following general procedure 6a using *N*-cyclohexylaniline (0.20 mL, 1.1 mmol) and *N*-phthalylglycyl chloride (0.25 g, 1.1 mmol). FCC using hexanes/ethyl acetate 4:1 gave a colorless solid (57 %). mp: 193 °C; ^1^H-NMR (500 MHz, CDCl_3_) δ (ppm) = 7.87 – 7.80 (m, 2H, 4''-H, 7''-H), 7.73 – 7.66 (m, 2H, 5''-H, 6''-H), 7.54 – 7.41 (m, 3H, 3'''-H, 4'''-H, 5'''-H), 7.32 – 7.28 (m, 2H, 2'''-H, 6'''-H), 4.52 (tt, *J_1_* = 12.2 Hz, *J_2_* = 3.6 Hz, 1H, 1'-H), 3.99 (s, 2H, 2-H), 1.91 – 1.83 (m, 2H, 2'-H), 1.76 – 1.67 (m, 2H, 5'-H), 1.58 – 1.51 (m, 1H, 4'-H), 1.40 – 1.28 (m, 2H, 3'-H), 1.14 – 1.02 (m, 2H, 6'-H), 0.97 – 0.87 (m, 1H, 4'-H). ^13^C-NMR (126 MHz, CDCl_3_) δ (ppm) = 168.2 (C-1'', C-3''), 165.2 (C-1), 137.8 (C-1'''), 134.0 (C-5'', C-6''), 132.4 (C-3a'', C-7a''), 130.5 (C-2''', C-6'''), 129.8 (C-3''', C-5'''), 129.1 (C-4'''), 123.5 (C-4'', C-7''), 55.4 (C-1'), 40.6 (C-2), 31.5 (C-2', C-6'), 25.8 (C-3', C-5'), 25.4 (C-4'). IR (ATR)
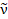
 [cm^-1^] = 2943, 2859, 1717, 1662, 1415, 1389, 1295, 1107, 951, 729, 718, 711. MS (EI) *m/z* (%) = 362 (10) [M^•^]^+^, 280 (89), 174 (30), 161 (48), 160 (100), 93 (22), 83 (46). HRMS (EI) 362.1624 (calculated for C_22_H_22_N_2_O_3_^•+^: 362.1625). Purity (HPLC) > 99 % (λ = 210 nm).

**(*RS*)-2-[2-(6-Ethoxy-2,2,4-trimethyl-3,4-dihydroquinolin-1(2*H*)-yl)-2-oxoethyl]isoindoline-1,3-dione (EVP-166)**

C_24_H_26_N_2_O_4_, M_r_ = 406.48

Prepared following general procedure 6a using (*RS*)-6-ethoxy-2,2,4-trimethyl-1,2,3,4-tetrahydroquinoline (0.19 g, 0.87 mmol) and *N*-phthalylglycyl chloride (0.21 g, 0.94 mmol). FCC using hexanes/ethyl acetate 4:1 gave a colorless solid (28 %). mp: 166 °C; ^1^H-NMR (500 MHz, CDCl_3_) δ (ppm) = 7.87 – 7.80 (m, 2H, 4-H, 7-H), 7.73 – 7.67 (m, 2H, 5-H, 6-H), 7.14 (d, *J* = 8.5 Hz, 1H, 8''-H), 6.77 (dd, *J_1_* = 2.7 Hz, *J_2_* = 0.8 Hz, 1H, 5''-H), 6.73 (dd, *J_1_* = 8.6 Hz, *J_2_* = 2.7 Hz, 1H, 7''-H), 4.78 (d, *J* = 16.0 Hz, 1H, 1'-H), 4.08 – 3.99 (m, 3H, 1'-H, O-CH_2_), 2.90 – 2.82 (m, 1H, 4''-H), 1.83 (dd, *J_1_* = 12.9 Hz, *J_2_* = 2.8 Hz, 1H, 3''-H), 1.68 (s, 3H, 2''‑CH_3_), 1.43 (t, *J* = 7.0 Hz, 3H, O-CH_2_-**CH_3_**), 1.43 (s, 3H, 2''-CH_3_), 1.32 (d, *J* = 6.8 Hz, 3H, 4''-CH_3_), 1.21 (dd, *J_1_* = 12.9 Hz, *J_2_* = 12.4 Hz, 1H, 3''-H). ^13^C-NMR (126 MHz, CDCl_3_) δ (ppm) = 168.1 (C-1, C-3), 166.7 (C-2'), 157.4 (C-6''), 143.5 (C-4a''), 134.0 (C-5, C-6), 132.4 (C-3a, C-7a), 130.4 (C-8a''), 126.1 (C-8''), 123.5 (C-4, C-7), 111.0 (C‑5''), 110.8 (C-7''), 63.8 (O-CH_2_), 60.2 (C-2''), 51.8 (C-3''), 42.3 (C-1'), 29.6 (C‑4''), 27.7 (2''-CH_3_), 25.9 (2''-CH_3_), 17.0 (4''-CH_3_), 15.0 (O-CH_2_-**CH_3_**). IR (ATR)
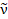
 [cm^-1^] = 2963, 2934, 1722, 1662, 1419, 1394, 1373, 1266, 1248, 1111, 717. MS (EI) *m/z* (%) = 406 (91) [M^•^]^+^, 218 (30), 204 (55), 190 (54), 160 (100). HRMS (EI) 406.1893 (calculated for C_24_H_26_N_2_O_4_^•+^: 406.1887). Purity (HPLC) > 99 % (λ = 210 nm).

**(*RS*)-2-[2-(2-Methylindolin-1-yl)-2-oxoethyl]isoindoline-1,3-dione (EVP-167)**

C_19_H_16_N_2_O_3_, M_r_ = 320.35

Prepared following general procedure 6a using (*RS*)-2-methylindoline (325 mg, 2.44 mmol) and *N*-phthalylglycyl chloride (523 mg, 2.34 mmol). FCC using hexanes/ethyl acetate 1:1 gave colorless crystals (76 %). mp: 170 °C; ^1^H-NMR (400 MHz, tetrachloroethane-*d_2_*, 70 °C) δ (ppm) = 8.11 – 7.75 (m, 1H, 7''-H), 7.90 – 7.82 (m, 2H, 4-H, 7-H), 7.75 – 7.69 (m, 2H, 5-H, 6-H), 7.25 – 7.14 (m, 2H, 4''-H, 6''-H), 7.05 (td, *J_1_* = 7.5 Hz, *J_2_* = 1.0 Hz, 1H, 5''-H), 4.77 – 4.62 (m, 2H, 1'-H, 2''-H), 4.56 (d, *J* = 16.1 Hz, 1H, 1'-H), 3.45 (dd, *J_1_* = 15.8 Hz, *J_2_* = 8.8 Hz, 1H, 3''-H), 2.68 (d, *J* = 15.8 Hz, 1H, 3''-H), 1.39 (d, *J* = 6.4 Hz, 3H, 2''-CH_3_). ^13^C-NMR (101 MHz, tetrachloroethane-*d_2_*, 70 °C) δ (ppm) = 167.8 (C-1, C-3), 163.3 (C-2'), 140.7 (C-7a''), 134.1 (C-5, C-6), 132.2 (C‑3a, C‑7a), 130.6 (C-3a''), 127.5 (C-6''), 125.4 (C-4''), 124.4 (C-5''), 123.5 (C-4, C‑7), 117.2 (C-7''), 55.5 (C-2''), 40.3 (C-1'), 36.5 (C-3''), 21.4 (2''-CH_3_). IR (ATR)
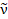
 [cm^-1^] = 3101, 3066, 2968, 1727, 1666, 1484, 1425, 1393, 1380, 1344, 1324, 1281, 951, 769. MS (EI) *m/z* (%) = 320 (48) [M^•^]^+^, 160 (100), 133 (54), 118 (41). HRMS (EI) 320.1157 (calculated for C_19_H_16_N_2_O_3_^•+^: 320.1155). Purity (HPLC) > 99 % (λ = 210 nm), > 99 % (λ = 254 nm).

**2-[2-((4a*RS*,8a*SR*)-Octahydroquinolin-1(2*H*)-yl)-2-oxoethyl]isoindoline-1,3-dione (EVP-168)**

C_19_H_22_N_2_O_3_, M_r_ = 326.40

Prepared following general procedure 6a using (4a*RS*,8a*SR*)-decahydroquinoline (606 mg, 4.35 mmol) and *N*-phthalylglycyl chloride (501 mg, 2.24 mmol). FCC using hexanes/ethyl acetate 1:1 gave a colorless solid (60 %). mp: 151 °C; ^1^H-NMR (500 MHz, CDCl_3_) δ (ppm) = 7.88 – 7.83 (m, 2H, 4-H, 7-H), 7.73 – 7.68 (m, 2H, 5-H, 6-H), 4.50 (d, *J* = 16.0 Hz, 1H, 1'-H), 4.42 (d, *J* = 16.0 Hz, 1H, 1'-H), 3.70 – 3.51 (m, 1H, 2''-H), 3.37 – 3.24 (m, 2H, 2''-H, 8a''-H), 2.17 – 2.11 (m, 1H, 8''-H), 1.97 – 1.87 (m, 1H, 3''-H), 1.78 – 1.63 (m, 5H, 3''-H, 4''-H, 5''-H, 6''-H, 7''-H), 1.63 – 1.55 (m, 1H, 4a''-H), 1.43 – 1.24 (m, 3H, 6''‑H, 7''‑H, 8''‑H), 1.24 – 1.16 (m, 1H, 4''-H), 1.11 – 1.01 (m, 1H, 5''-H). ^13^C-NMR (126 MHz, CDCl_3_) δ (ppm) = 168.3 (C-1, C-3), 164.7 (C-2'), 134.0 (C-5, C-6), 132.5 (C-3a, C-7a), 123.6 (C-4, C-7), 62.4 (C-8a''), 39.8 (C-1'), 39.0 (C-2''), 38.2 (C-4a''), 33.1 (C-5''), 30.9 (C‑8''), 26.3 (C-4''), 26.2 (C-6''), 25.6 (C-7''), 23.1 (C-3''). IR (ATR)
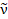
 [cm^-1^] = 2938, 2852, 1717, 1644, 1448, 1420, 1394, 1108, 959, 713. MS (EI) *m/z* (%) = 326 (9) [M^•^]^+^, 163 (100), 158 (32), 137 (19), 96 (19). HRMS (EI) 326.1625 (calculated for C_19_H_22_N_2_O_3_^•+^: 326.1625). Purity (HPLC) > 99 % (λ = 210 nm), > 99 % (λ = 254 nm).

**(*RS*)-3-Ethyl-3-methyl-1-[2-oxo-2-((*SR*)-2,2,4-trimethyl-3,4-dihydroquinolin-1(2*H*)-yl)ethyl]pyrrolidine-2,5-dione** and **(*RS*)-3-Ethyl-3-methyl-1-[2-oxo-2-((*RS*)-2,2,4-trimethyl-3,4-dihydroquinolin-1(2*H*)-yl)ethyl]pyrrolidine-2,5-dione (EVP-172)** (racemic mixture of diastereomers)

C_21_H_28_N_2_O_3_, M_r_ = 356.47

A mixture of (*RS*)-2-chloro-1-(2,2,4-trimethyl-3,4-dihydroquinolin-1(2*H*)-yl)ethan-1-one **(EVP-149)** (162 mg, 0.643 mmol), (*RS*)-ethosuximide (141 mg, 0.997 mmol), potassium carbonate (161 mg, 1.16 mmol), 3.0 g molecular sieves 4 Å and sodium iodide (60 mg, 0.40 mmol) was disperged in dry DMSO (5 mL) in a dry flask under nitrogen atmosphere. The resulting suspension was heated at 110 °C for 15 h. After cooling to room temperature the molecular sieves was removed by filtration and washed with ethyl acetate. The combined organic layers were washed with water and brine, dried over sodium sulfate, filtered and concentrated. FCC using hexanes/ethyl acetate 4:1 gave a colorless oil (76 %); ^1^H-NMR (400 MHz, CDCl_3_, mixture of the diastereomers, ratio 1:1) δ (ppm) = 7.22 – 7.11 (m, 2x 4H, 5''-H, 6''-H, 7''-H, 8''-H), 4.59 (d, *J* = 15.7 Hz, 1H, 1'-H), 4.58 (d, *J* = 15.7 Hz, 1H, 1'-H), 3.78 (d, *J* = 15.7 Hz, 1H, 1'-H), 3.77 (d, *J* = 15.7 Hz, 1H, 1'-H), 2.84 (dqd, *J_1_* = 13.3 Hz, *J_2_* = 6.8 Hz, *J_3_* = 2.7 Hz, 2x 1H, 4''-H), 2.63 (d, *J* = 18.1 Hz, 1H, 4-H), 2.62 (d, *J* = 18.2 Hz, 1H, 4-H), 2.41 (d, *J* = 18.1 Hz, 1H, 4-H), 2.39 (d, *J* = 18.2 Hz, 1H, 4-H), 1.82 (dd, *J* = 12.8, 2.7 Hz, 2x 1H, 3''-H), 1.77 – 1.66 (m, 2x 1H, 3-CH_2_), 1.64 (s, 3H, 2''-CH_3_), 1.63 (s, 3H, 2''-CH_3_), 1.62 – 1.54 (m, 2x 1H, 3-CH_2_), 1.43 (s, 2x 3H, 2''-CH_3_), 1.31 (d, *J* = 6.8 Hz, 2x 3H, 4''-CH_3_), 1.27 (s, 3H, 3-CH_3_), 1.27 (s, 3H, 3-CH_3_), 1.20 (dd, *J_1_* = 13.3 Hz, *J_2_* = 12.8 Hz, 2x 1H, 3''-H), 0.87 (t, *J* = 7.5 Hz, 3H, CH_2_-**CH_3_**), 0.87 (t, *J* = 7.5 Hz, 3H, CH_2_-**CH_3_**). ^13^C-NMR (101 MHz, CDCl_3_, mixture of the diastereomers ratio 1:1) δ (ppm) = 182.5 (C-2), 182.5 (C-2), 175.8 (C-5), 175.7 (C-5), 166.6 (C-2'), 166.6 (C-2'), 141.6 (C-4a''), 141.6 (C-4a''), 137.5 (C-8a''), 137.5 (C-8a''), 126.4 (C-7''), 126.3 (C-7''), 125.7 (C-6''), 125.7 (C-6''), 125.1 (C-8''), 125.1 (C-8''), 123.5 (C-5''), 123.4 (C-5''), 60.1 (C-2''), 60.1 (C-2''), 51.8 (C-3''), 51.8 (C-3''), 44.3 (C-3), 44.3 (C-3), 42.8 (C-1'), 42.8 (C-1'), 40.5 (C-4), 40.5 (C-4), 31.0 (3-CH_2_), 30.9 (3-CH_2_), 29.2 (C-4''), 29.2 (C-4''), 27.8 (2''-CH_3_), 27.8 (2''-CH_3_), 25.6 (2''-CH_3_), 25.6 (2''-CH_3_), 23.9 (3-CH_3_), 23.8 (3-CH_3_), 16.9 (4''-CH_3_), 16.9 (4''-CH_3_), 8.7 (CH_2_-**CH_3_**), 8.7 (CH_2_-**CH_3_**). IR (ATR)
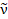
 [cm^-1^] = 3031, 3016, 2965, 2929, 2875, 1712, 1676, 1490, 1454, 1418, 1373, 1308, 1208, 1146, 756. MS (EI) *m/z* (%) = 356 (62) [M^•^]^+^, 215 (20), 175 (25), 174 (24), 161 (100), 155 (48), 148 (56). HRMS (EI) 356.2102 (calculated for C_21_H_28_N_2_O_3_: 356.2100). Purity (HPLC) 97 % (λ = 210 nm), 96 % (λ = 254 nm).

**(*RS*)-2-[2-Oxo-2-(2,2,4-trimethyl-3,4-dihydroquinolin-1(2*H*)-yl)ethyl]isoindoline-1-one (EVP-173)**

C_22_H_24_N_2_O_2_, M_r_ = 348.45

A mixture of (*RS*)-2-chloro-1-(2,2,4-trimethyl-3,4-dihydroquinolin-1(2*H*)-yl)ethan-1-one **(EVP-149)** (122 mg, 0.485 mmol), isoindoline-1-one (107 mg, 0.801 mmol), 3.0 g molecular sieves 4 Å and sodium iodide (106 mg, 0.707 mmol) was disperged in dry THF (3 mL) in a dry flask under nitrogen atmosphere. Under stirring at ambient temperature LiHMDS (1 M in toluene, 0.5 mL, 0.5 mmol) was added slowly and the resulting suspension was heated at 70 °C for 15 h. After cooling to room temperature the mixture was transferred to FCC using hexanes/ethyl acetate 4:1 to give yellow crystals (58 %). mp: 113 °C; ^1^H-NMR (500 MHz, CDCl_3_) δ (ppm) = 7.83 (dd, *J_1_* = 7.5 Hz, *J_2_* = 1.1 Hz, 1H, 7-H), 7.51 (ddd, *J_1_* = 7.5 Hz, *J_2_* = 7.4 Hz, *J_3_* = 1.2 Hz, 1H, 5-H), 7.46 – 7.38 (m, 2H, 4-H, 6-H), 7.25 – 7.17 (m, 4H, 5''-H, 6''-H, 7''‑H, 8''‑H), 4.77 (d, *J* = 16.2 Hz, 1H, 1'-H), 4.50 (s, 2H, 3-H), 3.96 (d, *J* = 16.2 Hz, 1H, 1'-H), 2.85 (dqd, *J_1_* = 13.4 Hz, *J_2_* = 6.8 Hz, *J_3_* = 2.6 Hz, 1H, 4''-H), 1.85 (dd, *J_1_* = 12.8 Hz, *J_2_* = 2.7 Hz, 1H, 3''-H), 1.70 (s, 3H, 2''-CH_3_), 1.48 (s, 3H, 2''-CH_3_), 1.34 (d, *J* = 6.8 Hz, 3H, 4''-CH_3_), 1.24 (dd, *J_1_* = 13.3 Hz, *J_2_* = 12.6 Hz, 1H, 3''-H). ^13^C-NMR (126 MHz, CDCl_3_) δ (ppm) = 169.6 (C-2'), 169.0 (C-1), 142.0 (C-3a), 141.4 (C-4a''), 137.8 (C-8a''), 132.3 (C-7a), 131.5 (C-5), 128.0 (C-6), 126.5 (C-7''), 125.8 (C-6''), 125.2 (C-8''), 123.9 (C-7), 123.4 (C-5''), 122.8 (C-4), 60.0 (C-2''), 51.9 (C-3''), 51.1 (C-3), 47.2 (C-1'), 29.3 (C-4''), 27.9 (2''-CH_3_), 25.9 (2''-CH_3_), 17.0 (4''-CH_3_). IR (ATR)
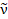
 [cm^-1^] = 3081, 3019, 2968, 2929, 1699, 1659, 1488, 1363, 1299, 1244, 766, 736. MS (EI) *m/z* (%) = 348 (17) [M^•^]^+^, 175 (27), 146 (100). HRMS (EI) 348.1854 (calculated for C_22_H_24_N_2_O_2_: 348.1838). Purity (HPLC) 97 % (λ = 210 nm), 99 % (λ = 254 nm).

**(*RS*)-2-Bromo-1-(2,2,4-trimethyl-3,4-dihydroquinolin-1(2*H*)-yl)ethan-1-one (EVP-175)**

C_14_H_18_BrNO, M_r_ = 296.21

Potassium carbonate (4.73 g, 34.2 mmol) was added to a solution of (*RS*)-2,2,4-trimethyl-1,2,3,4-tetrahydroquinoline (4.01 g, 22.9 mmol) in acetone (35 mL) and the mixture stirred in an ice bath. After 15 min bromoacetyl bromide (5.5 mL, 63 mmol) was added dropwise over 1 hour and the mixture was stirred for another 15 hours while slowly warming to room temperature. Then the mixture was poured into water (50 mL) and extracted with ethyl acetate (3 × 50 mL). The combined organic layers were washed with brine, dried over sodium sulfate, filtered and concentrated. FCC using hexanes/ethyl acetate 9:1 gave a yellow oil (52 %); ^1^H-NMR (400 MHz, CDCl_3_) δ (ppm) = 7.24 – 7.14 (m, 3H, 5'-H, 6'-H, 7'-H), 7.02 – 6.94 (m, 1H, 8'-H), 4.03 (d, *J* = 10.9 Hz, 1H, 2-H), 3.82 (d, *J* = 10.9 Hz, 1H, 2-H), 2.80 (dqd, *J_1_* = 13.4 Hz, *J_2_* = 6.8 Hz, *J_3_* = 2.6 Hz, 1H, 4'-H), 1.88 (dd, *J_1_* = 12.8 Hz, *J_2_* = 2.7 Hz, 1H, 3'-H), 1.68 (s, 3H, 2'-CH_3_), 1.49 (s, 3H, 2'-CH_3_), 1.34 (d, *J* = 6.8 Hz, 3H, 4'-CH_3_), 1.24 (t, *J* = 12.5 Hz, 1H, 3'-H). ^13^C-NMR (126 MHz, CDCl_3_) δ (ppm) = 168.3 (C-1), 141.2 (C-4a'), 138.5 (C-8a'), 126.4 (C-7'), 125.9 (C-6'), 124.4 (C‑8'), 123.4 (C-5'), 59.8 (C-2'), 51.8 (C-3'), 30.9 (C-2), 29.1 (C-4'), 27.8 (2'-CH_3_), 25.1 (2'-CH_3_), 16.9 (4'-CH_3_). IR (ATR)
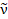
 [cm^-1^] = 3055, 3002, 2989, 2965, 2934, 2876, 1654, 1490, 1342, 1208, 1196, 768, 650. MS (EI) *m/z* (%) = 297 (50) [(^81^Br) M^•^]^+^, 295 (35) [(^79^Br) M^•^]^+^, 282 (32), 280 (32), 160 (100). HRMS (EI) 295.0577 (calculated for C_14_H_18_BrNO: 295.0572). Purity (HPLC) > 99 % (λ = 210 nm), > 99 % (λ = 254 nm).

**(3a*R*,4*S*,7*R*,7a*S*)-2-[2-Oxo-2-((*RS*)-2,2,4-trimethyl-3,4-dihydroquinolin-1(2*H*)-yl)ethyl]-3a,4,7,7a-tetrahydro-1*H*-4,7-epoxyisoindole-1,3(2*H*)-dione (EVP-177)**

C_22_H_24_N_2_O_4_, M_r_ = 380.44

Prepared following general procedure 7 using (*RS*)-2-bromo-1-(2,2,4-trimethyl-3,4-dihydroquinolin-1(2*H*)-yl)ethan-1-one **(EVP-175)** (600 mg, 2.03 mmol) and *exo*-3a,4,7,7a-tetrahydro-1*H*-4,7-epoxyisoindole-1,3(2*H*)-dione (415 mg, 2.51 mmol). FCC using hexanes/ethyl acetate 1:1 gave a colorless solid (55 %). mp: 156 °C; ^1^H-NMR (500 MHz, CDCl_3_) δ (ppm) = 7.23 – 7.12 (m, 4H, 5''-H, 6''-H, 7''-H, 8''-H), 6.51 – 6.45 (m, 2H, 5-H, 6-H), 5.23 (d, *J* = 1.4 Hz, 1H, 4-H, 7-H), 5.19 (d, *J* = 1.4 Hz, 1H, 4-H, 7-H), 4.59 (d, *J* = 15.9 Hz, 1H, 1'-H), 3.82 (d, *J* = 15.9 Hz, 1H, 1'-H), 2.91 (d, *J* = 6.5 Hz, 1H, 3a-H, 7a-H), 2.85 (d, *J* = 6.5 Hz, 1H, 3a-H, 7a-H), 2.89 - 2.81 (m, 1H, 4''-H), 1.84 (dd, *J_1_* = 12.8 Hz, *J_2_* = 2.7 Hz, 1H, 3''‑H), 1.66 (s, 3H, 2''-CH_3_), 1.45 (s, 3H, 2''-CH_3_), 1.33 (d, *J* = 6.8 Hz, 3H, 4''-CH_3_), 1.22 (dd, *J_1_*= 12.7 Hz, *J_2_* = 12.4 Hz, 1H, 3''-H). ^13^C-NMR (126 MHz, CDCl_3_) δ (ppm) = 175.8 (C-1, C-3), 175.6 (C-1, C-3), 166.4 (C-2'), 141.7 (C-4a''), 137.5 (C-8a''), 136.7 (C-5, C-6), 136.6 (C-5, C-6), 126.5 (C-7''), 125.9 (C-6''), 125.1 (C-8''), 123.5 (C-5''), 80.9 (C-4, C-7), 80.9 (C-4, C-7), 60.3 (C-2''), 51.8 (C-3''), 47.9/47.7 (C-3a, C-7a), 43.1 (C-1'), 29.3 (C-4''), 27.9 (2''-CH_3_), 25.7 (2''-CH_3_), 17.0 (4''-CH_3_). IR (ATR)
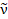
 [cm^-1^] = 3024, 2981, 2944, 1706, 1668, 1424, 1312, 1180, 1016, 882. MS (EI) *m/z* (%) = 380 (8) [M^•^]^+^, 312 (52), 270 (33), 202 (26), 160 (89), 146 (100). HRMS (EI) 380.1736 (calculated for C_22_H_24_N_2_O_4_: 380.1736). Purity (HPLC) 99 % (λ = 210 nm), > 99 % (λ = 254 nm).

**(3a*R*,7a*S*)-2-[2-Oxo-2-((*RS*)-2,2,4-trimethyl-3,4-dihydroquinolin-1(2*H*)-yl)ethyl]-3a,4,7,7a-tetrahydro-1*H*-isoindole-1,3(2*H*)-dione (EVP-179)**

C_22_H_26_N_2_O_3_, M_r_ = 366.46

Prepared following general procedure 7 using (*RS*)-2-bromo-1-(2,2,4-trimethyl-3,4-dihydroquinolin-1(2*H*)-yl)ethan-1-one **(EVP-175)** (427 mg, 1.44 mmol) and 3a,4,7,7a-tetrahydro-1*H*-isoindole-1,3(2*H*)-dione (272 mg, 1.80 mmol). FCC using hexanes/ethyl acetate 1:1 gave a beige solid (68 %). mp: 52 °C; ^1^H-NMR (500 MHz, CDCl_3_) δ (ppm) = 7.21 – 7.18 (m, 1H, 5''-H), 7.18 – 7.14 (m, 2H, 6''-H, 7''-H), 7.14 – 7.10 (m, 1H, 8''-H), 5.87 – 5.78 (m, 2H, 5-H, 6-H), 4.55 (d, *J* = 15.8 Hz, 1H, 1'-H), 3.78 (d, *J* = 15.7 Hz, 1H, 1'-H), 3.13 – 3.02 (m, 2H, 3a-H, 7a-H), 2.83 (dqd, *J_1_* = 13.5 Hz, *J_2_* = 6.8 Hz, *J_3_* = 2.5 Hz, 1H, 4''-H), 2.55 – 2.46 (m, 2H, 4-H, 7-H), 2.26 – 2.17 (m, 2H, 4-H, 7-H), 1.82 (dd, *J_1_* = 12.8 Hz, *J_2_* = 2.7 Hz, 1H, 3''-H), 1.63 (s, 3H, 2''-CH_3_), 1.43 (s, 3H, 2''-CH_3_), 1.31 (d, *J* = 6.8 Hz, 3H, 4''-CH_3_), 1.19 (dd, *J_1_* = 13,4 Hz, *J_2_*  = 12.8 Hz, 1H, 3''-H). ^13^C-NMR (126 MHz, CDCl_3_) δ (ppm) = 179.7 (C-1, C-3), 179.7 (C-1, C-3), 166.6 (C-2'), 141.7 (C-4a''), 137.6 (C-8a''), 127.7 (C-5, C-6), 126.5 (C-7''), 125.8 (C-6''), 125.1 (C-8''), 123.5 (C-5''), 60.2 (C-2''), 51.8 (C‑3''), 43.1 (C-1'), 39.3 (C-3a, C-7a), 39.2 (C-3a, C-7a), 29.3 (C-4''), 27.9 (2''-CH_3_), 25.7 (2''-CH_3_), 23.5 (C-4, C-7), 23.5 (C-4, C-7), 17.0 (4''-CH_3_). IR (ATR)
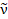
 [cm^-1^] = 3027, 2955, 2933, 2874, 1698, 1667, 1419, 1309, 1246, 1173, 762, 747. MS (EI) *m/z* (%) = 366 (67) [M^•^]^+^, 164 (50), 160 (100), 146 (60). HRMS (EI) 366.1939 (calculated for C_22_H_26_N_2_O_3_: 366.1944). Purity (HPLC) 96 % (λ = 210 nm), 98 % (λ = 254 nm).

**(3a*RS*,4*RS*,7*SR*,7a*SR*)-5-Bromo-3a,4,7,7a-tetrahydro-1*H*-4,7-epoxyisoindole-1,3(2*H*)-dione (EVP-183)**

C_8_H_6_BrNO_3_, M_r_ = 244.04

A solution of maleimide (320 mg, 3.29 mmol) and 3-bromofurane (626 mg, 4.26 mmol) in toluene (4mL) was heated to 105 °C for 36 h. Cooling down, a brown precipitate formed that was collected by filtration and washed with diethyl ether to give a light brown solid (90 %). mp: 206 °C; ^1^H-NMR (500 MHz, DMSO-*d*_6_) δ (ppm) = 11.29 (s, 1H, NH), 6.76 (d, *J* = 2.0 Hz, 1H, 6-H), 5.18 (dd, *J_1_* = 2.1 Hz, *J_2_* = 0.8 Hz, 1H, 7-H), 5.06 (d, *J* = 0.8 Hz, 1H, 4-H), 3.07 (d, *J* = 6.5 Hz, 1H, 7a-H), 3.01 (d, *J* = 6.5 Hz, 1H, 3a-H). ^13^C-NMR (126 MHz, DMSO-*d*_6_) δ (ppm) = 177.0 (C-1), 176.8 (C-3), 136.2 (C-6), 125.7 (C-5), 84.2 (C-4), 82.5 (C-7), 49.4 (C-7a), 47.9 (C-3a). IR (ATR)
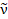
 [cm^-1^] = 3160, 3075, 3014, 2944, 2804, 1770, 1707, 1583, 1356, 1254, 1198, 833. MS (EI) *m/z* (%) = 245 (12) [(^81^Br)M^•^]^+^, 243 (14) [(^79^Br)M^•^]^+^, 148 (96), 146 (100). HRMS (EI) 242.9554 (calculated for C_8_H_6_^79^BrNO_3_: 242.9531). Purity (HPLC) 97 % (λ = 210 nm), > 99 % (λ = 254 nm).

**(3a*R*,4*S*,7*R*,7a*S*)-2-[2-Oxo-2-((*RS*)-2,2,4-trimethyl-3,4-dihydroquinolin-1(2*H*)-yl)ethyl]-3a,4,7,7a-tetrahydro-1*H*-4,7-methanoisoindole-1,3(2*H*)-dione (EVP-184)**

C_23_H_26_N_2_O_3_, M_r_ = 378.47

Prepared following general procedure 7 using (*RS*)-2-bromo-1-(2,2,4-trimethyl-3,4-dihydroquinolin-1(2*H*)-yl)ethan-1-one **(EVP-175)** (428 mg, 1.44 mmol) and *endo*-3a,4,7,7a-tetrahydro-1*H*-4,7-methanoisoindole-1,3(2*H*)-dione (376 mg, 2.30 mmol). FCC using hexanes/ethyl acetate 4:1 gave a beige solid (57 %). mp: 130 °C; ^1^H-NMR (500 MHz, CDCl_3_) δ (ppm) = 7.15 – 7.10 (m, 1H, 5''-H), 7.10 – 7.04 (m, 2H, 6''-H, 7''-H), 7.02 – 6.97 (m, 1H, 8''-H), 5.97 – 5.92 (m, 2H, 5-H, 6-H), 4.32 (d, *J* = 15.6 Hz, 1H, 1'-H), 3.58 (d, *J* = 15.6 Hz, 1H, 1'-H), 3.28 – 3.22 (m, 2H, 4-H, 7-H), 3.22 – 3.14 (m, 2H, 3a-H, 7a-H), 2.75 (dqd, *J_1_* = 13.5 Hz, *J_2_* = 6.8 Hz, *J_3_* = 2.6 Hz, 1H, 4''-H), 1.74 (dd, *J_1_* = 12.6 Hz, *J_2_* = 2.6 Hz, 1H, 3''-H), 1.60 (dt, *J_1_* = 8.7 Hz, *J_2_* = 1.7 Hz, 1H, 8-H), 1.55 (s, 3H, 2''-CH_3_), 1.44 (dt, *J_1_* = 8.8 Hz, J_2_ = 1.5 Hz, 1H, 8-H), 1.35 (s, 3H, 2''-CH_3_), 1.24 (d, *J* = 6.9 Hz, 3H, 4''-CH_3_), 1.11 (dd, *J_1_* = 13.4 Hz, *J_2_* = 12.5 Hz, 1H, 3''-H). ^13^C-NMR (126 MHz, CDCl_3_) δ (ppm) = 176.8 (C-1, C-3), 176.7 (C-1, C-3), 166.4 (C-2'), 141.2 (C-4a''), 137.3 (C-8a''), 134.3 (C-6), 134.2 (C-5), 126.1 (C-7''), 125.4 (C-6''), 124.7 (C-8''), 123.2 (C-5''), 59.7 (C-2''), 52.0 (C-8), 51.5 (C-3''), 45.9 (C-3a, C-7a), 45.8 (C-3a, C-7a), 44.6 (C-4, C-7), 44.5 (C-4, C-7), 42.2 (C-1'), 28.9 (C-4''), 27.6 (2''-CH_3_), 25.3 (2''-CH_3_), 16.7 (4''-CH_3_). IR (ATR)
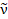
 [cm^-1^] = 3004, 2960, 2948, 2875, 1707, 1657, 1395, 1311, 1175, 746, 719. MS (EI) *m/z* (%) = 378 (20) [M^•^]^+^, 252 (100), 250 (60), 235 (60), 233 (37), 160 (26). HRMS (EI) 378.1953 (calculated for C_23_H_26_N_2_O_3_: 378.1944). Purity (HPLC) > 99 % (λ = 210 nm), > 99 % (λ = 254 nm).

**(3a*RS*,4*RS*,7*SR*,7a*SR*)-5-Bromo-2-[2-oxo-2-((*SR*)-2,2,4-trimethyl-3,4-dihydroquinolin-1(2*H*)-yl)ethyl]-3a,4,7,7a-tetrahydro-1*H*-4,7-epoxyisoindole-1,3(2*H*)-dione** and **(3a*RS*,4*RS*,7*SR*,7a*SR*)-5-bromo-2-[2-oxo-2-((*RS*)-2,2,4-trimethyl-3,4-dihydroquinolin-1(2*H*)-yl)ethyl]-3a,4,7,7a-tetrahydro-1*H*-4,7-epoxyisoindole-1,3(2*H*)-dione (EVP-185)** (racemic mixture of diastereomers)

C_22_H_23_BrN_2_O_4_, M_r_ = 459.34

Prepared following general procedure 7 using (*RS*)-2-bromo-1-(2,2,4-trimethyl-3,4-dihydroquinolin-1(2*H*)-yl)ethan-1-one **(EVP-175)** (302 mg, 1.02 mmol) and (3a*RS*,4*RS*,7*SR*,7a*SR*)-5-bromo-3a,4,7,7a-tetrahydro-1*H*-4,7-epoxyisoindole-1,3(2*H*)-dione **(EVP-183)** (394 mg, 1.61 mmol). FCC using hexanes/ethyl acetate 4:1 gave colorless crystals (59 %). mp: 112 °C; ^1^H-NMR (400 MHz, CDCl_3_, mixture of diastereomers, ratio 1:1) δ (ppm) = 7.23 – 7.15 (m, 2x 3H, 5''-H, 6''-H, 7''-H), 7.15 – 7.09 (m, 2x 1H, 8''-H), 6.50 (d, *J* = 1.9 Hz, 1H, 6-H), 6.49 (d, *J* = 1.9 Hz, 1H, 6-H), 5.21 (dd, *J_1_* = 1.9 Hz, *J_2_* = 0.8 Hz, 1H, 7-H), 5.17 (dd, *J_1_* = 1.9 Hz, *J_2_* = 0.8 Hz, 1H, 7-H), 5.05 (d, *J* = 0.8 Hz, 1H, 4-H), 5.00 (d, *J* = 0.8 Hz, 1H, 4-H), 4.59 (d, *J* = 15.8 Hz, 1H, 1'-H), 4.58 (d, *J* = 15.8 Hz, 1H, 1'-H), 3.81 (d, *J* = 15.8 Hz, 1H, 1'-H), 3.81 (d, *J* = 15.8 Hz, 1H, 1'-H), 3.09 – 2.96 (m, 2x 2H, 3a-H, 7a-H), 2.89 – 2.78 (m, 2x 1H, 4''-H), 1.84 (dd, *J_1_* = 12.9 Hz, *J_2_* = 2.7 Hz, 2x 1H, 3''-H), 1.65 (s, 2x 3H, 2''-CH_3_), 1.44 (s, 2x 3H, 2''-CH_3_), 1.33 (d, *J* = 6.8 Hz, 2x 3H, 4''-CH_3_), 1.21 (dd, *J_1_* = 12.8 Hz, *J_2_* = 12.4 Hz, 2x 1H, 3''-H). ^13^C-NMR (101 MHz, CDCl_3_, mixture of diastereomers, ratio 1:1) δ (ppm) = 174.7 (C-1, C-3), 174.5 (C-1, C-3), 166.2 (C-2'), 166.2 (C-2'), 141.7 (C-4a''), 141.7 (C-4a''), 137.4 (C-8a''), 135.0 (C-6), 135.0 (C-6), 127.4 (C-5), 127.4 (C-5), 126.5 (C-7''), 126.5 (C-7''), 125.9 (C-6''), 125.9 (C-6''), 125.0 (C-8''), 125.0 (C-8''), 123.6 (C-5''), 123.6 (C-5''), 85.2 (C-4), 85.1 (C-4), 82.8 (C-7), 82.8 (C-7), 60.3 (C-2''), 60.3 (C-2''), 51.8 (C-3''), 51.8 (C-3''), 48.9 (C-7a), 48.7 (C-7a), 47.1 (C-3a), 46.9 (C-3a), 43.3 (C-1'), 43.3 (C-1'), 29.3 (C-4''), 29.3 (C-4''), 27.9 (2''-CH_3_), 27.9 (2''-CH_3_), 25.7 (2''-CH_3_), 25.7 (2''-CH_3_), 17.0 (4''-CH_3_), 17.0 (4''-CH_3_). IR (ATR)
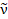
 [cm^-1^] = 3016, 2998, 2980, 2900, 1706, 1671, 1581, 1490, 1420, 1399, 1332, 1308, 1177. MS (EI) *m/z* (%) = 460 (12) [(^81^Br)M^•^]^+^, 458 (12) [(^79^Br)M^•^]^+^, 312 (68), 252 (54), 160 (89), 146 (100). HRMS (EI) 458.0830 (calculated for C_22_H_23_^79^BrN_2_O_4_: 458.0841). Purity (HPLC) > 99 % (λ = 210 nm), > 99 % (λ = 254 nm).

**(*SR*)-1-[2-Oxo-2-((*SR*)-2,2,4-trimethyl-3,4-dihydroquinolin-1(2*H*)-yl)ethyl]-3-(1*H*-pyrrol-2-yl)pyrrolidine-2,5-dione (EVP-190)** and **(*SR*)-1-[2-oxo-2-((*RS*)-2,2,4-trimethyl-3,4-dihydroquinolin-1(2*H*)-yl)ethyl]-3-(1*H*-pyrrol-2-yl)pyrrolidine-2,5-dione (EVP-190)** (racemic mixture of diastereomers)

C_22_H_25_N_3_O_3_, M_r_ = 379.46

Prepared following general procedure 7 using (*RS*)-2-bromo-1-(2,2,4-trimethyl-3,4-dihydroquinolin-1(2*H*)-yl)ethan-1-one **(EVP-175)** (204 mg, 0.689 mmol) and (*RS*)-3-(1*H*-pyrrol-2-yl)pyrrolidine-2,5-dione (100 mg, 0.609 mmol). FCC using hexanes/ethyl acetate 2:1 gave a colorless oil (22 %); ^1^H-NMR (500 MHz, CDCl_3_, mixture of diastereomers, ratio 1:1) δ (ppm) = 9.35 (s, 2 x 1H, NH), 7.25 – 7.15 (m, 2 x 4H, 5''-H, 6''-H, 7''-H, 8''-H), 6.83 – 6.74 (m, 2 x 1H, 5'''-H), 6.16 – 6.11 (m, 2 x 1H, 4'''-H), 6.05 – 6.00 (m, 2 x 1H, 3'''-H), 4.69 (d, *J* = 15.9 Hz, 1H, 1'-H), 4.68 (d, *J* = 15.9 Hz, 1H, 1'-H), 4.17 – 4.09 (m, 2 x 1H, 3-H), 3.89 (d, *J* = 15.9 Hz, 1H, 1'-H), 3.88 (d, *J* = 15.9 Hz, 1H, 1'-H), 3.17 (dd, *J_1_* = 18.2 Hz, *J_2_* = 7.0 Hz, 1H, 4-H), 3.16 (dd, *J_1_* = 18.1 Hz, *J_2_* = 7.1 Hz, 1H, 4-H), 2.90 (dd, *J_1_* = 18.0 Hz, *J_2_* = 4.5 Hz, 1H, 4-H), 2.83 (dd, *J_1_* = 18.1 Hz, *J_2_* = 4.6 Hz, 1H, 4-H), 2.85 – 2.79 (m, 2 x 1H, 4''-H), 1.85 (dd, *J_1_* = 12.9 Hz, *J_2_* = 2.8 Hz, 2 x 1H, 3''-H), 1.69 (s, 3H, 2''-CH_3_), 1.66 (s, 3H, 2''-CH_3_), 1.47 (s, 3H, 2''-CH_3_), 1.46 (s, 3H, 2''-CH_3_), 1.34 (d, *J* = 6.8 Hz, 3H, 4''-CH_3_), 1.33 (d, *J* = 6.8 Hz, 3H, 4''-CH_3_), 1.25 – 1.19 (m, 2 x 1H, 3''-H). ^13^C-NMR (126 MHz, CDCl_3_, mixture of diastereomers, ratio 1:1) δ (ppm) = 177.1 (C-2), 177.1 (C-2), 175.5 (C-5), 175.5 (C-5), 166.6 (C-2'), 166.5 (C-2'), 141.7 (C-4a''), 141.7 (C-4a''), 137.1 (C-8a''), 137.1 (C-8a''), 126.5 (C-7''), 126.4 (C-7''), 126.2 (C-2'''), 126.2 (C-2'''), 126.1 (C-6''), 126.0 (C-6''), 125.1 (C-8''), 125.1 (C-8''), 123.5 (C-5''), 123.5 (C-5''), 118.8 (C-5'''), 118.8 (C-5'''), 108.3 (C-4'''), 108.2 (C-4'''), 106.4 (C-3'''), 106.3 (C-3'''), 60.5 (C-2''), 60.4 (C-2''), 51.7 (C-3''), 51.7 (C-3''), 43.1 (C-1'), 43.0 (C-1'), 39.2 (C-3), 39.2 (C-3), 35.5 (C-4), 35.3 (C-4), 29.2 (C-4''), 29.2 (C-4''), 27.8 (2''-CH_3_), 27.8 (2''-CH_3_), 25.7 (2''-CH_3_), 25.6 (2''-CH_3_), 16.9 (4''-CH_3_), 16.9 (4''-CH_3_). IR (ATR)
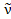
 [cm^-1^] = 3358, 3024, 3013, 2961, 2933, 1708, 1669, 1489, 1422, 1378, 1309, 1248, 1176, 760, 727. MS (EI) *m/z* (%) = 379 (25) [M^•^]^+^, 377 (25), 252 (100), 250 (85), 235 (60), 233 (40), 219 (50), 175 (50), 160 (60), 146 (40). HRMS (EI) 379.1870 (calculated for C_22_H_25_N_3_O_3_: 379.1896). Purity (HPLC) 96 % (λ = 210 nm), 96 % (λ = 254 nm).

**(*RS*)-2-[2-Oxo-2-(2,2,4-trimethyl-3,4-dihydroquinolin-1(2*H*)-yl)ethyl]-1*H*-benzo[*de*] isoquinoline-1,3(2*H*)-dione (EVP-191)**

C_26_H_24_N_2_O_3_, M_r_ = 412.49

Method A:
A mixture of (*RS*)-2-bromo-1-(2,2,4-trimethyl-3,4-dihydroquinolin-1(2*H*)-yl)ethan-1-one **(EVP-175)** (296 mg, 1.00 mmol), naphthalimide (201 mg, 1.02 mmol) and potassium carbonate (139 mg, 1.01 mmol) was disperged in dry DMSO (15 mL) in a dry flask under nitrogen atmosphere. The resulting suspension was refluxed for 3 hours. After cooling to room temperature the mixture was poured into water (50 mL) and extracted with ethyl acetate (3 × 50 mL). The combined organic layers were washed with water and brine, dried over sodium sulfate, filtered and concentrated. FCC using hexanes/ethyl acetate 4:1 gave a colorless solid (47 %).

Method B:
Prepared following general procedure 7 using (*RS*)-2-bromo-1-(2,2,4-trimethyl-3,4-dihydroquinolin-1(2*H*)-yl)ethan-1-one **(EVP-175)** (169 mg, 0.569 mmol) and naphthalimide (257 mg, 1.30 mmol). FCC using hexanes/ethyl acetate 4:1 gave a colorless solid (58 %).

mp: 191 °C; ^1^H-NMR (400 MHz, CDCl_3_) δ (ppm) = 8.58 (dd, *J_1_* = 7.3 Hz, *J_2_* = 1.2 Hz, 2H, 4-H, 9-H), 8.20 (dd, *J_1_* = 8.4 Hz, *J_2_* = 1.1 Hz, 2H, 6-H, 7-H), 7.73 (dd, *J_1_* = 8.3 Hz, *J_2_* = 7.2 Hz, 2H, 5-H, 8-H), 7.44 – 7.40 (m, 1H, 8''-H), 7.28 – 7.17 (m, 3H, 5''-H, 6''-H, 7''-H), 5.46 (d, *J* = 15.6 Hz, 1H, 1'-H), 4.47 (d, *J* = 15.5 Hz, 1H, 1'-H), 2.98 (dqd, *J_1_* = 13.4 Hz, *J_2_* = 6.7 Hz, *J_3_* = 2.6 Hz, 1H, 4''-H), 1.87 (dd, *J_1_* = 12.8 Hz, *J_2_* = 2.7 Hz, 1H, 3''-H), 1.71 (s, 3H, 2''-CH_3_), 1.48 (s, 3H, 2''-CH_3_), 1.37 (d, *J* = 6.7 Hz, 3H, 4''-CH_3_), 1.30 – 1.22 (dd, *J_1_* = 13.4 Hz, *J_2_* = 12.7 Hz, 1H, 3''-H). ^13^C-NMR (101 MHz, CDCl_3_) δ (ppm) = 167.9 (C-2'), 164.1 (C-1, C-3), 141.7 (C-4a''), 137.9 (C-8a''), 134.1 (C-6, C-7), 131.6 (C-6a), 131.4 (C-4, C-9), 128.4 (C-9b), 126.8 (C-5, C-8), 126.3 (C-7''), 125.5 (C-6''), 125.3 (C-8''), 123.3 (C-5''), 122.5 (C-3a, C-9a), 60.0 (C-2''), 52.0 (C-3''), 44.6 (C-1'), 29.3 (C-4''), 27.9 (2''-CH_3_), 25.8 (2''-CH_3_), 16.9 (4''-CH_3_). IR (ATR)
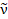
 [cm^-1^] = 3062, 3016, 2994, 2981, 2957, 2933, 1701, 1667, 1586, 1383, 1308, 1249, 1237, 777. MS (EI) *m/z* (%) = 412 (20) [M^•^]^+^, 270 (15), 238 (45), 210 (100), 175 (35). HRMS (EI) 412.1777 (calculated for C_26_H_24_N_2_O_3_: 412.1787). Purity (HPLC) > 99 % (λ = 210 nm), > 99 % (λ = 254 nm).

**(*RS*)-3,7-Dimethyl-1-[2-oxo-2-(2,2,4-trimethyl-3,4-dihydroquinolin-1(2*H*)-yl)ethyl]-3,7-dihydro-1*H*-purine-2,6-dione (EVP-192)**

C_21_H_25_N_5_O_3_, M_r_ = 395.46

A mixture of (*RS*)-2-bromo-1-(2,2,4-trimethyl-3,4-dihydroquinolin-1(2*H*)-yl)ethan-1-one **(EVP-175)** (305 mg, 1.03 mmol), theobromine (191 mg, 1.06 mmol), potassium carbonate (181 mg, 1.31 mmol) and tetrabutylammonium bromide (34 mg, 0.10 mmol) was disperged in dry DMF (7 mL) in a dry flask under nitrogen atmosphere. The resulting suspension was refluxed for 3 h. After cooling to room temperature the mixture was poured into water (50 mL) and extracted with chloroform (3 × 50 mL). The combined organic layers were washed with and brine, dried over sodium sulfate, filtered and concentrated. FCC using dichloromethane/methanol 19:1 gave a pale yellow solid (38 %). mp: 195 °C; ^1^H-NMR (500 MHz, CDCl_3_) δ (ppm) = 7.48 (s, 1H, 8-H), 7.33 (dd, *J_1_* = 7.8 Hz, *J_2_* = 1.7 Hz, 1H, 8''-H), 7.21 – 7.13 (m, 3H, 5''-H, 6''-H, 7''-H), 5.24 (d, *J* = 15.8 Hz, 1H, 1'-H), 4.25 (d, *J* = 15.7 Hz, 1H, 1'-H), 3.92 (s, 3H, 7-CH_3_), 3.52 (s, 3H, 3-CH_3_), 2.88 (dqd, *J_1_* = 12.3 Hz, *J_2_* = 6.9 Hz, *J_3_* = 2.7 Hz, 1H, 4''-H), 1.82 (dd, *J_1_* = 12.8 Hz, *J_2_* = 2.7 Hz, 1H, 3''-H), 1.66 (s, 3H, 2''-CH_3_), 1.43 (s, 3H, 2''-CH_3_), 1.31 (d, *J* = 6.9 Hz, 3H, 4''-CH_3_), 1.20 (dd, *J_1_* = 12.8 Hz, *J_2_* = 12.3 Hz, 2H, 3''-H). ^13^C-NMR (126 MHz, CDCl_3_) δ (ppm) = 168.2 (C-2'), 155.0 (C-6), 151.5 (C-2), 149.1 (C-4), 141.7 (C-4a''), 141.6 (C-8), 137.7 (C-8a''), 126.3 (C-7''), 125.6 (C-6''), 125.3 (C-8''), 123.3 (C-5''), 107.5 (C-5), 60.0 (C-2''), 52.0 (C-3''), 45.1 (C-1'), 33.6 (7-CH_3_), 29.8 (3-CH_3_), 29.2 (C-4''), 28.0 (2''-CH_3_), 25.9 (2''-CH_3_), 16.9 (4''-CH_3_). IR (ATR)
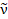
 [cm^-1^] = 3127, 3023, 2993, 2980, 2926, 2890, 2856, 1708, 1672, 1660, 1602, 1489, 1452, 1372, 1308, 1238, 752. MS (EI) *m/z* (%) = 395 (25) [M^•^]^+^, 221 (55), 193 (100), 175 (20). HRMS (EI) 395.1955 (calculated for C_21_H_25_N_5_O_3_: 395.1957). Purity (HPLC) 98 % (λ = 210 nm), 99 % (λ = 254 nm).

**(*RS*)-5,6-Dichloro-2-[2-oxo-2-(2,2,4-trimethyl-3,4-dihydroquinolin-1(2*H*)-yl)ethyl]isoindoline-1,3-dione (EVP-194)**

C_22_H_20_Cl_2_N_2_O_3_, M_r_ = 431.31

Prepared following general procedure 7 using (*RS*)-2-bromo-1-(2,2,4-trimethyl-3,4-dihydroquinolin-1(2*H*)-yl)ethan-1-one **(EVP-175)** (303 mg, 1.02 mmol) and 4,5-dichlorophthalimide (251 mg, 1.16 mmol). FCC using hexanes/ethyl acetate 9:1 gave colorless crystals (60 %). mp: 135 °C; ^1^H-NMR (500 MHz, CDCl_3_) δ (ppm) = 7.91 (s, 2H, 4-H, 7-H), 7.24 – 7.19 (m, 4H, 5''-H, 6''-H, 7''-H, 8''-H), 4.81 (d, *J* = 16.1 Hz, 1H, 1'-H), 4.01 (d, *J* = 16.1 Hz, 1H, 1'-H), 2.88 (dqd, *J_1_* = 12.5 Hz, *J_2_* = 6.8 Hz, *J_3_* = 2.7 Hz, 1H, 4''-H), 1.86 (dd, *J_1_* = 12.7 Hz, *J_2_* = 2.7 Hz, 1H, 3''-H), 1.66 (s, 3H, 2''-CH_3_), 1.45 (s, 3H, 2''-CH_3_), 1.35 (d, *J* = 6.8 Hz, 3H, 4''-CH_3_), 1.24 (dd, *J_1_* = 12.7 Hz, J_2_ = 12.5 Hz, 1H, 3''-H). ^13^C-NMR (101 MHz, CDCl_3_) δ (ppm) = 166.7 (C-2'), 166.1 (C-1, C-3), 141.8 (C-4a''), 139.0 (C-5, C-6), 137.4 (C-8a''), 131.5 (C-3a, C-7a), 126.6 (C-7''), 126.0 (C-6''), 125.6 (C-4, C-7), 125.1 (C‑8''), 123.7 (C‑5''), 60.4 (C-2''), 51.8 (C-3''), 42.8 (C-1'), 29.4 (C-4''), 27.9 (2''-CH_3_), 25.8 (2''-CH_3_), 17.0 (4''-CH_3_). IR (ATR)
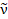
 [cm^-1^] = 3099, 3070, 3028, 2962, 2926, 2878, 2864, 1777, 1715, 1668, 1488, 1416, 1387, 1307, 1247, 1195, 1143, 754. MS (EI) *m/z* (%) = 434 (3) [(^37^Cl_2_) M^•^]^+^, 432 (15) [(^35^Cl, ^37^Cl) M^•^]^+^, 430 (25) [(^35^Cl_2_) M^•^]^+^, 230 (23), 228 (34), 202 (23), 160 (67), 146 (100). HRMS (EI) 430.0866 (calculated for C_22_H_20_^35^Cl_2_N_2_O_3_: 430.0851). Purity (HPLC) 97 % (λ = 210 nm), 97 % (λ = 254 nm).

**(*RS*)-2-[3-Oxo-3-(2,2,4-trimethyl-3,4-dihydroquinolin-1(2*H*)-yl)propyl]isoindoline-1,3-dione (EVP-195)**

C_23_H_24_N_2_O_3_, M_r_ = 376.46

Prepared following general procedure 8 using 3-(1,3-dioxoisoindolin-2-yl)propanoic acid (231 mg, 1.05 mmol), thionyl chloride (0.6 mL) and (*RS*)-2,2,4-trimethyl-1,2,3,4-tetrahydroquinoline (433 mg, 2.47 mmol) to give a colorless solid (89 %). mp: 188 °C; ^1^H-NMR (400 MHz, CDCl_3_) δ (ppm) = 7.82 – 7.75 (m, 2H, 4-H, 7-H), 7.71 – 7.65 (m, 2H, 5-H, 6-H), 7.16 – 7.11 (m, 3H, 5''-H, 6''-H, 7''-H), 6.96 – 6.91 (m, 1H, 8''-H), 4.03 – 3.89 (m, 2H, 1'-H), 2.88 (ddd, *J_1_* = 14.6 Hz, *J_2_* = 8.5 Hz, *J_3_* = 6.0 Hz, 1H, 2'-H), 2.74 – 2.61 (m, 2H, 2'-H, 4''-H), 1.79 (dd, *J_1_* = 12.8 Hz, *J_2_* = 2.6 Hz, 1H, 3''-H), 1.63 (s, 3H, 2''-CH_3_), 1.45 (s, 3H, 2''-CH_3_), 1.27 (d, *J* = 6.8 Hz, 3H, 4''-CH_3_), 1.16 (dd, *J_1_* = 12.8 Hz, *J_2_* = 12.5 Hz, 1H, 3''-H). ^13^C-NMR (101 MHz, CDCl_3_) δ (ppm) = 171.7 (C-3'), 168.1 (C-1, C-3), 141.3 (C-4a''), 138.6 (C-8a''), 134.0 (C-5, C-6), 132.3 (C-3a, C-7a), 126.1 (C-7''), 125.3 (C-6''), 125.2 (C-8''), 123.3 (C-4, C-7), 123.1 (C‑5''), 59.3 (C-2''), 52.4 (C-3''), 35.9 (C-2'), 35.6 (C-1'), 29.2 (C-4''), 27.8 (2''-CH_3_), 25.9 (2''-CH_3_), 16.9 (4''-CH_3_). IR (ATR)
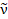
 [cm^-1^] = 3052, 3019, 2991, 2980, 2949, 2933, 1770, 1700, 1651, 1411, 1366, 1346, 1228, 1003, 760, 752, 714. MS (EI) *m/z* (%) = 376 (20) [M^•^]^+^, 252 (32), 250 (27), 202 (42), 175 (35), 160 (100). HRMS (EI) 376.1787 (calculated for C_23_H_24_N_2_O_3_: 376.1787). Purity (HPLC) 98 % (λ = 210 nm), > 99 % (λ = 254 nm).

**(*RS*)-2-[4-Oxo-4-(2,2,4-trimethyl-3,4-dihydroquinolin-1(2*H*)-yl)butyl]isoindoline-1,3-dione (EVP-198)**

C_24_H_26_N_2_O_3_, M_r_ = 390.48

Prepared following general procedure 8 using 4-(1,3-dioxoisoindolin-2-yl)butanoic acid (555 mg, 2.38 mmol), thionyl chloride (2.0 mL) and (*RS*)-2,2,4-trimethyl-1,2,3,4-tetrahydroquinoline (761 mg, 4.34 mmol) to give a colorless solid (78 %). mp: 116 °C; ^1^H-NMR (400 MHz, CDCl_3_) δ (ppm) = 7.81 – 7.76 (m, 2H, 4-H, 7-H), 7.71 – 7.65 (m, 2H, 5-H, 6-H), 7.14 – 7.09 (m, 1H, 5''-H), 7.05 – 7.00 (m, 2H, 6''-H, 7''-H), 6.89 – 6.83 (m, 1H, 8''-H), 3.61 (t, *J* = 7.2 Hz, 2H, 1'-H), 2.79 (dqd, *J_1_* = 12.4 Hz, *J_2_* = 6.7 Hz, *J_3_* = 2.4 Hz, 1H, 4''-H), 2.46 (ddd, *J_1_* = 14.8 Hz, J_2_ = 8.9 Hz, J_3_ = 6.1 Hz, 1H, 3'-H), 2.36 – 2.26 (m, 1H, 3'-H), 2.05 – 1.88 (m, 2H, 2'-H), 1.85 – 1.79 (m, 1H, 3''-H), 1.69 (s, 3H, 2''-CH_3_), 1.46 (s, 3H, 2''-CH_3_), 1.31 (d, *J* = 6.7 Hz, 3H, 4''-CH_3_), 1.18 (dd, *J_1_* = 12.5 Hz, *J_2_* = 12.5 Hz, 1H, 3''-H). ^13^C-NMR
(101 MHz, CDCl_3_) δ (ppm) = 174.1 (C-4'), 168.3 (C-1, C-3), 141.2 (C-4a''), 139.0 (C-8a''), 133.9 (C-5, C-6), 132.2 (C-3a, C-7a), 125.8 (C-7''), 125.1 (C-6''), 124.9 (C-8''), 123.2 (C-4, C-7), 123.0 (C‑5''), 59.2 (C-2''), 52.5 (C-3''), 37.5 (C-1'), 35.1 (C-3'), 29.1 (C-4''), 27.9 (2''-CH_3_), 25.9 (C‑2'), 25.9 (2''-CH_3_), 17.0 (4''-CH_3_). IR (ATR)
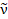
 [cm^-1^] = 3059, 3038, 3006, 2979, 2961, 2944, 2929, 2876, 2862, 1765, 1712, 1659, 1395, 1360, 760, 723. MS (EI) *m/z* (%) = 390 (22) [M^•^]^+^, 216 (34), 175 (72), 160 (100). HRMS (EI) 390.1956 (calculated for C_24_H_26_N_2_O_3_: 390.1944). Purity (HPLC) 98 % (λ = 210 nm), > 99 % (λ = 254 nm).

**(*RS*)-4-Nitro-2-[2-oxo-2-(2,2,4-trimethyl-3,4-dihydroquinolin-1(2*H*)-yl)ethyl]isoindoline-1,3-dione (EVP-199)**

C_22_H_21_N_3_O_5_, M_r_ = 407.43

Prepared following general procedure 7 using (*RS*)-2-bromo-1-(2,2,4-trimethyl-3,4-dihydroquinolin-1(2*H*)-yl)ethan-1-one **(EVP-175)** (266 mg, 0.898 mmol) and 3-nitrophthalimide (190 mg, 0.989 mmol). FCC using hexanes/ethyl acetate 4:1 gave pale yellow crystals (55 %). mp: 177 °C; ^1^H-NMR (500 MHz, CDCl_3_) δ (ppm) = 8.13 – 8.09 (m, 2H, 5-H, 7-H), 7.90 (dd, *J_1_* = 7.8 Hz, *J_2_* = 7.8 Hz, 1H, 6-H), 7.25 – 7.18 (m, 4H, 5''-H, 6''-H, 7''-H, 8''-H), 4.86 (d, *J* = 16.2 Hz, 1H, 1'-H), 4.08 (d, *J* = 16.2 Hz, 1H, 1'-H), 2.89 (dqd, *J_1_* = 12.6 Hz, *J_2_* = 6.8 Hz, *J_3_* = 2.6 Hz, 1H, 4''-H), 1.86 (dd, *J_1_* = 12.7 Hz, *J_2_* = 2.7 Hz, 1H, 3''-H), 1.67 (s, 3H, 2''-CH_3_), 1.45 (s, 3H, 2''-CH_3_), 1.35 (d, *J* = 6.8 Hz, 3H, 4''-CH_3_), 1.23 (dd, *J_1_* = 12.5 Hz, *J_2_* = 12.7 Hz, 1H, 3''-H). ^13^C-NMR (101 MHz, CDCl_3_) δ (ppm) = 166.5 (C-2'), 165.6 (C-1), 162.6 (C-3), 145.3 (C-4), 141.8 (C-4a''), 137.4 (C-8a''), 135.4 (C-6), 134.5 (C-7a), 128.7 (C-5), 127.3 (C-7''), 126.6 (C-7), 126.1 (C-6''), 125.2 (C-8''), 124.2 (C-3a), 123.7 (C-5''), 60.5 (C-2''), 51.8 (C-3''), 42.9 (C-1'), 29.3 (C-4''), 27.9 (2''-CH_3_), 25.8 (2''-CH_3_), 17.0 (4''-CH_3_). IR (ATR)
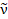
 [cm^-1^] = 3096, 3027, 2979, 2959, 2944, 2921, 1733, 1676, 1540, 1419, 1391, 1371, 1308, 1251, 1114, 769, 746, 716. MS (EI) *m/z* (%) = 407 (48) [M^•^]^+^, 186 (50), 160 (100), 146 (85), 128 (33). HRMS (EI) 407.1486 (calculated for C_22_H_21_N_3_O_5_: 407.1481). Purity (HPLC) 98 % (λ = 210 nm), 98 % (λ = 254 nm).

**(*RS*)-5-Nitro-2-[2-oxo-2-(2,2,4-trimethyl-3,4-dihydroquinolin-1(2*H*)-yl)ethyl]isoindoline-1,3-dione (EVP-200)**

C_22_H_21_N_3_O_5_, M_r_ = 407.43

Prepared following general procedure 7 using (*RS*)-2-bromo-1-(2,2,4-trimethyl-3,4-dihydroquinolin-1(2*H*)-yl)ethan-1-one **(EVP-175)** (208 mg, 0.702 mmol) and 4-nitrophthalimide (167 mg, 0.869 mmol). FCC using hexanes/ethyl acetate 9:1 gave yellow crystals (79 %). mp: 108 °C; ^1^H-NMR (500 MHz, CDCl_3_) δ (ppm) = 8.64 – 8.61 (m, 1H, 4-H), 8.58 (dd, *J_1_* = 8.1 Hz, *J_2_* = 1.9 Hz, 1H, 6-H), 8.02 (d, *J* = 8.1 Hz, 1H, 7-H), 7.25 – 7.18 (m, 4H, 5''-H, 6''-H, 7''-H, 8''-H), 4.87 (d, *J* = 16.1 Hz, 1H, 1'-H), 4.06 (d, *J* = 16.1 Hz, 1H, 1'-H), 2.88 (dqd, *J_1_* = 12.8 Hz, *J_2_* = 6.6 Hz, *J_3_* = 2.5 Hz, 1H, 4''-H), 1.86 (dd, *J_1_* = 13.0 Hz, *J_2_* = 2.6 Hz, 1H, 3''-H), 1.65 (s, 3H, 2''-CH_3_), 1.44 (s, 3H, 2''-CH_3_), 1.35 (d, *J* = 6.7 Hz, 3H, 4''-CH_3_), 1.23 (dd, *J_1_* = 13.0 Hz, *J_2_* = 12.6 Hz, 1H, 3''-H). ^13^C-NMR (101 MHz, CDCl_3_) δ (ppm) = 166.4 (C-2'), 165.8 (C-1), 165.6 (C-3), 151.8 (C-5), 141.7 (C-4a''), 137.3 (C-8a''), 136.7 (C-7a), 133.7 (C-3a), 129.3 (C-6), 126.5 (C-7''), 126.1 (C-6''), 125.1 (C-8''), 124.7 (C-7), 123.6 (C-5''), 118.8 (C-4), 60.4 (C-2''), 51.7 (C-3''), 42.8 (C-1'), 29.3 (C-4''), 27.8 (2''-CH_3_), 25.7 (2''-CH_3_), 17.0 (4''-CH_3_). IR (ATR)
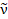
 [cm^-1^] = 3077, 3045, 2980, 2933, 2890, 1721, 1670, 1540, 1418, 1393, 1345, 1307, 1246, 1195, 1107, 763, 718. MS (EI) *m/z* (%) = 407 (40) [M^•^]^+^, 252 (23), 205 (43), 160 (90), 146 (100), 128 (27). HRMS (EI) 407.1497 (calculated for C_22_H_21_N_3_O_5_: 407.1481). Purity (HPLC) 98 % (λ = 210 nm), 98 % (λ = 254 nm).

**(*RS*)-4,5,6,7-Tetrafluoro-2-[2-oxo-2-(2,2,4-trimethyl-3,4-dihydroquinolin-1(2*H*)-yl)ethyl] isoindoline-1,3-dione (EVP-203)**

C_22_H_18_F_4_N_2_O_3_, M_r_ = 434.39

Prepared following general procedure 8 using 2-(4,5,6,7-tetrafluoro-1,3-dioxoisoindolin-2-yl)acetic acid (285 mg, 1.03 mmol), thionyl chloride (2 mL) and (*RS*)-2,2,4-trimethyl-1,2,3,4-tetrahydroquinoline (336 mg, 1.92 mmol) to give colorless crystals (57 %). mp: 221 °C; ^1^H-NMR (400 MHz, CDCl_3_) δ (ppm) = 7.26 – 7.15 (m, 4H, 5''-H, 6''-H, 7''-H, 8''-H), 4.81 (d, *J* = 16.2 Hz, 1H, 1'-H), 3.99 (d, *J* = 16.2 Hz, 1H, 1'-H), 2.88 (dqd, *J_1_* = 13.4 Hz, *J_2_* = 6.8 Hz, *J_3_* = 2.6 Hz, 1H, 4''‑H), 1.87 (dd, *J_1_* = 12.8 Hz, *J_2_* = 2.7 Hz, 1H, 3''-H), 1.66 (s, 3H, 2''-CH_3_), 1.45 (s, 3H, 2''-CH_3_), 1.36 (d, *J* = 6.8 Hz, 3H, 4''-CH_3_), 1.24 (dd, *J_1_* = 13.3 Hz, *J_2_* = 12.7 Hz, 1H, 3''). ^13^C-NMR (101 MHz, CDCl_3_) δ (ppm) = 166.0 (C-1, C-3), 162.1 (C-2'), 145.0 (d, *J* = 269 Hz, C-5, C-6), 143.4 (d, *J* = 262 Hz, C-4, C-7), 141.7 (C-4a''), 137.2 (C-8a''), 126.5 (C-7''), 126.1 (C-6''), 125.0 (C‑8''), 123.6 (C-5''), 114.0 (C-3a, C-7a), 60.5 (C-2''), 51.6 (C-3''), 42.7 (C-1'), 29.2 (C‑4''), 27.7 (2''-CH_3_), 25.6 (2''-CH_3_), 16.9 (4''-CH_3_). IR (ATR)
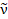
 [cm^-1^] = 3071, 3040, 3021, 2999, 2980, 2959, 2933, 2922, 2866, 1725, 1667, 1513, 1499, 1423, 968. MS (EI) *m/z* (%) = 434 (55) [M^•^]^+^, 419 (27), 232 (72), 202 (27), 160 (100), 146 (90), 128 (27). HRMS (EI) 434.1285 (calculated for C_22_H_18_F_4_N_2_O_3_: 434.1254). Purity (HPLC) 99 % (λ = 210 nm), > 99 % (λ = 254 nm)

**(3a*RS*,4*SR*,7*RS*,7a*RS*)-3-Mesityl-3a,4,7,7a-tetrahydro-4,7-methanobenzo[*d*]isoxazole (EVP-204)** C_17_H_19_NO, M_r_ = 253.35, **(3a*RS*,4*RS*,4a*RS*,7a*RS*,8*RS*,8a*RS*)-3,7-dimesityl-3a,4,4a,7a,8,8a-hexahydro-4,8-methanobenzo[1,2-*d*:4,5-*d'*]diisoxazole (EVP-204_A)** C_27_H_30_N_2_O_2_, M_r_ = 414.55 and ***meso*-(3a*R*,4*S*,4a*S*,7a*R*,8*S*,8a*S*)-3,5-dimesityl-3a,4,4a,7a,8,8a-hexahydro-4,8-methanobenzo[1,2-*d*:5,4-*d'*]diisoxazole (EVP-204_B)** C_27_H_30_N_2_O_2_, M_r_ = 414.55

Prepared following general procedure 3a using mesitonitrile oxide (364 mg, 2.26 mmol) and norbornadiene (227 mg, 2.46 mmol). FCC using a gradient hexane/ethyl acetate 97:3 -> 4:1 was used to separate the monoadduct **EVP-204** (53 %) (eluting first) from the two isomeric diadducts **EVP-204_A** (21 %) (eluting second) and **EVP-204_B** (8 %) (eluting third).

**EVP-204:** colorless crystals (53 %). mp: 128 °C; ^1^H-NMR (400 MHz, CDCl_3_) δ (ppm) = 6.92 – 6.88 (m, 2H, 3'-H, 5'-H), 6.19 (dd, *J_1_* = 5.8 Hz, *J_2_* = 3.0 Hz, 1H, 5-H), 6.08 (dd, *J_1_* = 5.8 Hz, *J_2_* = 3.1 Hz, 1H, 6-H), 5.02 (br.d, *J* = 8.2 Hz, 1H, 7a-H), 3.56 (br.d, *J* = 8.2 Hz, 1H, 3a-H), 3.32 (ddd, *J_1_* = 3.1 Hz, *J_2_* = 1.5 Hz, *J_3_* = 1.5 Hz, 1H, 7-H), 2.93 – 2.88 (m, 1H, 4-H), 2.29 (s, 3H, 4'-CH_3_), 2.28 (s, 6H, 2'-CH_3_, 6'‑CH_3_), 2.07 (ddd, *J_1_* = 9.2 Hz, *J_2_* = 1.6 Hz, *J_3_* = 1.6 Hz, 1H, 8-H), 1.74 (ddd, *J_1_* = 9.3 Hz, *J_2_* = 1.6 Hz, *J_3_* = 1.6 Hz, 1H, 8‑H). ^13^C-NMR (126 MHz, CDCl_3_) δ (ppm) = 157.1 (C-3), 140.4 (C-5), 138.7 (C-4'), 136.8 (C-2', C-6'), 136.0 (C-6), 128.9 (C-3', C-5'), 126.4 (C-1'), 88.5 (C-7a), 62.5 (C-3a), 49.7 (C-7), 44.9 (C-4), 43.7 (C-8), 21.2 (4'-CH_3_), 20.3 (2'-CH_3_, 6'-CH_3_). IR (ATR)
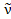
 [cm^-1^] = 3067, 3046, 3003, 2980, 2970, 2952, 2924, 2891, 1455, 1323, 902, 858, 714. MS (EI) *m/z* (%) = 253 (63) [M^•^]^+^, 188 (55), 187 (66), 158 (100), 144 (50). HRMS (EI) 253.1470 (calculated for C_17_H_19_NO: 253.1467). Purity (HPLC) > 99 % (λ = 210 nm), > 99 % (λ = 254 nm).

By-products:

**EVP-204_A:** colorless crystals (21 %). mp: - decomposition: 293 °C; ^1^H-NMR (500 MHz, CDCl_3_) δ (ppm) = 6.92 (s, 4H, 3'-H, 5'-H, 3''-H, 5''-H), 4.68 (d, *J* = 8.0 Hz, 2H, 4a-H, 8a-H), 3.39 (d, *J* = 8.1 Hz, 2H, 3a-H, 7a-H), 2.73 (s, 2H, 4-H, 8-H), 2.30 (s, 6H, 4'-CH_3_, 4''-CH_3_), 2.28 (s, 12H, 2'-CH_3_, 6'-CH_3_, 2''-CH_3_, 6''-CH_3_), 2.02 (s, 2H, 9-H). ^13^C-NMR (126 MHz, CDCl_3_) δ (ppm) = 156.7 (C-3, C-7), 139.0 (C-4', C-4''), 136.6 (C-2', C-6', C-2'', C-6''), 128.9 (C-3', C-5', C-3'', C-5''), 125.3 (C-1', C-1''), 85.5 (C-4a, C-8a), 57.2 (C-3a, C-7a), 45.4 (C-4, C-8), 27.6 (C-9), 21.1 (4'-CH_3_, 4''-CH_3_), 20.1 (2'-CH_3_, 6'-CH_3_, 2''-CH_3_, 6''-CH_3_). IR (ATR)
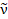
 [cm^-1^] = 3104, 3024, 2980, 2919, 2884, 1611, 1458, 1432, 1371, 1055, 951, 922, 898, 859, 848, 831. MS (EI) *m/z* (%) = 414 (24) [M^•^]^+^, 158 (22), 145 (57), 144 (22), 130 (100). HRMS (EI) 414.2308 (calculated for C_27_H_30_N_2_O_2_: 414.2307). Purity (HPLC) 98 % (λ = 210 nm), > 99 % (λ = 254 nm).

**EVP-204_B:** colorless crystals (8 %). mp: 230 °C; ^1^H-NMR (500 MHz, CDCl_3_) δ (ppm) = 6.86 (s, 4H, 3'-H, 5'-H, 3''-H, 5''-H), 4.77 (d, *J* = 8.3 Hz, 2H, 7a-H, 8a-H), 3.38 (d, *J* = 8.4 Hz, 2H, 3a-H, 4a-H), 3.24 – 3.17 (m, 1H, 8-H), 2.32 – 2.29 (m, 1H, 4-H), 2.25 (s, 6H, 4'-CH_3_, 4''-CH_3_), 2.22 (s, 12H, 2'-CH_3_, 6'-CH_3_, 2''-CH_3_, 6''-CH_3_), 2.02 – 1.96 (m, 2H, 9-H). ^13^C-NMR (126 MHz, CDCl_3_) δ (ppm) = 156.7 (C-3, C-5), 139.1 (C-4', C-4''), 136.6 (C-2', C-6', C-2'', C-6''), 129.0 (C‑3', C-5', C-3'', C-5''), 125.4 (C-1', C-1''), 82.9 (C-7a, C-8a), 61.3 (C-3a, C-4a), 50.6 (C-8), 41.4 (C-4), 28.1 (C-9), 21.1 (4'-CH_3_, 4''-CH_3_), 20.2 (2'-CH_3_, 6'-CH_3_, 2''-CH_3_, 6''-CH_3_). IR (ATR)
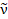
 [cm^-1^] = 3085, 3070, 3006, 2958, 2920, 2858, 1731, 1515, 1501, 1424, 859. MS (EI) *m/z* (%) = 414 (42) [M^•^]^+^, 210 (35), 158 (52), 145 (65), 130 (100). HRMS (EI) 414.2309 (calculated for C_27_H_30_N_2_O_2_: 414.2307). Purity (HPLC) 88 % (λ = 210 nm), 95 % (λ = 254 nm).

**(3a*S*,5*S*,7*S*,7a*S*)-3-Mesityl-6,6,7a-trimethyl-3a,4,5,6,7,7a-hexahydro-5,7-methanobenzo[*d*]isoxazole (EVP-205)**

C_20_H_27_NO, M_r_ = 297.44

Prepared following general procedure 3a using mesitonitrile oxide (496 mg, 3.08 mmol) and (+)‑α‑pinene (645 mg, 4.73 mmol). FCC using hexanes/ethyl acetate 97:3 gave colorless crystals (39 %). mp: 141 °C; ^1^H-NMR (400 MHz, CDCl_3_) δ (ppm) = 6.95 – 6.83 (m, 2H, 3'-H, 5'-H), 3.34 (dd, *J_1_* = 11.3 Hz, *J_2_* = 3.8 Hz, 1H, 3a-H), 2.33 (s, 6H, 2'-CH_3_, 6'-CH_3_), 2.31 – 2.26 (m, 1H, 8-H), 2.29 (s, 3H, 4'-CH_3_), 2.19 (dd, *J_1_* = 6.1 Hz, *J_2_* = 4.8 Hz, 1H, 7-H), 1.98 – 1.92 (m, 1H, 5-H), 1.87 (dddd, *J_1_* = 13.7 Hz, *J_2_* = 11.3 Hz, *J_3_* = 2.2 Hz, *J_4_* = 2.1 Hz, 1H, 4-H), 1.63 (ddd, *J_1_* = 13.9 Hz, *J_2_* = 3.8 Hz, *J_3_* = 3.6 Hz, 1H, 4-H), 1.49 (s, 3H, 7a-CH_3_), 1.39 (d, *J* = 10.7 Hz, 1H, 8-H), 1.31 (s, 3H, 6-CH_3_), 0.92 (s, 3H, 6-CH_3_). ^13^C-NMR (101 MHz, CDCl_3_) δ (ppm) = 159.5 (C-3), 138.6 (C-4'), 137.2 (C-2', C-6'), 128.9 (C-3', C-5'), 126.4 (C-1'), 90.2 (C-7a), 50.4 (C-7), 49.2 (C-3a), 39.5 (C-5), 37.8 (C-6), 27.9 (7a-CH_3_), 27.8 (C-4), 27.0 (6-CH_3_), 26.3 (C-8), 23.4 (6-CH_3_), 21.2 (4'-CH_3_), 20.6 (2'-CH_3_, 6'-CH_3_). IR (KBr)
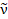
 [cm^-1^] = 3001, 2997, 2985, 2976, 2926, 2869, 1474, 1458, 1446, 1373, 1322, 924, 885, 866. MS (EI) *m/z* (%) = 297 (100) [M^•^]^+^, 282 (100), 254 (27), 146 (60), 145 (25), 119 (30). HRMS (EI) 297.2064 (calculated for C_20_H_27_NO: 297.2093). Purity (HPLC) > 99 % (λ = 210 nm), > 99 % (λ = 254 nm).

**(*RS*)-2-[5-Oxo-5-(2,2,4-trimethyl-3,4-dihydroquinolin-1(2*H*)-yl)pentyl]isoindoline-1,3-dione (EVP-207)**

C_25_H_28_N_2_O_3_, M_r_ = 404.51

Prepared following general procedure 8 using 5-(1,3-dioxoisoindolin-2-yl)pentanoic acid (504 mg, 2.04 mmol), thionyl chloride (2 mL) and (*RS*)-2,2,4-trimethyl-1,2,3,4-tetrahydroquinoline (530 mg, 3.02 mmol) to give pale yellow oil (66 %); ^1^H-NMR (500 MHz, CDCl_3_) δ (ppm) = 7.81 – 7.69 (m, 2H, 4-H, 7-H), 7.69 – 7.57 (m, 2H, 5-H, 6-H), 7.13 – 6.99 (m, 3H, 5''-H, 6''-H, 7''-H), 6.83 (d, *J* = 6.9 Hz, 1H, 8''-H), 3.52 (t, *J* = 6.3 Hz, 2H, 1'-H), 2.75 – 2.64 (m, 1H, 4''-H), 2.44 – 2.32 (m, 1H, 4'-H), 2.32 – 2.21 (m, 1H, 4'-H), 1.76 (d, *J* = 12.8 Hz, 1H, 3''-H), 1.61 (s, 3H, 2''-CH_3_), 1.60 – 1.46 (m, 4H, 2'-H, 3'-H), 1.41 (s, 3H, 2''-CH_3_), 1.25 (d, *J* = 6.8 Hz, 3H, 4''-CH_3_), 1.12 (dd, *J_1_* = 12.8 Hz, *J_2_* = 12.5 Hz, 1H, 3''-H). ^13^C-NMR (126 MHz, CDCl_3_) δ (ppm) = 174.6 (C-5'), 168.1 (C-1, C-3), 140.8 (C-4a''), 138.9 (C-8a''), 133.8 (C-5, C-6), 132.0 (C-3a, C-7a), 125.7 (C-7''), 124.9 (C-6''), 124.7 (C-8''), 123.0 (C-4, C-7), 122.8 (C‑5''), 58.8 (C-2''), 52.2 (C-3''), 37.6 (C-1'), 37.0 (C-4'), 28.9 (C-4''), 27.8 (C-3'), 27.7 (2''-CH_3_), 25.7 (2''-CH_3_), 24.0 (C-2'), 16.8 (4''-CH_3_). IR (ATR)
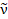
 [cm^-1^] = 2980, 2927, 2870, 1771, 1714, 1659, 1396, 1373, 1040, 721. MS (EI) *m/z* (%) = 404 (15) [M^•^]^+^, 230 (14), 175 (66), 160 (100). HRMS (EI) 404.2102 (calculated for C_25_H_28_N_2_O_3_: 404.2100). Purity (HPLC) 99 % (λ = 210 nm), > 99 % (λ = 254 nm).

**(*RS*)-2-[6-Oxo-6-(2,2,4-trimethyl-3,4-dihydroquinolin-1(2*H*)-yl)hexyl]isoindoline-1,3-dione (EVP-209)**

C_26_H_30_N_2_O_3_, M_r_ = 418.54

Prepared following general procedure 8 using 6-(1,3-dioxoisoindolin-2-yl)hexanoic acid (509 mg, 1.95 mmol), thionyl chloride (2 mL) and (*RS*)-2,2,4-trimethyl-1,2,3,4-tetrahydroquinoline (514 mg, 2.93 mmol) to give a colorless oil (65 %); ^1^H-NMR (500 MHz, CDCl_3_) δ (ppm) = 7.84 – 7.79 (m, 2H, 4-H, 7-H), 7.73 – 7.66 (m, 2H, 5-H, 6-H), 7.18 – 7.15 (m, 1H, 5''-H), 7.14 – 7.09 (m, 2H, 6''-H, 7''-H), 6.90 – 6.85 (m, 1H, 8''-H), 3.61 (t, *J* = 7.2 Hz, 2H, 1'-H), 2.79 – 2.67 (m, 1H, 4''-H), 2.38 (ddd, *J_1_* = 14.3 Hz, *J_2_* = 8.2 Hz, *J_3_* = 6.1 Hz, 1H, 5'-H), 2.31 – 2.19 (m, 1H, 5'-H), 1.81 (dd, *J_1_* = 12.8 Hz, *J_2_* = 2.7 Hz, 1H, 3''), 1.63 (s, 3H, 2''-CH_3_), 1.67 – 1.54 (m, 4H, 2'-H, 4'-H), 1.46 (s, 3H, 2''-CH_3_), 1.32 (d, *J* = 6.8 Hz, 3H, 4''-CH_3_), 1.30 – 1.21 (m, 2H, 3'-H), 1.21 – 1.15 (m, 1H, 3''-H). ^13^C-NMR (126 MHz, CDCl_3_) δ (ppm) = 175.3 (C-6'), 168.5 (C-1, C-3), 141.0 (C-4a''), 139.3 (C-8a''), 133.9 (C-5, C-6), 132.3 (C-3a, C-7a), 125.9 (C-7''), 125.2 (C-6''), 124.8 (C-8''), 123.3 (C-4, C-7), 122.9 (C-5''), 59.0 (C-2''), 52.5 (C-3''), 38.0 (C-1'), 37.6 (C-5'), 29.2 (C-4''), 28.5 (C-2'), 27.9 (2''‑CH_3_), 26.7 (C-4'), 26.5 (C-3'), 25.9 (2''-CH_3_), 17.0 (4''-CH_3_). IR (ATR)
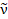
 [cm^-1^] = 3029, 2996, 2981, 2933, 2865, 1772, 1716, 1661, 1396, 1363, 1049, 722. MS (EI) *m/z* (%) = 418 (20) [M^•^]^+^, 270 (26), 175 (90), 160 (100). HRMS (EI) 418.2263 (calculated for C_26_H_30_N_2_O_3_: 418.2257). Purity (HPLC) > 99 % (λ = 210 nm), > 99 % (λ = 254 nm).

**Supplementary References**

Caldirola, P., Ciancaglione, M., De Amici, M. & De Micheli, C. Conversion of isoxazolines to β-hydroxy esters. Synthesis of 2-deoxy-D-ribose. *Tetrahedron Letters* 27, 4647-4650 (1986).

Chen, R. *et al.* Effect of Tetrahydroquinoline Dyes Structure on the Performance of Organic Dye-Sensitized Solar Cells. *Chemistry of Materials* 19, 4007-4015 (2007).

Chen, C.C. *et al.* [A small molecule restores function to TRPML1 mutant isoforms responsible for mucolipidosis type IV.](https://www.ncbi.nlm.nih.gov/pubmed/25119295) *Nat. Commun.* 5, 4681 (2014).

Chen, C.C. *et al.* [Small Molecules for Early Endosome-Specific Patch Clamping.](https://www.ncbi.nlm.nih.gov/pubmed/28732201) *Cell Chem. Biol.* 24, 907-916.e4 (2017).

[Cheng, X](https://www.ncbi.nlm.nih.gov/pubmed/?term=Cheng X%5BAuthor%5D&cauthor=true&cauthor_uid=25216637). *et al.* The intracellular Ca²⁺ channel MCOLN1 is required for sarcolemma repair to prevent muscular dystrophy. [*Nat. Med.*](https://www.ncbi.nlm.nih.gov/pubmed/25216637) 20, 1187-1192 (2014).

[Dong, X.P](https://www.ncbi.nlm.nih.gov/pubmed/?term=Dong XP%5BAuthor%5D&cauthor=true&cauthor_uid=20802798). *et al.* PI(3,5)P(2) controls membrane trafficking by direct activation of mucolipin Ca(2+) release channels in the endolysosome. [*Nat. Commun.*](https://www.ncbi.nlm.nih.gov/pubmed/?term=Dong+2010+TRPML) 1, 38 (2010).

Grimm, C. Endolysosomal cation channels as therapeutic targets – Pharmacology of TRPML channels. *Messenger* 5, 30–36 (2016).

Grimm, C. *et al.* Small Molecule Activators of TRPML3. *Chemistry & Biology* 17, 135-148 (2010).
