# Supplementary material for: Selective agonist of TRPML2 reveals direct role in chemokine release from innate immune cells
Source: eLife. 2018 Nov 27;7:e39720. doi: 10.7554/eLife.39720 (PMC6257821; doi:10.7554/eLife.39720)
Supplement: Supplementary file 2. [file elife-39720-supp2.docx]

**Supplementary file 2.** Characteristics of TRPML channels

|  | **TRPML1** | **TRPML2** | **TRPML3** | **Ref.** |
| --- | --- | --- | --- | --- |
| **Luminal pH for max. activity** | Acidic | Neutral | Neutral | (Dong et al, 2010; Chen et al, 2017) |
| **Endogenous agonist** | PI(3,5)P_2_ | PI(3,5)P_2_ | PI(3,5)P_2_ | (Dong et al, 2010; Chen et al, 2017) |
| **Endogenous antagonist** | PI(4,5)P_2_, PI(3,4)P_2_, PI(3,4,5)P_3_ | ? | PI(4,5)P_2_ | (Zhang et al, 2012; Chen et al, 2017) |
| **Specific small molecule agonists** | None | ML2-SA1 = EVP-22(h,m) | e.g. EVP-21(h) | (Grimm et al, 2010; Grimm et al, 2016; Plesch et al) |
| **ROS sensitivity** | Yes | No | No | (Zhang et al, 2016) |
| **LPS sensitivity** | No effect | Up-regulation | No effect | (Sun et al, 2015; Plech et al) |
| **Channel activity (organelle, EL patch clamp)** | LE/LY | LE/LY, EE, RE, Rab11+, Tf/TfR+ | LE/LY, EE | (Chen et al, 2017; Plesch et al) |
| **Endogenous channel activity (EL patch clamp)** | AMΦ, BMMΦ, PMΦ, LMΦ, fibroblasts, myoblasts, parietal cells, hippo-campal neurons (unpubl.), RPE (unpubl.) | Activated (e.g. LPS) AMΦ, BMMΦ | AMΦ, LMΦ, SMΦ (unpubl.), BEC | (Shen et al, 2012; Cheng et al, 2014; Miao et al, 2015; Chen et al, 2017; Sahoo et al, 2017; Plesch et al) |
